# Design and synthesis of 1,3,4-oxadiazole-tethered *N*-substituted isatin hybrids as SARS-CoV-2 main protease inhibitors: biological evaluation, kinetic investigation, and *in silico* studies

**DOI:** 10.1039/d6ra06004h

**Published:** 2026-09-01

**Authors:** Tarfah Al-Warhi, Md Sofequl Islam Mukim, Zainab M. Elsayed, Yasser M. Omar, Diaaeldin M. Elimam, Moataz A. Shaldam, Ashraf K. El-Damasy, Mostafa M. Elbadawi, Haytham O. Tawfik, Dae-Geun Song, Wagdy M. Eldehna

**Affiliations:** a Department of Chemistry, College of Science, Princess Nourah Bint Abdulrahman University P.O. Box 84428 Riyadh 11671 Saudi Arabia; b Center for Natural Product Systems Biology, KIST Gangneung Institute of Natural Products Gangneung 25451 Republic of Korea dsong82@kist.re.kr; c Natural Product Applied Science, KIST School, University of Science and Technology Gangneung 25451 Republic of Korea; d Scientific Research and Innovation Support Unit, Faculty of Pharmacy, Kafrelsheikh University Kafr El-Shaikh 33516 Egypt; e Institute of Cancer Therapeutics, University of Bradford BD7 1DP UK; f Department of Pharmaceutical Organic Chemistry, Faculty of Pharmacy, Assiut University Assiut 71526 Egypt; g Department of Pharmacognosy, Faculty of Pharmacy, Kafrelsheikh University Kafrelsheikh 33516 Egypt; h Department of Pharmaceutical Chemistry, Faculty of Pharmacy, Kafrelsheikh University Kafrelsheikh P.O. Box 33516 Egypt wagdy2000@gmail.com; i Department of Pharmaceutical Chemistry, Faculty of Pharmacy, Alsalam University 31511 Tanta Egypt haytham.omar.mahmoud@pharm.tanta.edu.eg; j Department of Medicinal Chemistry, Faculty of Pharmacy, Mansoura University Mansoura 35516 Egypt; k Department of Pharmaceutical Chemistry, Faculty of Pharmacy, Tanta University Tanta 31527 Egypt

## Abstract

The ongoing emergence of SARS-CoV-2 mutations underscores the urgent need for new antivirals that target key viral proteins. This study describes the design, synthesis, and evaluation of two series of 1,3,4-oxadiazole-tethered *N*-substituted isatin hybrids as inhibitors of the SARS-CoV-2 main protease (M^pro^): 1,2,3-triazole-linked derivatives (9a–h) and pyrazole-linked derivatives (15a–d). Compounds 15a–c were identified as the most active derivatives in initial FRET-based screening. With an IC_50_ of 15.38 µM, 15c was the most effective inhibitor, as determined by subsequent enzymatic assays. 15a and 15b had IC_50_ values of 24.43 and 30.55 µM, respectively. The active chemicals inhibit M^pro^*via* a noncompetitive/mixed mechanism (*α* = 0.41–0.44), at a *K*_I_ value of 13.24 µM, according to enzyme kinetic studies. Additionally, the compounds exhibited favorable physicochemical and ADMET profiles and minimal cytotoxicity against normal IMR-90 cells. The stable predicted binding of the most active compound, 15c, within the M^pro^ active site was further supported by molecular docking and molecular dynamics simulations. Collectively, these findings identify 15c as the most active derivative in the present series and provide preliminary SAR insights that may guide further optimization of this scaffold as a potential class of SARS-CoV-2 M^pro^ inhibitors.

## Abstract 2

The ongoing emergence of SARS-CoV-2 mutations underscores the urgent need for new antivirals that target key viral proteins.

## Graphical abstract

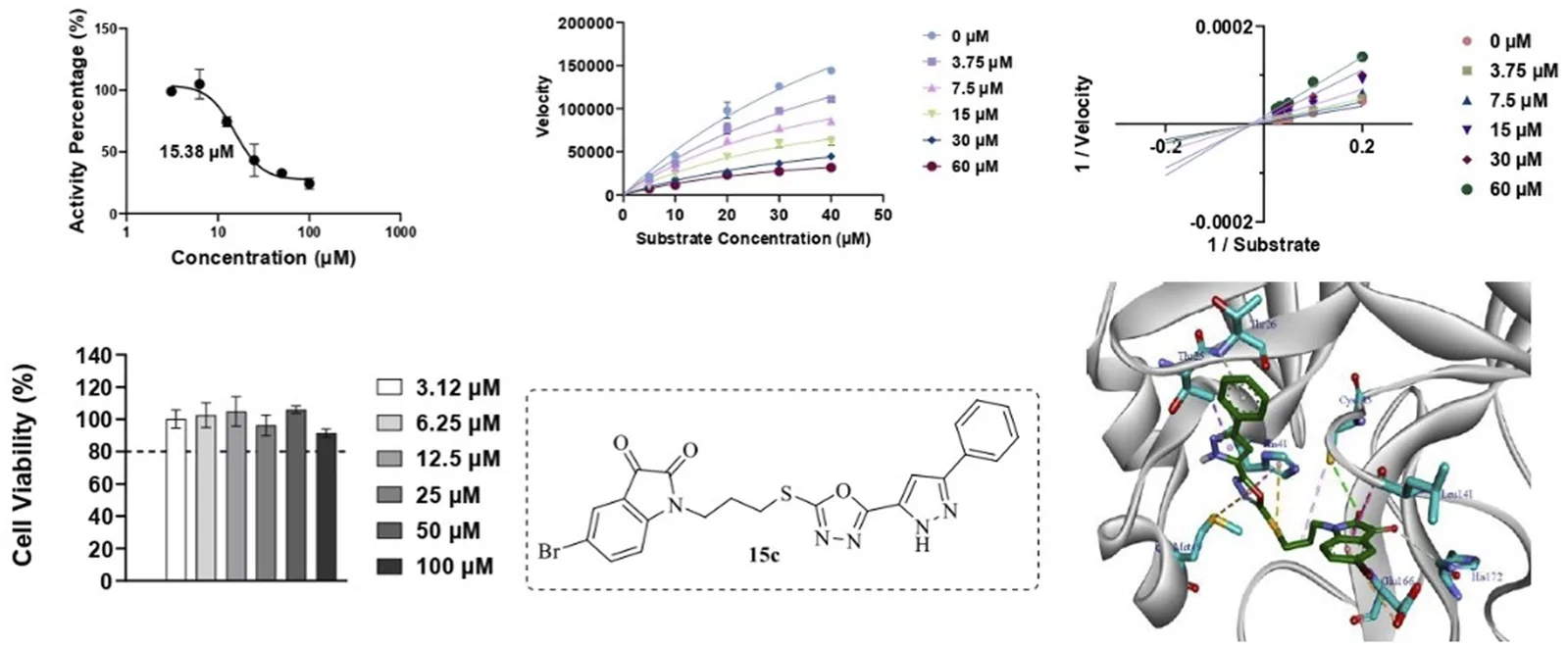

## Introduction

Due to the ongoing emergence of viral variants and the scarcity of highly effective antiviral medications, coronavirus disease 2019 (COVID-19), caused by severe acute respiratory syndrome coronavirus 2 (SARS-CoV-2), remains a serious global health concern.^1^ The replication and transcription of the positive-sense single-stranded RNA virus SARS-CoV-2 rely on an intricate network of viral proteins and enzymes, some of which have become appealing targets for the development of antiviral medications.^2^

One of the most appealing therapeutic targets for anti-SARS-CoV-2 drug discovery among viral proteins is the main protease (M^pro^), also known as non-structural protein 5 (Nsp5) or 3-chymotrypsin-like protease (3CL^pro^).^3,4^ By mediating proteolytic cleavage of the viral polyproteins pp1a and pp1ab, M^pro^ plays a crucial role in the viral life cycle by releasing 13 of the 16 non-structural proteins required for viral transcription and replication.^5^

His41 and Cys145 form the catalytic dyad of M^pro^, which is responsible for substrate recognition and proteolytic cleavage at specific glutamine-containing regions.^6,7^ Crucially, human proteases lack this substrate specificity, enabling the development of selective inhibitors with low off-target effects.^8^ Furthermore, M^pro^ is highly conserved across pathogenic coronaviruses, including SARS-CoV-2, SARS-CoV, and MERS-CoV, and mutations arising in emerging viral strains do not affect its catalytic center.^9^ Because of these special qualities, M^pro^ is a desirable target for both the treatment of COVID-19 and the development of broad-spectrum antiviral drugs to combat current and emerging coronavirus infections.^10^

**Fig. 1:**
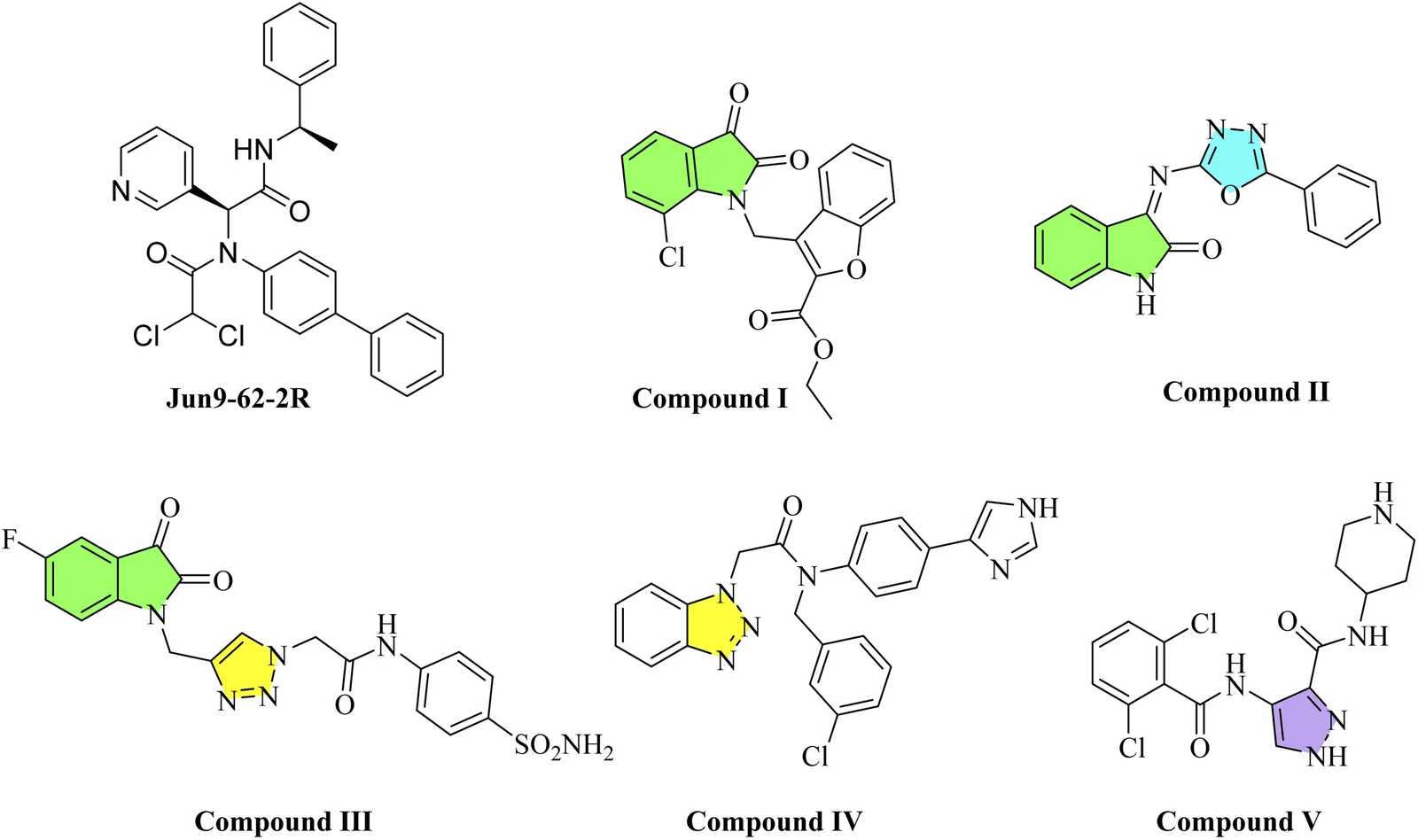
Chemical structures of representative isatin-, 1,3,4-oxadiazole-, 1,2,3-triazole-, and pyrazole-based inhibitors of SARS-CoV-2 M^pro^ together with the peptidomimetic inhibitor Jun9-62-2R.

Numerous substances have been identified as SARS-CoV-2 M^pro^ inhibitors; peptidomimetic inhibitors have shown exceptional potency.^11^ These substances often include electrophilic warheads that can interact covalently with the catalytic residue Cys145 and are typically made to resemble the natural substrates of M^pro^.^12^ Nirmatrelvir, Jun9-62-2R, FB2001, PF-00835231, PF-07304814, and GC376 are a few examples.^13,14^ Notably, Jun9-62-2R inhibited SARS-CoV-2 M^pro^ with an IC_50_ value of 0.43 µM (Fig. 1). Despite their high potency, peptidomimetic inhibitors may exhibit limitations related to their relatively high molecular weight and conformational complexity, including poor membrane permeability, limited metabolic stability, suboptimal oral bioavailability, and unfavorable pharmacokinetic properties.^15^ Consequently, considerable effort has been directed toward developing non-peptidic M^pro^ inhibitors with potent antiviral activity and favorable drug-like properties.^16^

Because of their favorable drug-like properties and structural diversity, non-peptidic inhibitors have become a desirable alternative to peptidomimetic M^pro^ inhibitors.^17^ Trisubstituted piperazines, spiropyrazolopyridones, and flavonoid derivatives like baicalein are among the families of non-peptidic drugs that have shown encouraging inhibitory efficacy against SARS-CoV-2 M^pro^.^18^ Heterocyclic compounds have garnered significant interest among the many scaffolds investigated due to their synthetic versatility, tunable physicochemical properties, and ability to engage in diverse interactions within the M^pro^ binding pocket.^19,20^

Because of its wide range of biological activities, especially its antiviral potential, isatin has become a favored structure among the several heterocyclic scaffolds studied for antiviral drug discovery.^21,22^ Because of their synthetic accessibility, structural adaptability, and ability to form important intermolecular interactions within the enzyme's active site, isatin-based derivatives have garnered significant interest in recent years as potential SARS-CoV-2 M^pro^ inhibitors.^23,24^ Specifically, the carbonyl and NH functionalities of the isatin nucleus can engage in hydrogen-bonding interactions with key residues in the substrate-binding pocket,^23^ whereas *N*-substitution provides an effective means of adjusting both pharmacological and pharmacokinetic properties.^25^

The literature contains a wealth of information about the antiviral potential of isatin-based agents against SARS-CoV-2 M^pro^. Strong inhibitory effects against M^pro^ were demonstrated by representative *N*-substituted isatin derivatives (I^26^ and II,^27^Fig. 1). It's interesting to note that compound II, which contains a 1,3,4-oxadiazole moiety, demonstrated the beneficial contribution of this heterocycle to M^pro^ inhibition, suggesting the use of this motif in the development of novel antiviral options. Similarly, derivatives (III^25^ and IV^28^) containing 1,2,3-triazoles exhibited strong inhibitory activity and good safety profiles. Additionally, the derivative V^29^ based on pyrazoles demonstrated exceptional inhibitory activity against M^pro^. All of these results point to isatin, 1,3,4-oxadiazole, 1,2,3-triazole, and pyrazole as preferred pharmacophores for the creation of new M^pro^ inhibitors. Furthermore, M^pro^ inhibitory activity can be considerably increased by substitutions at the C-5 and N1 positions of the isatin scaffold, particularly by inserting hydrophobic groups at N1, according to earlier structure–activity relationship (SAR) studies.

Accordingly, two series of *N*-substituted isatin hybrids were designed by integrating the isatin nucleus with a 1,3,4-oxadiazole-containing arm terminating in either a 1,2,3-triazole (9a–h) or a pyrazole moiety (15a–d). *N*1 of the isatin scaffold was selected as the point of attachment because substitution at this position permits structural extension without masking the two carbonyl groups of the isatin nucleus, thereby preserving their potential involvement in hydrogen-bonding interactions. A three-carbon propyl spacer was introduced to provide sufficient separation between the relatively rigid isatin nucleus and the terminal heterocyclic region, while imparting the conformational flexibility required for the hybrid molecules to accommodate the extended M^pro^ binding pocket. This spacer was also expected to reduce steric interference between the combined pharmacophores and allow them to explore different regions of the active site. The propyl chain was maintained throughout the present series to provide a consistent molecular framework for evaluating the effects of the terminal heterocycle and isatin substitution. Therefore, its use represents an initial design choice rather than evidence that three carbon atoms constitute the optimum linker length; systematic variation of the spacer length would be required to establish a linker-length SAR. Additionally, various substituents were introduced into the isatin nucleus to examine their effects on M^pro^ inhibitory activity and to establish preliminary SARs.

A FRET-based assay was first used to evaluate the synthesized agents against SARS-CoV-2 M^pro^ at a single concentration. To further understand their inhibitory mechanisms, the most active derivatives underwent enzyme kinetic studies, cytotoxicity assessment in normal IMR-90 cells, and IC_50_ determination. To obtain a deeper understanding of their binding modalities and interaction dynamics within the M^pro^ active site, molecular docking and molecular dynamics simulations were performed, along with *in silico* physicochemical and ADMET analysis.

## Results and discussion

### Chemistry

**Scheme 1:**
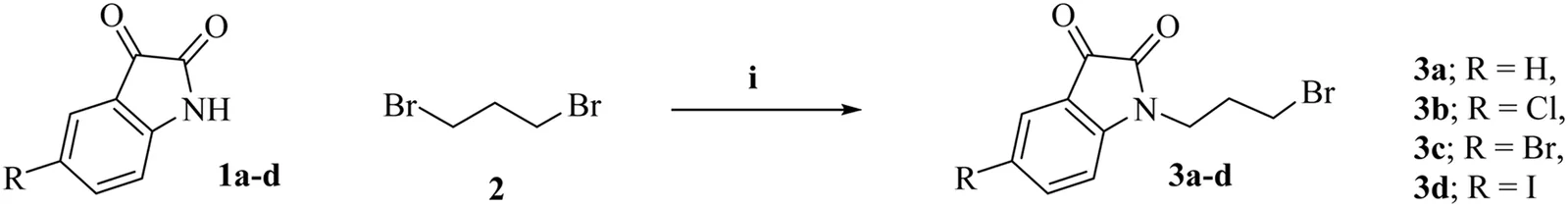
The synthesis of *N*-propyl bromo isatins as key intermediates (3a–d). Reagents and conditions: (i) K_2_CO_3_, DMF, stirred at rt for 2 h.

**Scheme 2:**
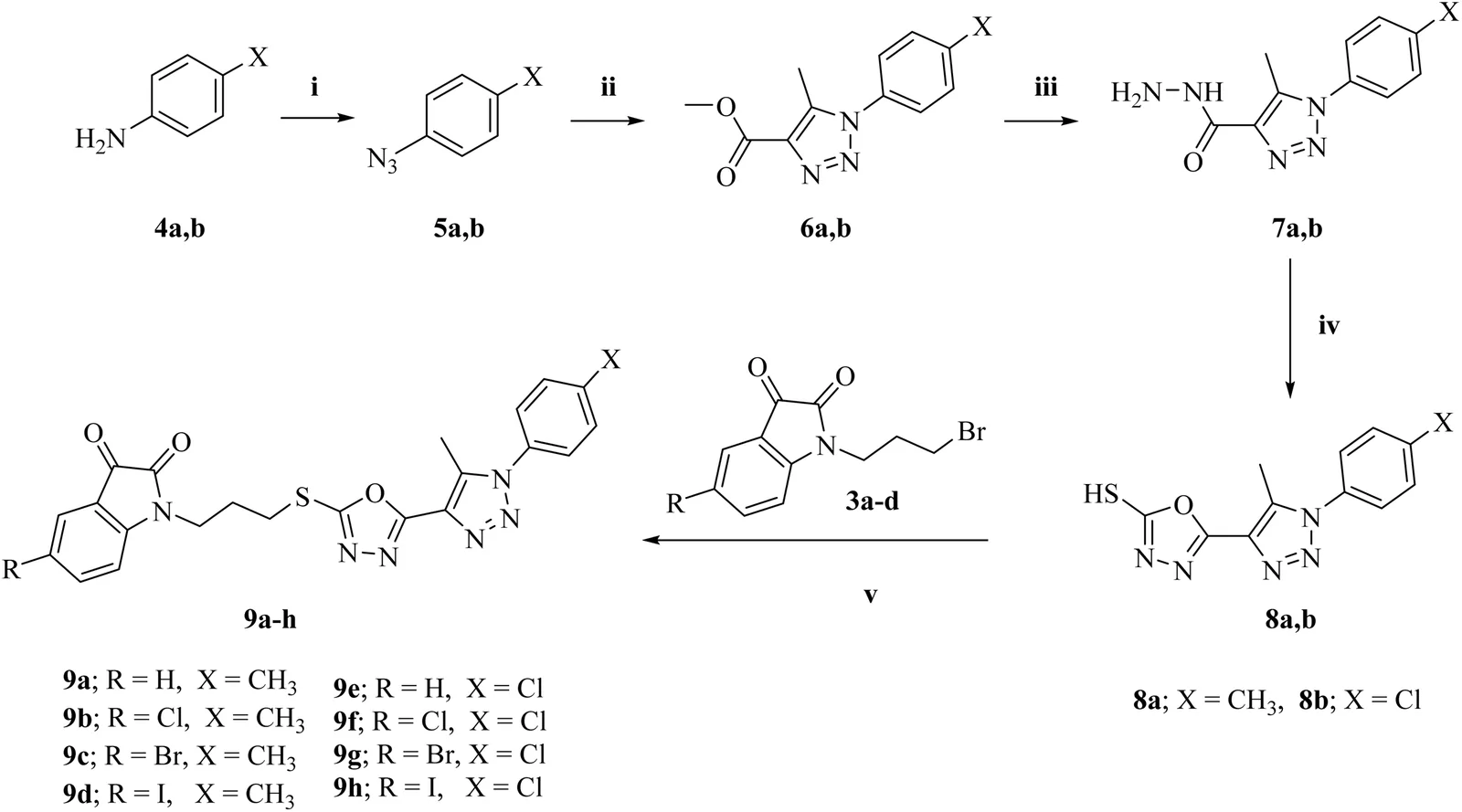
The synthesis of target compounds 1,2,3-triazole-linked 1,3,4-oxadiazole-tethered *N*-substituted isatin derivatives (9a–h). Reagents and conditions: (i) (a) NaNO_2_, HCl, 0 °C, (b) NaN_3_, 0 °C(ii) methyl acetoacetate, NaOCH_3_, CH_3_OH, rt (iii) CH_3_OH, NH_2_NH_2_ H_2_O, 6 h, reflux (iv) CS_2_, KOH, C_2_H_5_OH, 4 h, reflux (v) DMF, K_2_CO_3_, 24 h, rt.

**Scheme 3:**
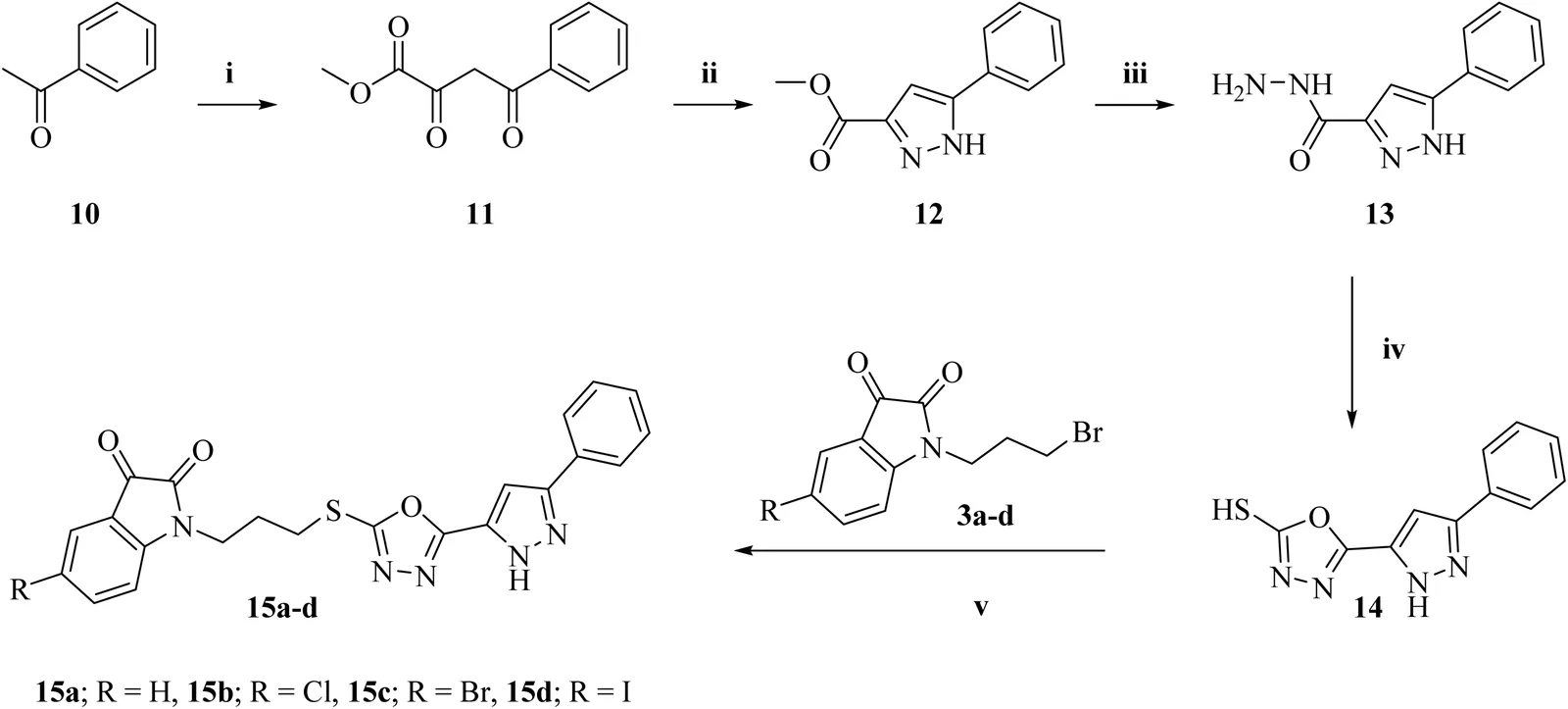
The synthesis of target compounds 1,2-diazole-linked 1,3,4-oxadiazole-tethered *N*-substituted isatin derivatives (15a–d). Reagents and conditions: (i) dimethyl oxalate, NaOCH_3_, THF, rt, 8 h; (ii) NH_2_NH_2_·H_2_O, acetic acid, reflux, 1 h; (iii) EtOH, NH_2_NH_2_ H_2_O, 6 h reflux; (iv) CS_2_, KOH, C_2_H_5_OH, 4 h reflux; (v) DMF, K_2_CO_3_, 24 h rt.

As shown in Schemes 1–3, a multistep synthetic method was used to synthesize the *N*-propyl bromoisatins (3a–d), critical intermediates, and the designed target hybrid molecules (9a–h and 15a–d) from various isatins. Scheme 1 illustrates how several isatins (1a–d) were first *N*-alkylated using 1,3-dibromopropane (2) in the presence of anhydrous potassium carbonate (K_2_CO_3_) in dry DMF at room temperature.^30^ The corresponding nucleophilic nitrogen species is generated in this reaction by K_2_CO_3_, which acts as a mild base to deprotonate the isatin NH. This species then undergoes S_N_2 displacement at one bromide terminal of 1,3-dibromopropane to yield the corresponding *N*-(3-bromopropyl)isatin derivatives (3a–d). DMF was chosen as a polar aprotic solvent to enhance the nucleophilicity of the isatin anion and accelerate the substitution process.

Concurrently, conc. hydrochloric acid (HCl) and sodium nitrite (NaNO_2_) were used to diazotize commercially available 4-methylaniline (4a) and 4-chloroaniline (4b) into their respective diazonium salts at a low temperature (0 °C).^31^ Mechanistically, sodium nitrite interacts with hydrochloric acid to generate nitrous acid *in situ*, which subsequently generates the electrophilic nitrosonium ion (NO^+^). The aromatic amines react with this species to generate unstable diazonium intermediates. Subsequent treatment with sodium azide resulted in nucleophilic substitution of the diazonium group, accompanied by the evolution of nitrogen gas, affording the corresponding aryl azides (5a,b). The produced azides (5a,b) subsequently underwent regioselective 1,3-dipolar cycloaddition with methyl acetoacetate in methanolic sodium methoxide solution to furnish the corresponding 1,2,3-triazole methyl esters (6a,b) in excellent yields (>80%). The reaction proceeds through cycloaddition between the azide 1,3-dipole and the activated methylene system of methyl acetoacetate under basic conditions, resulting in the synthesis of the substituted 1,2,3-triazole nucleus. Sodium methoxide not only forms the enolate intermediate from methyl acetoacetate but also facilitates ring closure and aromatization of the triazole scaffold. Thereafter, the ester derivatives (6a,b) were treated with excess hydrazine hydrate (99%) in methanol to give the corresponding carbohydrazides (7a,b) *via* nucleophilic acyl substitution. In this process, hydrazine attacks the ester carbonyl carbon, followed by elimination of methanol to form the hydrazide intermediates. These intermediates serve as flexible synthetic precursors for the production of heterocyclic rings. Cyclization of the hydrazides (7a,b) with carbon disulfide (CS_2_) in ethanolic KOH produced the corresponding 5-substituted-1,3,4-oxadiazole-2-thiol derivatives (8a,b).

Mechanistically, the equivalent dithiocarbazate intermediate is first produced *via* a basic reaction between the terminal hydrazide NH and carbon disulfide. Heating causes intramolecular cyclodehydration, releasing hydrogen sulfide and forming the thermodynamically stable 1,3,4-oxadiazole ring. The thiol form is sufficiently nucleophilic for additional alkylation reactions, and the isolated products are primarily found in the thiol-thione tautomeric equilibrium. The target 1,2,3-triazole-linked 1,3,4-oxadiazole-tethered *N*-substituted isatin hybrids (9a–h) were obtained by reacting the thiol compounds (8a,b) with *N*-(3-bromopropyl)isatin derivatives (3a–d) in DMF using K_2_CO_3_ as a base, as shown in Scheme 2. After the mercapto group is deprotonated, the equivalent thiolate anion is produced. This anion then undergoes S_N_2 substitution at the bromopropyl moiety, forming the required thioether bond that joins the two pharmacophoric fragments.

In Scheme 3, methyl 2,4-dioxo-4-phenylbutanoate (11),^32^ a β-diketoester intermediate, was obtained *via* Claisen-type condensation between acetophenone (10) and dimethyl oxalate in the presence of sodium methoxide. In this process, the acidic α-proton of acetophenone is abstracted by sodium methoxide, producing the corresponding enolate ion, which then attacks the electrophilic carbonyl carbon of dimethyl oxalate. The methoxide is then eliminated, producing the β-dicarbonyl system. The equivalent pyrazole methyl ester derivative was obtained by treating compound 11 with hydrazine hydrate in refluxing acetic acid (12).

The aromatic pyrazole nucleus is produced by cyclocondensation and dehydration after hydrazine's initial nucleophilic attack on the β-diketoester's carbonyl groups. By activating the carbonyl functionalities, acetic acid promotes condensation and cyclization. After being treated with hydrazine hydrate in methanol, the pyrazole ester derivative (12) underwent hydrazinolysis of the ester moiety, resulting in the equivalent carbohydrazide intermediate (13). Compound 13 underwent cyclization with carbon disulfide in ethanolic KOH, as in the previous pathway, to get the equivalent 5-(3-phenyl-1*H*-pyrazol-5-yl)-1,3,4-oxadiazole-2-thiol derivative (14) by forming a dithiocarbazate intermediate and then intramolecular ring closure.^33^ Lastly, the required pyrazole-linked 1,3,4-oxadiazole-tethered *N*-substituted isatin hybrids (15a–d) were obtained by *S*-alkylation of compound 14 with the previously synthesized *N*-(3-bromopropyl)isatin derivatives (3a–d) in DMF/K_2_CO_3_ through nucleophilic substitution by the produced thiolate species.

A variety of spectroscopic and analytical methods, including ^1^H NMR, ^13^C NMR, COSY, HMQC, and elemental analysis, confirmed the structures of the final compounds 9a–h and 15a–d. The elimination of the distinctive mercapto/thiol proton signal of intermediates 8a,b, and 14 and the emergence of novel aliphatic signals owing to the propyl linker joining the isatin nitrogen and the oxadiazole sulfur atom were the primary indicators of successful *S*-alkylation. The addition of the *N*-propyl thioether spacer was confirmed by the appearance of unique aliphatic resonances in the ^1^H NMR spectra of the newly inserted –NCH_2_–, central –CH_2_–, and –SCH_2_– protons. Specifically, thioether production *via* sulfur alkylation was supported by the downfield methylene signal attributed to –SCH_2_–.

By displaying three distinctive aliphatic carbon signals that corresponded to the propyl linker carbons, NCH_2_, CH_2_, and SCH_2_, the ^13^C NMR spectra further verified the target structures. Furthermore, the integrity of both pharmacophoric fragments following alkylation was corroborated by the preservation of diagnostic carbon signals of the isatin carbonyl groups and the heteroaromatic oxadiazole/triazole or pyrazole carbons. The successful synthesis of the end products was evidenced by the absence of unexpected signals from unreacted bromopropyl isatin or thiol intermediates.

By correlating each proton signal with its directly attached carbon resonance, HMQC analysis enabled the unambiguous assignment of protonated carbon atoms, particularly those corresponding to the three methylene groups of the propyl linker. This confirmed the connectivity of the newly introduced spacer between the oxadiazole and isatin moieties. Moreover, the ^1^H–^1^H COSY spectrum established the scalar coupling relationships among the adjacent methylene protons of the propyl chain, verifying the sequential proton connectivity and further supporting the proposed linkage between the two pharmacophoric fragments. The suggested molecular formulae and the purity of the synthesized compounds were further confirmed by elemental analysis, which showed good agreement (±0.4) between the calculated and observed values for carbon, hydrogen, nitrogen, and sulfur.

### Biological evaluations

SARS-CoV-2 M^pro^ was recombinantly expressed in *E. coli* BL21(DE3) and purified in-house by Ni-NTA affinity chromatography followed by size-exclusion chromatography, with purity confirmed by SDS-PAGE as described previously.^34^ This purified preparation was used for all enzymatic and kinetic experiments described in Table S2.

**Fig. 2:**
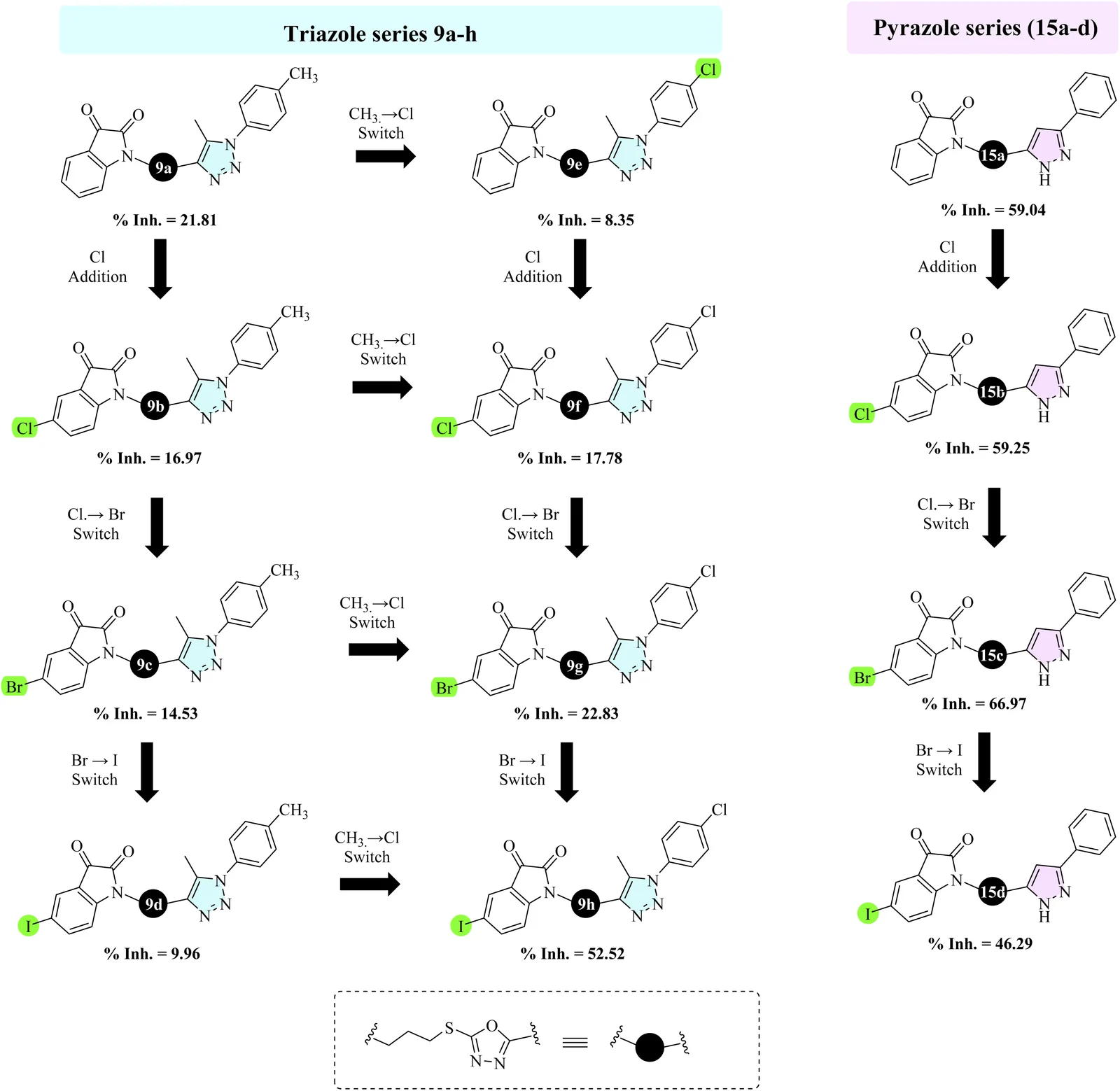
Structure–activity relationships of compounds 9a–h and 15a–d against SARS-CoV-2 M^pro^.

The synthetic 1,3,4-oxadiazole-tethered *N*-substituted isatin hybrids were first screened utilizing the FRET-based enzyme assay at a single concentration of 100 µM against the well-known SARS-CoV-2 target, M^pro^. These hybrids were divided into two series based on the azole ring that was incorporated: pyrazole-linked derivatives (15a–d) and 1,2,3-triazole-linked derivatives (9a–h). Remarkably, the target compounds exhibited a wide range of inhibitory effects against M^pro^, with inhibition percentages ranging from 8.35% to 66.97% (Table 1 and Fig. 2).

**Table 1:** Percentage inhibition of SARS-CoV-2 M^pro^ by the twelve 1,3,4-oxadiazole-linked *N*-substituted isatin derivatives and boceprevir at a concentration of 100 µM, as determined using the FRET-based assay

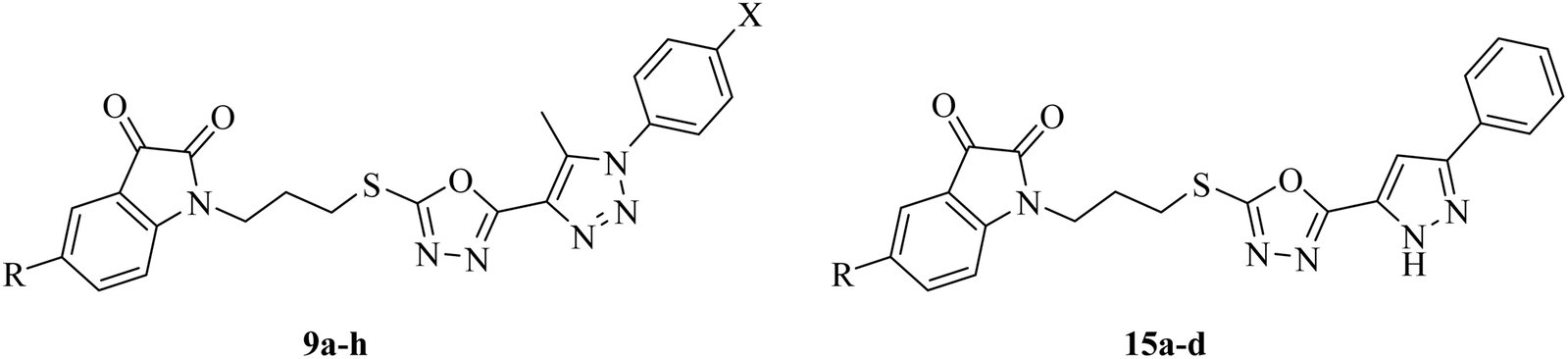			
Compounds			Inhibition%^a^
Code	Structure variants		
	R	X	
9a	H	CH_3_	21.81
9b	Cl	CH_3_	16.97
9c	Br	CH_3_	14.53
9d	I	CH_3_	9.96
9e	H	Cl	8.35
9f	Cl	Cl	17.78
9g	Br	Cl	22.83
9h	I	Cl	52.52
15a	H	—	59.04
15b	Cl	—	59.25
15c	Br	—	66.97
15d	I	—	46.29
Boceprevir			98.91

a All experiments were conducted at least in triplicate to ensure reproducibility.

SARS-CoV-2 M^pro^ inhibitory action clearly depends on both the terminal azole motif and the isatin nucleus substitution pattern, according to the preliminary SAR analysis. Compared with the majority of the triazole-linked derivatives 9a–h, the pyrazole-linked analogues 15a–d generally showed better inhibitory profiles, indicating that the phenylpyrazole motif is more advantageous for productive interaction with the M^pro^ binding pocket than the phenyl-1,2,3-triazole counterpart.

The unsubstituted isatin analogue 15a demonstrated a moderate inhibition of 59.04% at 100 µM within the pyrazole-linked series 15a–d. Since compound 15b inhibited M^pro^ by 59.25%, the introduction of a 5-chloro substituent showed almost identical activity, suggesting that chlorine substitution at C-5 has no effect in this subseries. It's interesting to note that substituting the bulkier, more polarizable bromine atom for chlorine significantly increased inhibitory activity; compound 15c exhibited the highest inhibition in the pyrazole series, at 66.97%. Improved hydrophobic and/or halogen-bonding interactions within the enzyme pocket could account for this enhancement. However, as seen with 15d, which showed only 46.29% inhibition, further expansion of the halogen substituent to iodine led to a marked decline in activity. This pattern implies that while the bulkier iodine may cause steric repulsion or interfere with the ideal binding orientation, the M^pro^ binding site may withstand bromine substitution at the isatin C-5 location.

The type of *para*-substituent on the phenyl ring connected to the triazole nucleus was crucial for the triazole-linked derivatives 9a–h. With the exception of the unsubstituted isatin pair 9a and 9e, the 4-chlorophenyl triazole analogues 9e–h generally exhibited greater inhibitory action than their corresponding 4-methylphenyl counterparts 9a–d. This result suggests that the more hydrophobic and electron-withdrawing chloro substituent on the terminal phenyl ring is often preferable to the electron-donating methyl group, especially when paired with halogenated isatin analogues.

The unsubstituted isatin derivative 9e exhibited only 8.35% inhibition in the 4-chlorophenyl triazole subseries 9e–h. Compound 9f demonstrated 17.78% inhibition, indicating that the addition of chlorine at the isatin C-5 site roughly doubled the activity. For compound 9g, further substitution with bromine raised the inhibitory efficacy to 22.83%. The 5-iodoisatin analogue 9h, which reached 52.52% inhibition, more than six times higher than the unsubstituted counterpart 9e, showed a notable improvement. This gradual improvement from H to Cl, Br, and I indicates that increasing halogen size and polarizability at the isatin C-5 position is advantageous in the 4-chlorophenyl triazole series, potentially due to improved halogen-mediated interactions and stronger hydrophobic contacts.

On the other hand, the 4-methylphenyl triazole subseries 9a–d exhibited the opposite trend. With 21.81% inhibition, the unsubstituted isatin derivative 9a displayed the greatest activity in this subseries. However, as was seen with 9b, 9c, and 9d, which showed 16.97%, 14.53%, and 9.96% inhibition, respectively, C-5 halogenation of the isatin ring caused a progressive loss of activity. This order of falling activity suggests that further halogen substitution of the isatin moiety is undesirable when the terminal phenyl ring bears an electron-donating methyl group. The combined steric and electronic effects of the methylphenyl-triazole fragment and the C-5-halogenated isatin nucleus may hamper the ideal alignment of the molecules within the M^pro^ active site.

When taken as a whole, our SAR results point to three key structural factors that influence SARS-CoV-2 M^pro^ inhibition. First, since compounds 15a–d normally perform better, substituting a pyrazole motif for the triazole linker significantly increases activity. Second, C-5 isatin halogenation shows a strongly scaffold-dependent effect: in the pyrazole series, bromine is ideal; in the 4-chlorophenyl triazole series, iodine is advantageous; and in the 4-methylphenyl triazole series, halogenation is detrimental. Third, especially when combined with halogenated isatin analogues, the 4-chlorophenyl group on the triazole ring is typically preferred over the 4-methylphenyl group. With the greatest M^pro^ inhibition of 66.97% at 100 µM, 15c was the most promising derivative among all tested agents. It was followed by 15b/15a and the iodinated 4-chlorophenyl triazole derivative 9h.

### Multiple-dose screening against SARS-CoV-2 M^pro^

**Fig. 3:**
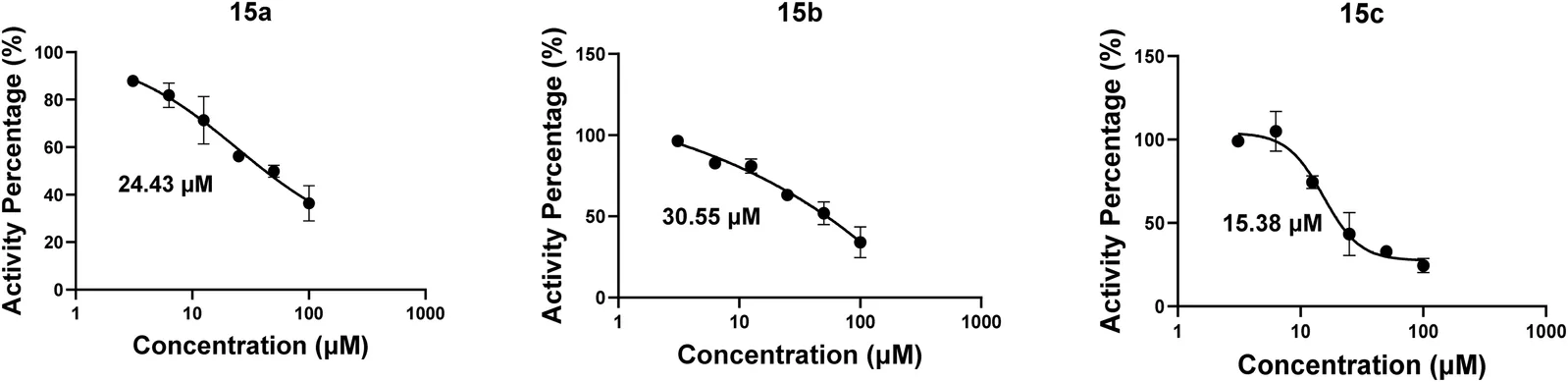
Concentration-dependent inhibition of SARS-CoV-2 M^pro^ by the most active isatin derivatives within the series (15a–c). The inhibitory activities of the tested compounds were evaluated by monitoring M^pro^ enzymatic activity over a range of inhibitor concentrations. IC_50_ values were determined by nonlinear regression analysis of the resulting dose–response curves.

The FRET assay was used to estimate the half-maximal inhibitory concentrations (IC_50_) of compounds that showed inhibition percentages greater than 55% in the main screening test (15a–c). These compounds were then moved on to a more thorough biological investigation. Boceprevir (IC_50_ = 8.78 µM) was used as the positive control when measuring enzymatic activity in the presence of increasing concentrations of the investigated agents (Table 2 and Fig. 3).

**Table 2:** Determination of IC_50_ values (µM) for the most potent derivatives (15a–c) and the reference inhibitor boceprevir

Compound	IC_50_ values a(µM)
15a	24.43
15b	30.55
15c	15.38
Boceprevir	8.78

a All experiments were conducted at least twice to ensure reproducibility.

With IC_50_ values ranging from 15.38 to 30.55 µM, the compounds examined exhibited encouraging inhibitory activity against SARS-CoV-2 M^pro^. Compounds 15a and 15b showed moderate inhibitory effects with IC_50_ values of 24.43 and 30.55 µM, respectively, while the pyrazole-linked derivative 15c showed the most inhibitory activity with an IC_50_ value of 15.38 µM. The substitution pattern of the pyrazole-linked scaffold plays a crucial role in regulating inhibitory activity against SARS-CoV-2 M^pro^, as evidenced by the greater potency of 15c. Molecular docking and molecular dynamics simulations were used to investigate this observation further. Notably, the main objective of this investigation was to identify potential M^pro^ inhibitors from the synthesized 1,3,4-oxadiazole-tethered *N*-substituted isatin hybrids. To fully evaluate the therapeutic potential of these interesting compounds, additional research is needed to assess selectivity against comparable human and viral proteases, as well as to conduct mechanistic and cellular antiviral assessments.

### Cytotoxicity assay

**Fig. 4:**
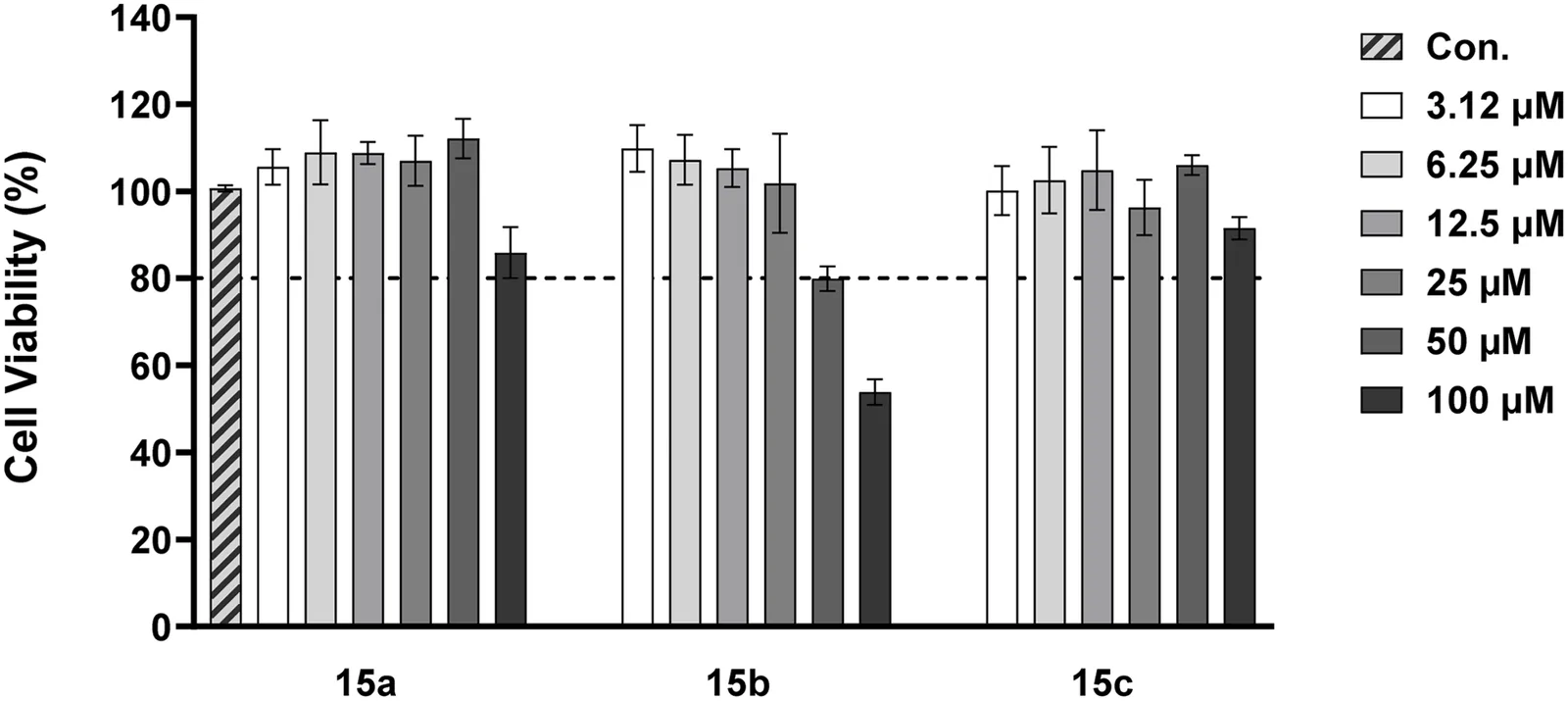
Assessment of the cytotoxicity of the three most active isatin derivatives within the series (15a, 15b, and 15c) toward IMR-90 cells.

An important step in lead optimization is assessing the safety profile of newly discovered inhibitors in normal human cells, taking into account the potential side effects of antiviral medicines, including several clinically licensed medications. The most active SARS-CoV-2 M^pro^ inhibitors within the present series, 15a, 15b, and 15c, were therefore evaluated for cytotoxicity using the normal human lung fibroblast cell line IMR-90. None of the examined agents showed significant cytotoxic effects over the analyzed concentration range, as shown in Fig. 4, demonstrating their high tolerance toward normal cells and favorable safety profiles. Notably, their observed inhibitory activity against SARS-CoV-2 M^pro^, together with their favorable cytotoxicity profiles, supports their consideration as preliminary hits for further investigation and structural optimization. Further pharmacological and antiviral research is necessary, as the observed selectivity for the viral target over normal human cells may offer a favorable treatment window.

### M^pro^ kinetics analysis

**Fig. 5:**
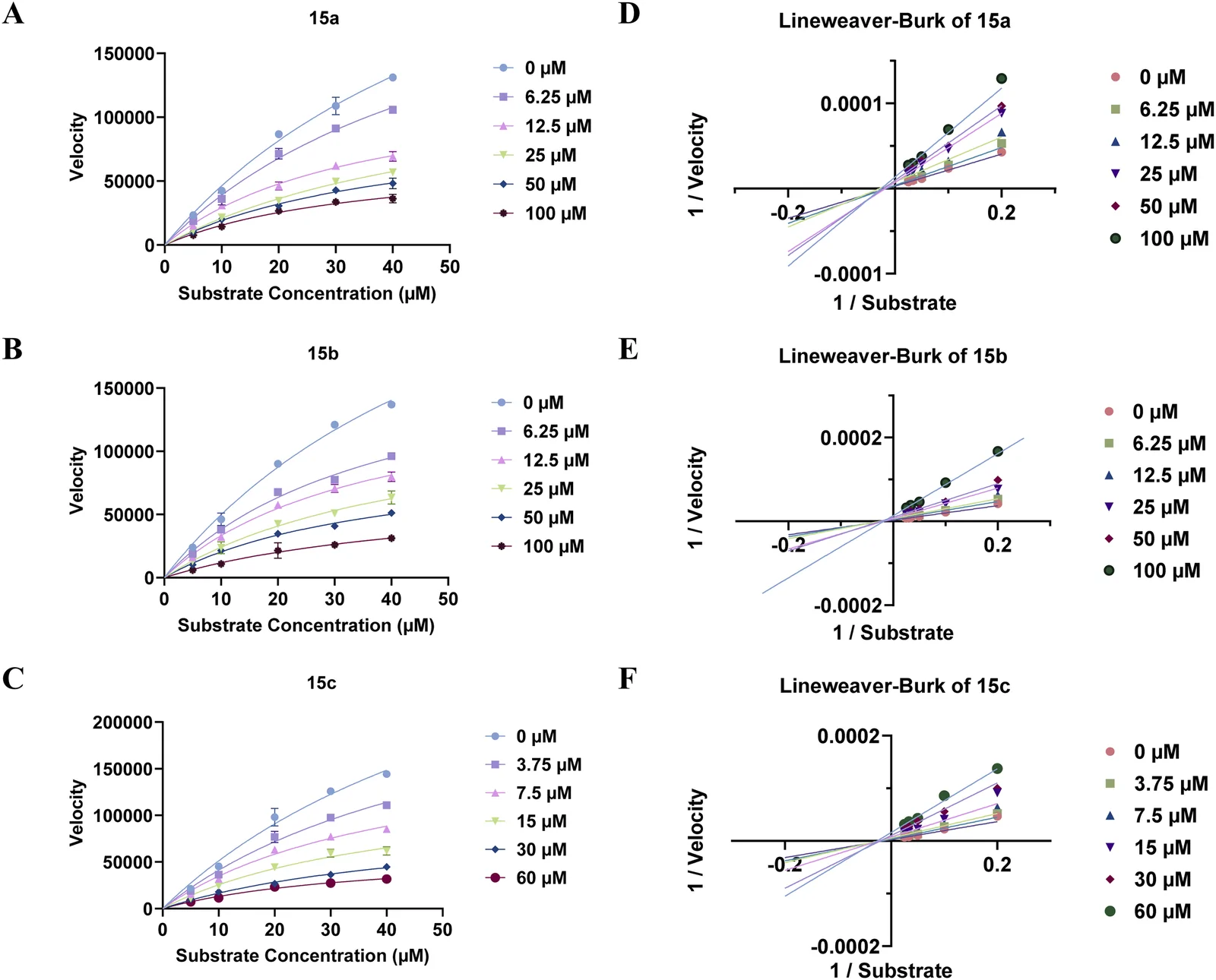
Enzyme kinetic analysis of SARS-CoV-2 M^pro^ in the presence of the most active isatin derivatives within the series. Initial reaction rates were measured over a range of substrate concentrations at different inhibitor levels and fitted using the Michaelis–Menten equation by nonlinear regression, as detailed in the Experimental section. Michaelis–Menten curves are presented in panels (A–C), while the corresponding Lineweaver–Burk plots are shown in panels (D–F). The analyzed compounds are: 15a (A and D), 15b (B and E), and 15c (C and F).

Enzyme kinetic experiments were carried out for compounds 15a, 15b, and 15c at various inhibitor concentrations and across varying substrate concentrations to gain a deeper understanding of the inhibitory mechanism of the most active compounds against SARS-CoV-2 M^pro^. Fig. 5 shows the Michaelis–Menten generated and double-reciprocal Lineweaver–Burk plots, while Table 3 summarizes the computed kinetic parameters.

**Table 3:** Kinetic parameters derived from global nonlinear fitting of Michaelis–Menten kinetics for the most active isatin derivatives within the series against SARS-CoV-2 M^pro^

Compound	Inhibition constant, *K*_I_ (µM)	Alpha (*α*)	*R* ^2^ value	Mechanism of inhibition
15a	24.98	0.41	0.95	Noncompetitive/mixed
15b	34.55	0.44	0.97	Noncompetitive/mixed
15c	13.24	0.41	0.98	Mixed/noncompetitive

As demonstrated by the concentration-dependent drop in reaction velocity seen in the Michaelis–Menten plots, the kinetic profiles showed that increasing concentrations of the investigated agents gradually decreased the catalytic effectiveness of M^pro^. Although the corresponding Lineweaver–Burk plots showed patterns consistent with mixed-type inhibition, the untransformed initial-velocity data were further evaluated by global nonlinear fitting with competitive, noncompetitive, uncompetitive, and mixed-type inhibition models. AICc-based model comparison showed that the noncompetitive and mixed models provided comparable fits for all three compounds (ΔAICc < 2), whereas the competitive and uncompetitive models were less supported. Residual analysis showed no pronounced systematic dependence on substrate concentration or fitted velocity for the preferred models (Table S3). Accordingly, the kinetic profiles are more conservatively described as noncompetitive/mixed inhibition.

Compounds 15a, 15b, and 15c were found to have inhibition constants (*K*_I_) of 24.98, 34.55, and 13.24 µM, respectively. This indicates that compound 15c is the most effective kinetic inhibitor, consistent with its superior IC_50_ value observed in the enzymatic inhibition assay. The *α* values of the three compounds ranged from 0.41 to 0.44, with the corresponding confidence intervals encompassing unity. Within the mixed-model framework, these *α* estimates are consistent with a possible preference for binding to the enzyme–substrate complex, while the model-comparison analysis indicated comparable support for mixed and noncompetitive inhibition.

Taken together, these findings indicate that the 1,3,4-oxadiazole-tethered *N*-substituted isatin hybrids inhibit SARS-CoV-2 M^pro^ through a predominantly noncompetitive/mixed kinetic profile. The *α* values further suggest a possible tendency toward enzyme–substrate complex formation within the mixed-inhibition model, although this feature is interpreted in light of the comparable support for the noncompetitive model. Overall, the kinetic results support further investigation of these compounds, particularly 15c, as a preliminary hit for the development of SARS-CoV-2 M^pro^ inhibitors (Table 3).

### *In silico* studies

#### Predicted ADMET and physicochemical properties

**Fig. 6:**
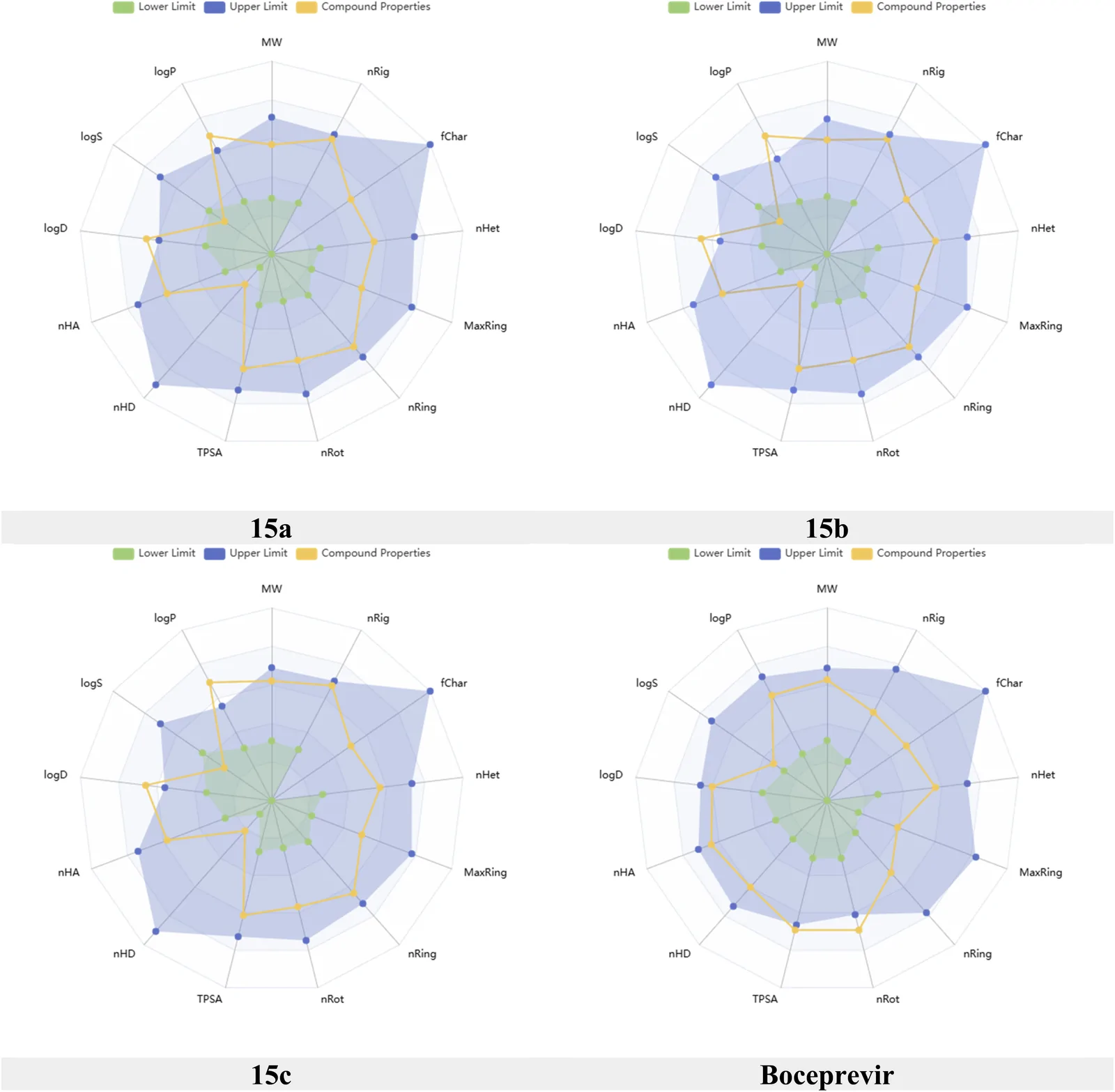
Radar plots illustrate the predicted physicochemical properties of compounds 15a, 15b, 15c, and boceprevir, generated by ADMETlab 3.0. The orange line represents the compound properties, while the green and blue areas indicate the lower and upper acceptable limits, respectively.

The assessment of pharmacokinetic and physicochemical properties is essential in the early phases of drug discovery to identify prospective lead compounds with desirable drug-like features.^35–42^ Thus, ADMETlab 3.0 was used to predict the physicochemical profiles of the reference inhibitor boceprevir and the most active SARS-CoV-2 M^pro^ inhibitors, 15a, 15b, and 15c. The resulting radar plots showed that each substance under investigation had balanced physicochemical characteristics, with most descriptors falling within the suggested limits (Fig. 6).

Interestingly, compound 15c, the most active derivative within the present series, displayed a physicochemical profile comparable to that of boceprevir, suggesting potentially favorable drug-like characteristics. Collectively, these findings identify the synthesized 1,3,4-oxadiazole-tethered *N*-substituted isatin hybrids as preliminary hit structures that may provide a useful basis for further structural optimization toward more potent SARS-CoV-2 M^pro^ inhibitors.

#### Docking analysis

Because compound 15c exhibited a favorable inhibition profile, it was selected for detailed molecular docking and molecular dynamics (MD) simulations. Enzyme assays indicated that 15c operates *via* a non-covalent mechanism; thus, non-covalent docking simulations were performed using AutoDock Vina to explore its potential binding mode within the SARS-CoV-2 main protease (M^pro^).

**Fig. 7:**
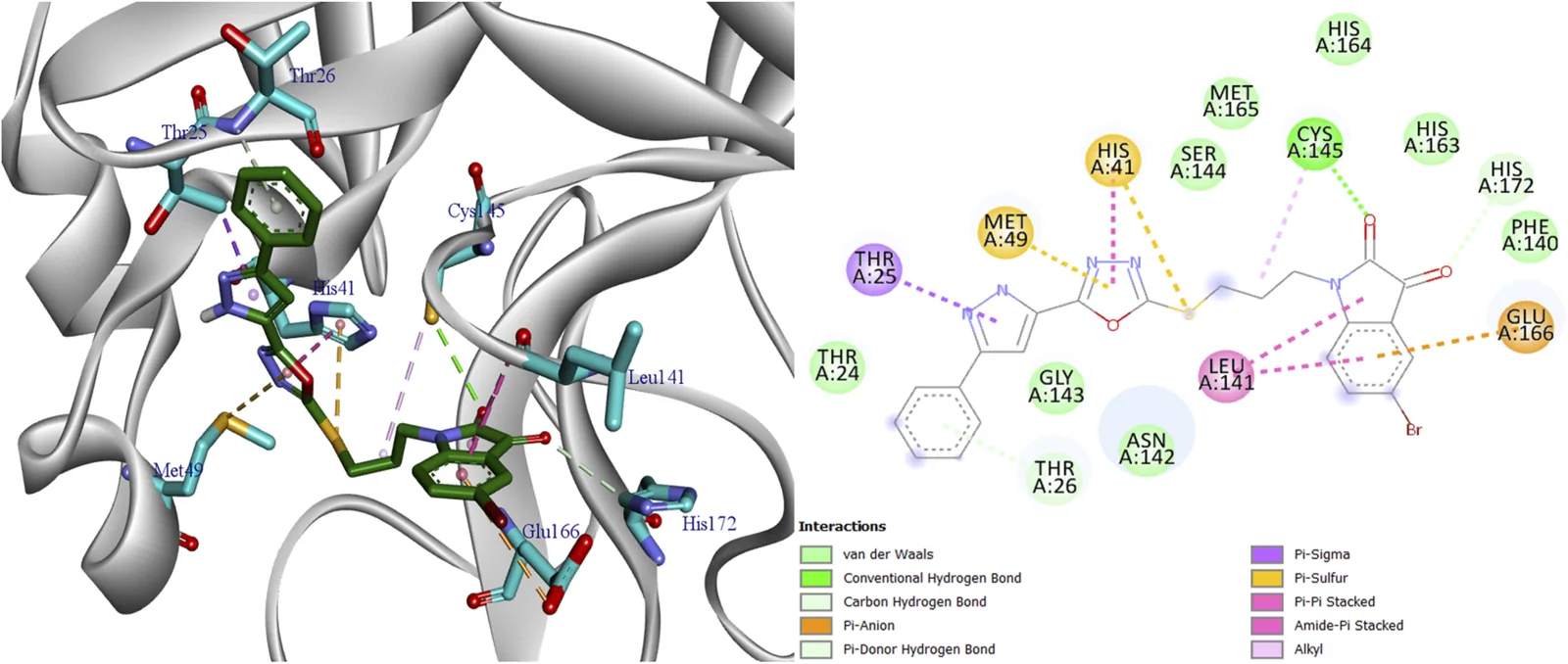
Presentation of the best docking pose through active site view (left) and 2D interaction plot (right) for 15c inside SARS-CoV-2 M^pro^ protein.

Docking 15c into the allosteric site yielded a weak binding score of −6.0 kcal mol^−1^ (compared to −6.1 kcal mol^−1^ for the reference compound). Conversely, docking into the active site demonstrated a strong binding affinity of −8.3 kcal mol^−1^, whereas the reference compound scored −9.5 kcal mol^−1^. These data suggest that compound 15c fits favorably within the active site of SARS-CoV-2 M^pro^. As illustrated in Fig. 7, 15c establishes several key interactions with crucial active-site residues, including hydrogen bonding and hydrophobic interactions. It forms interactions with the key residues Thr25, His41, Cys145, and Glu166. In addition, it engages in hydrophobic contacts with Thr26, His41, Met49, Pro52, and Met165 (Fig. 7). Furthermore, docking of 9c and 9g, the lowest-active derivatives, showed a similar pattern of interactions with lower affinity than that of 15c (Fig. S44). This can be attributed to the benzene ring (yellow-highlighted) interaction with the Thr24 (NH) group. The presence of methyl or chlorine increased electron density on the ring, which disfavored interaction with the NH. In addition, the triazole derivatives showed lower activity than diazole for the same reason.

Together, these molecular docking results reinforce the potential of compound 15c as a SARS-CoV-2 M^pro^ inhibitor and foster further *in silico* validation *via* molecular dynamics simulations.

To assess whether the observed structure–activity trends are supported across the full activity range, representative derivatives from the potent and weakly active groups were compared. The most active compound 15c (66.97% inhibition at 100 µM; IC_50_ 15.38 µM, *K*_I_ 13.24 µM) was contrasted with the weakly active derivatives 9g (22.83%) and 9c (14.53%). Docking of 9c and 9g reproduced the overall binding orientation adopted by 15c within the active site, but with lower predicted affinity and fewer stabilizing contacts, consistent with the experimental rank order. The combined steric and electronic effects of the *para*-substituted terminal aryl ring in the triazole-linked series appear less favorable for engagement of the active-site residues than the corresponding region of the pyrazole-linked scaffold, consistent with the generally lower activity of series 9a–h relative to 15a–d. The experimental and computational analyses, therefore, converge on the same ordering across the series, although the limited number of congeners means these trends should be regarded as preliminary and require confirmation with an expanded set of analogs.

#### Molecular dynamics

In this study, extended computational investigations were performed using MD simulations to enable a detailed assessment of binding affinity within the ligand–target complex. The binding poses predicted by docking compound 15c with SARS-CoV-2 M^pro^ were further analyzed using MD simulations. To provide a reference for evaluating the influence of each ligand on enzyme stability, additional simulations were conducted on the Apo (ligand-free) form of the protease.

**Fig. 8:**
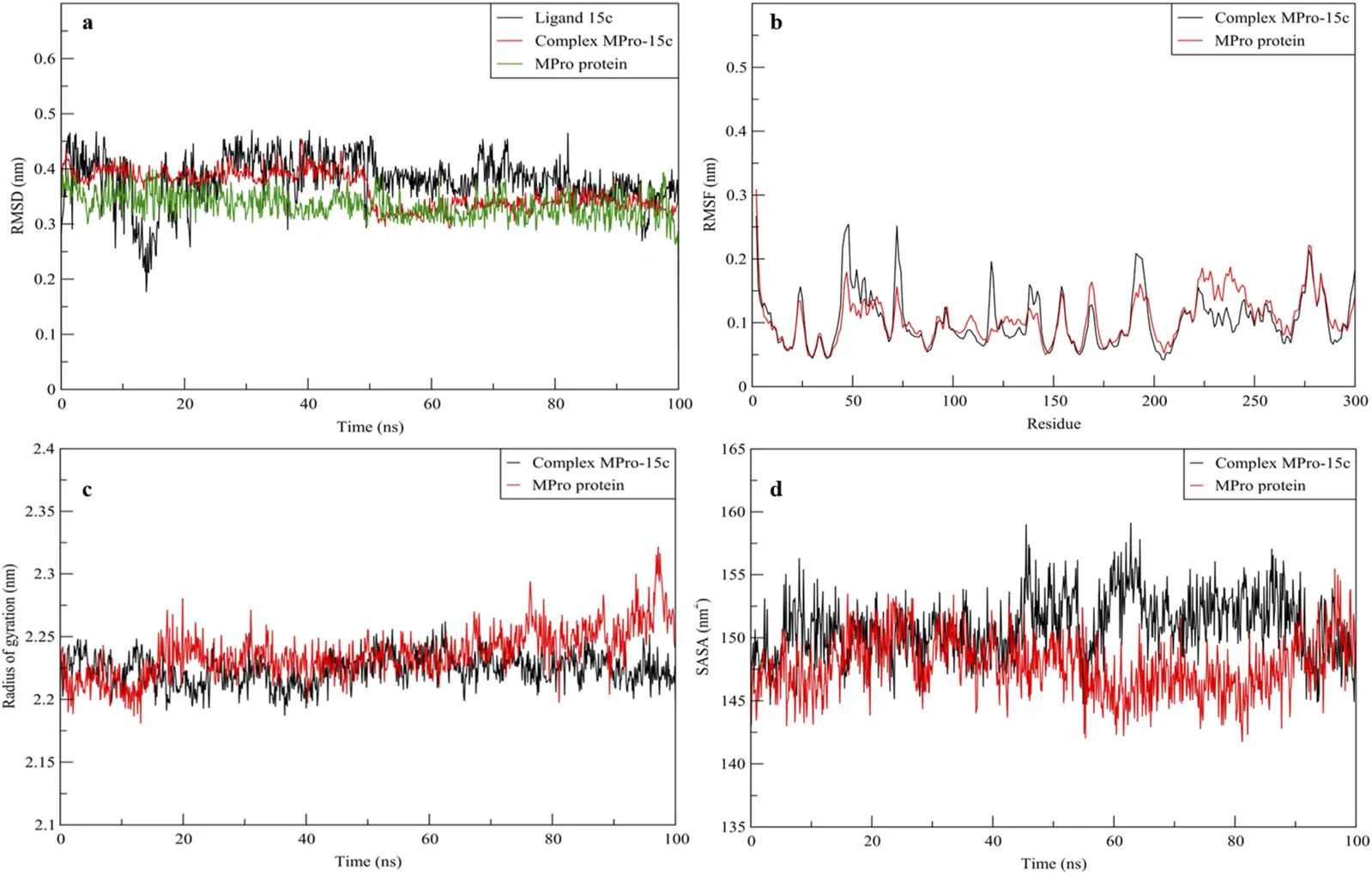
Analysis plot of (a) RMSD, (b) RMSF, (c) radius of gyration, and (d) SASA for 15c complexed and Apo forms of SARS-CoV-2 M^pro^.

As illustrated in Fig. 8, compound 15c preserved the structural stability of SARS-CoV-2 M^pro^, as indicated by RMSD values comparable to those of the Apo form, ranging between 0.35 and 0.45 nm. Moreover, ligand 15c retained its initial binding position throughout the simulation, suggesting the formation of a stable complex. RMSF analysis further revealed similar fluctuation profiles for both the Apo and ligand-bound systems. The original conformation of SARS-CoV-2 M^pro^ remained intact upon binding with 15c, with no significant structural deviations observed in the radius of gyration or SASA analyses.

**Fig. 9:**
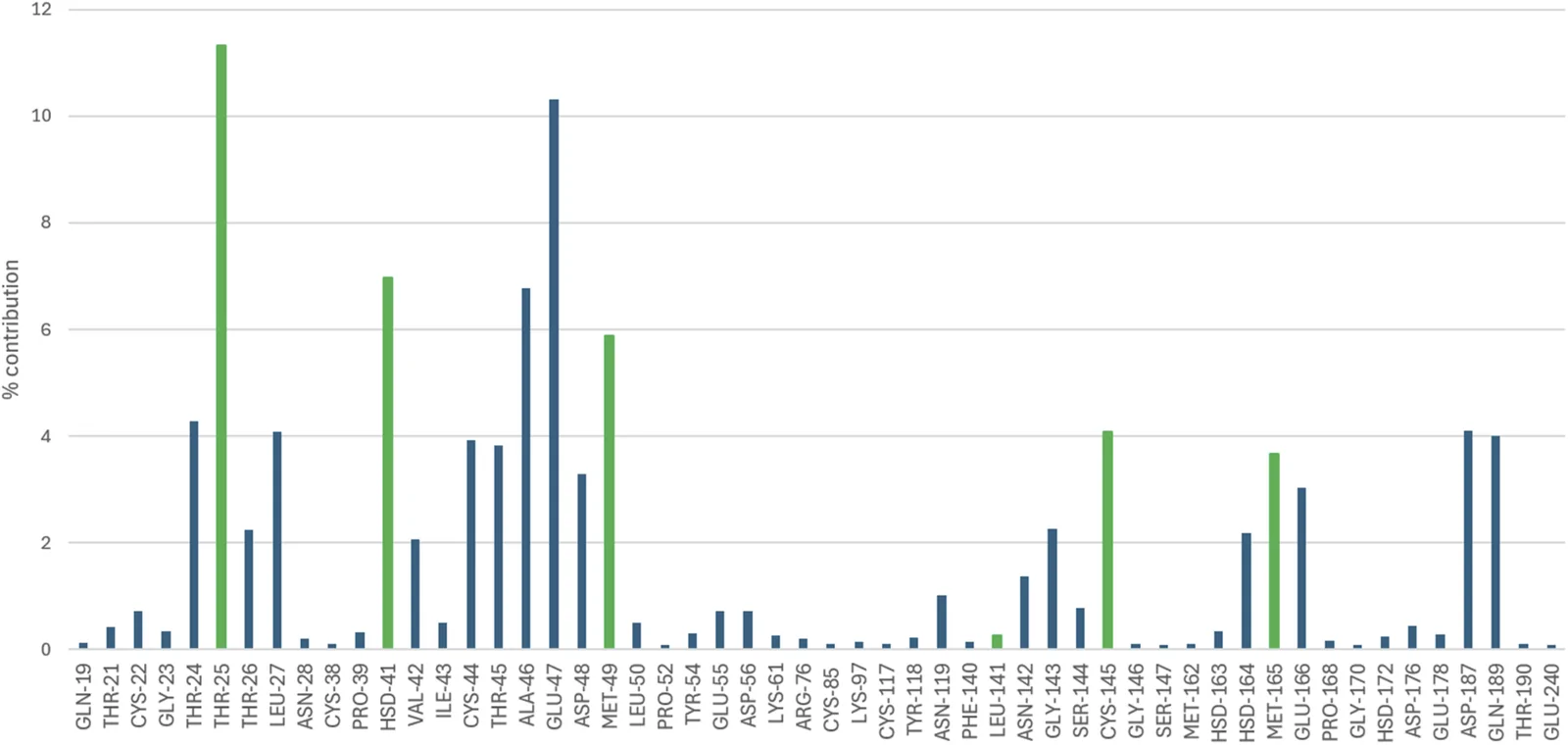
Plot of the various interacting amino acids with compound 15c into the SARS-CoV-2 M^pro^ active cavity.

To further support the MD findings, the g_mmpbsa package was employed to estimate the binding free energy of compound 15c in complex with SARS-CoV-2 M^pro^. Trajectories obtained from the production phase were analyzed to evaluate key energy components, including electrostatic interactions, van der Waals forces, polar solvation energy, and SASA contributions. The results revealed that compound 15c exhibits a favorable binding free energy (Table 4). Additionally, residue-level energy decomposition indicated that key amino acids in the active site significantly contribute to the stabilization of 15c, as shown in Fig. 9. Overall, these binding free-energy calculations strengthen the reliability of the molecular modeling approach and offer deeper insight into the ligand-protein interactions in this system.

**Table 4:** Free binding energies of 15c with SARS-CoV-2 M^pro^ protein in kJ mol^−1^

Binding energies (kJ mol^−1^)	Electrostatic energy	Polar solvation energy	SASA energy	van der Waals energy	Δ*G*
15c	−27.31 ± 18.34	110.53 ± 38.19	−15.572 ± 2.25	−132.29 ± 25.23	−64.646 ± 1.54

## Conclusion

The current study synthesized and assessed two series of 1,3,4-oxadiazole-tethered *N*-substituted isatin hybrids, namely 1,2,3-triazole-linked derivatives (9a–h) and pyrazole-linked derivatives (15a–d), as possible inhibitors of SARS-CoV-2 main protease (M^pro^). Compounds 15a–c were identified as the most active derivatives in the initial screening, with M^pro^ inhibition percentages exceeding 55%. With an IC_50_ of 15.38 µM and a *K*_I_ of 13.24 µM, 15c was the most effective inhibitor. Compounds 15a, 15b, and 15c inhibit M^pro^*via* a noncompetitive/mixed mechanism, according to mechanistic experiments. Additionally, these compounds exhibited low cytotoxicity toward normal IMR-90 cells under the tested conditions, indicating a favorable preliminary cytotoxicity profile. Molecular docking and molecular dynamics simulations supported a stable predicted binding mode of the most active compound, 15c, within the M^pro^ binding pocket and provided a plausible molecular explanation for its comparatively greater inhibitory activity within the present series. *In silico* physicochemical and ADMET analyses also suggested potentially favorable drug-like properties. Collectively, these findings identify 15c as a preliminary hit rather than an optimized lead. Further structural optimization, particularly systematic investigation of the linker length, together with comprehensive antiviral and mechanistic studies, will be required to establish the therapeutic potential of this scaffold.

## Experimental

### Chemistry

#### General

All commercially available reagents and solvents were obtained from suppliers and used without further purification, unless otherwise specified. Reaction progress and product purity sheets of silica gel aluminum with a fluorescent indicator at 254 nm and 0.2 mm thickness were monitored by thin-layer chromatography (TLC) with FLUK. The melting points were estimated using the SMP10 melting point apparatus (Stuart, Japan) and are shown uncorrected. The ^1^H and ^13^C NMR spectra in DMSO-*d*_6_ were recorded on a Bruker spectrometer (Japan) operating at 600 and 126 MHz, respectively. Elemental microanalyses were carried out using a PerkinElmer 2400 analyzer (PerkinElmer, USA). During the evaluation process, samples were burned in an oxygen-rich atmosphere, yielding CO_2_, N_2_, H_2_O, and nitrogen oxides. Following their equal mixing under atmospheric pressure, the percentages of carbon, hydrogen, and nitrogen in the resulting gaseous products were measured using a thermal conductivity detector.

#### General procedure and analytical data for 1,3,4-oxadiazole-tethered *N*-substituted isatin hybrids (9a–h and 15a–d)

The appropriate *N*-(3-bromopropyl)isatin derivatives (3a–d, 1 mmol) were reacted with the corresponding 5-substituted-1,3,4-oxadiazole-2-thiol derivatives (8a,b) or 5-(3-phenyl-1*H*-pyrazol-5-yl)-1,3,4-oxadiazole-2-thiol (14, 1 mmol) in dry DMF (5 mL) in the presence of anhydrous K_2_CO_3_ (1.5 mmol). The reaction mixture was stirred at room temperature for 24 h, and the progress of the reaction was monitored by TLC using *n*-hexane/ethyl acetate (1 : 2, v/v) as the eluent. Upon completion, the reaction mixture was poured onto crushed ice and neutralized with acetic acid. The resulting precipitate was collected by filtration, washed thoroughly with diethyl ether, and dried to afford the corresponding target compounds.

##### 1-(3-((5-(5-Methyl-1-(*p*-tolyl)-1*H*-1,2,3-triazol-4-yl)-1,3,4-oxadiazol-2-yl)thio)propyl)indoline-2,3-dione (9a)

Yellow solid (83%); M.p. 140 °C; ^1^H NMR (600 MHz, DMSO-*d*_6_) *δ*: 7.66 (t, *J* = 7.8 Hz, 1H, Ar–H), 7.56 (t, *J* = 8.3 Hz, 3H, Ar–Hs), 7.48 (d, *J* = 7.8 Hz, 2H, Ar–Hs), 7.24 (d, *J* = 7.9 Hz, 1H, Ar–H), 7.13 (t, *J* = 7.5 Hz, 1H, Ar–H), 3.85 (t, *J* = 6.7 Hz, 2H, NCH_2_), 3.41 (t, *J* = 7.3 Hz, 2H, SCH_2_), 2.57 (s, 3H, triazole-CH_3_), 2.44 (s, 3H, phenyl-CH_3_), 2.16 (p, *J* = 7.0 Hz, 2H, CH_2_**CH̲**_**2**_CH_2_). ^13^C NMR (151 MHz, DMSO-*d*_6_) *δ*: 183.75 (ketonic C=O), 163.77, 159.87, 158.90, 150.94, 140.58, 138.48, 136.63, 133.09, 131.03, 130.67, 125.59, 124.90, 123.60, 118.19, 111.02, 38.64 (NCH_2_), 30.08 (SCH_2_), 27.49 (**C̲H**_**2**_CH_2_CH_2_), 21.24 (phenyl-CH_3_), 10.05 (triazole-CH_3_). MS (ESI): *m*/*z*: [M]^+^ calcd. 460.13 and found 460.12. Anal. Calcd. for C_23_H_20_N_6_O_3_S: C, 59.99; H, 4.38; N, 18.25; S, 6.96; found C, 60.24; H, 4.39; N, 18.28; S, 6.99%.

##### 5-Chloro-1-(3-((5-(5-methyl-1-(*p*-tolyl)-1*H*-1,2,3-triazol-4-yl)-1,3,4-oxadiazol-2-yl)thio)propyl)indoline-2,3-dione (9b)

Yellow solid (86%); M.p. 184 °C; ^1^H NMR (600 MHz, DMSO-*d*_6_) *δ*: 7.70 (d, *J* = 8.4 Hz, 1H, Ar–H), 7.61 (s, 1H, Ar–H), 7.57 (d, *J* = 7.8 Hz, 2H, Ar–Hs), 7.48 (d, *J* = 7.9 Hz, 2H, Ar–Hs), 7.28 (d, *J* = 8.5 Hz, 1H, Ar–Hs), 3.85 (t, *J* = 6.6 Hz, 2H, NCH_2_), 3.40 (t, *J* = 7.2 Hz, 2H, SCH_2_), 2.57 (s, 3H, triazole-CH_3_), 2.44 (s, 3H, phenyl-CH_3_), 2.15 (t, *J* = 7.1 Hz, 2H, CH_2_**CH̲**_**2**_CH_2_). ^13^C NMR (151 MHz, DMSO-*d*_6_) *δ*: 182.66 (ketonic C=O), 163.73, 159.87, 158.70, 149.45, 140.58, 137.28, 136.63, 133.09, 131.02, 130.67, 127.80, 125.59, 124.38, 119.64, 112.75, 38.73 (NCH_2_), 30.00 (SCH_2_), 27.38 (CH_2_**C̲H**_**2**_CH_2_), 21.24 (phenyl-CH_3_), 10.05 (triazole-CH_3_). MS (ESI): *m*/*z*: [M]^+^ calcd. 494.09 and found 494.07. Anal. Calcd. for C_23_H_19_ClN_6_O_3_S: C, 55.81; H, 3.87; N, 16.98; S, 6.48; found C, 56.00; H, 3.85; N, 17.04; S, 6.51%.

##### 5-Bromo-1-(3-((5-(5-methyl-1-(*p*-tolyl)-1*H*-1,2,3-triazol-4-yl)-1,3,4-oxadiazol-2-yl)thio)propyl)indoline-2,3-dione (9c)

Yellow solid (79%); M.p. 178 °C; ^1^H NMR (600 MHz, DMSO-*d*_6_) *δ*: 7.90–7.76 (m, 1H, Ar–Hs), 7.71 (s, 1H, Ar–H), 7.57 (s, 2H, Ar–Hs), 7.48 (s, 2H, Ar–Hs), 7.29–7.17 (m, 1H, Ar–H), 3.85 (s, 2H, NCH_2_), 3.40 (s, 2H, SCH_2_), 2.57 (s, 3H, triazole-CH_3_), 2.44 (s, 3H, phenyl-CH_3_), 2.14 (s, 2H, CH_2_**CH̲**_**2**_CH). ^13^C NMR (151 MHz, DMSO-*d*_6_) *δ*: 182.51 (ketonic C=O), 163.73, 159.86, 158.53, 149.82, 140.58, 140.10, 136.63, 133.09, 131.02, 130.67, 127.10, 125.58, 120.00, 115.32, 113.19, 38.72 (NCH_2_), 30.00 (SCH_2_), 27.36 (CH_2_**C̲H**_**2**_CH_2_), 21.24 (phenyl-CH_3_), 10.05 (triazole-CH_3_). MS (ESI): *m*/*z*: [M]^+^ calcd. 538.04 and found 538.03. Anal. Calcd. for C_23_H_19_BrN_6_O_3_S: C, 51.21; H, 3.55; N, 15.58; S, 5.94; found C, 50.99; H, 3.57; N, 15.60; S, 5.94%.

##### 5-Iodo-1-(3-((5-(5-methyl-1-(*p*-tolyl)-1*H*-1,2,3-triazol-4-yl)-1,3,4-oxadiazol-2-yl)thio)propyl)indoline-2,3-dione (9d)

Yellow solid (66%); M.p. 148 °C; ^1^H NMR (600 MHz, DMSO-*d*_6_) *δ*: 7.97 (dd, *J* = 8.3, 1.9 Hz, 1H, Ar–H), 7.81 (d, *J* = 1.9 Hz, 1H, Ar–H), 7.57 (d, *J* = 8.3 Hz, 2H, Ar–Hs), 7.48 (d, *J* = 8.1 Hz, 2H, Ar–Hs), 7.11 (d, *J* = 8.3 Hz, 1H, Ar–Hs), 3.83 (t, *J* = 6.6 Hz, 2H, NCH_2_), 3.40 (t, *J* = 7.2 Hz, 2H, SCH_2_), 2.57 (s, 3H, triazole-CH_3_), 2.45 (s, 3H, phenyl-CH_3_), 2.14 (p, *J* = 6.8 Hz, 2H, CH_2_**CH̲**_**2**_CH_2_). ^13^C NMR (151 MHz, DMSO-*d*_6_) *δ*: 182.43 (ketonic C=O), 163.73, 159.86, 158.26, 150.26, 145.88, 140.59, 136.64, 133.09, 132.54, 131.03, 130.68, 125.60, 120.28, 113.52, 86.47, 38.67 (NCH_2_), 30.00 (SCH_2_), 27.35 (CH_2_**CH̲**_**2**_CH_2_), 21.25 (phenyl-CH_3_), 10.06 (triazole-CH_3_). MS (ESI): *m*/*z*: [M]^+^ calcd. 586.02 and found 586.03. Anal. Calcd. for C_23_H_19_IN_6_O_3_S: C, 47.11; H, 3.27; N, 14.33; S, 5.47; found C, 46.95; H, 3.27; N, 14.29; S, 5.48%.

##### 1-(3-((5-(1-(4-Chlorophenyl)-5-methyl-1*H*-1,2,3-triazol-4-yl)-1,3,4-oxadiazol-2-yl)thio)propyl)indoline-2,3-dione (9e)

Yellow solid (81%); M.p. 162 °C; ^1^H NMR (600 MHz, DMSO-*d*_6_) *δ*: 7.66 (t, *J* = 7.8 Hz, 1H, Ar–H), 7.56 (t, *J* = 8.3 Hz, 3H, Ar–Hs), 7.48 (d, *J* = 7.9 Hz, 2H, Ar–Hs), 7.24 (d, *J* = 7.9 Hz, 1H, Ar–H), 7.13 (t, *J* = 7.5 Hz, 1H, Ar–H), 3.85 (t, *J* = 6.7 Hz, 2H, Ar–Hs), 3.41 (t, *J* = 7.3 Hz, 2H, SCH_2_), 2.44 (s, 3H, triazole-CH_3_), 2.16 (p, *J* = 6.8 Hz, 2H, CH_2_**CH̲**_**2**_CH_2_). MS (ESI): *m*/*z*: [M]^+^ calcd. 480.07 and found 480.08. Anal. Calcd. for C_22_H_17_ClN_6_O_3_S: C, 54.94; H, 3.56; N, 17.48; S, 6.67; found C, 55.17; H, 3.55; N, 17.44; S, 6.69%.

##### 5-Chloro-1-(3-((5-(1-(4-chlorophenyl)-5-methyl-1*H*-1,2,3-triazol-4-yl)-1,3,4-oxadiazol-2-yl)thio)propyl)indoline-2,3-dione (9f)

Yellow solid (86%); M.p. 188 °C; ^1^H NMR (600 MHz, DMSO-*d*_6_) *δ*: 8.63 (t, *J* = 5.7 Hz, 1H, Ar–H), 7.76 (d, *J* = 2.2 Hz, 3H, Ar–Hs), 7.55 (d, *J* = 2.6 Hz, 1H, Ar–H), 7.32 (dd, *J* = 9.0, 2.7 Hz, 1H, Ar–H), 6.81 (d, *J* = 9.1 Hz, 1H, Ar–H), 3.42–3.38 (dt, *J* = 10.3, 6.7 Hz, 4H, NCH_2_ and SCH_2_), 2.60 (s, 3H, triazole-CH_3_), 2.11 (t, *J* = 7.0 Hz, 2H, CH_2_**CH̲**_**2**_CH_2_). ^13^C NMR (151 MHz, DMSO-*d*_6_) *δ*: 200.70 (ketonic C=O), 169.09, 163.88, 159.74, 149.95, 136.94, 135.35, 134.38, 134.13, 133.84, 131.26, 130.32, 127.67, 127.61, 117.60, 115.83, 113.60, 40.98 (NCH_2_), 30.41 (SCH_2_), 28.91 (CH_2_**C̲H**_**2**_CH_2_), 10.04 (triazole-CH_3_). MS (ESI): *m*/*z*: [M]^+^ calcd. 514.03 and found 514.04. Anal. Calcd. for C_22_H_16_Cl_2_N_6_O_3_S: C, 51.27; H, 3.13; N, 16.31; S, 6.22; found C, 50.99; H, 3.15; N, 16.29; S, 6.23%.

##### 5-Bromo-1-(3-((5-(1-(4-chlorophenyl)-5-methyl-1*H*-1,2,3-triazol-4-yl)-1,3,4-oxadiazol-2-yl)thio)propyl)indoline-2,3-dione (9g)

Yellow solid (77%); M.p. 202 °C; ^1^H NMR (600 MHz, DMSO-*d*_6_) *δ*: 7.83 (d, *J* = 8.4 Hz, 1H, Ar–H), 7.76 (s, 4H, Ar–Hs), 7.71 (s, 1H, Ar–H), 7.23 (d, *J* = 9.2 Hz, 1H, Ar–H), 3.85 (d, *J* = 7.6 Hz, 2H, NCH_2_), 3.40 (s, 2H, SCH_2_), 2.60 (s, 3H, triazole-CH_3_), 2.14 (s, 2H, CH_2_**CH̲**_**2**_CH_2_). ^13^C NMR (151 MHz, DMSO-*d*_6_) *δ*: 182.51 (ketonic C=O), 163.84, 159.74, 158.54, 149.82, 140.09, 136.94, 135.39, 134.36, 131.22, 130.35, 127.64, 127.11, 120.02, 115.32, 113.19, 38.71 (NCH_2_), 30.00 (SCH_2_), 27.36 (CH_2_**C̲H**_**2**_CH_2_), 10.05 (triazole-CH_3_). MS (ESI): *m*/*z*: [M]^+^ calcd. 557.98 and found 557.99. Anal. Calcd. for C_22_H_16_BrClN_6_O_3_S: C, 47.20; H, 2.88; N, 15.01; S, 5.73; found C, 46.93; H, 2.87; N, 14.99; S, 5.75%.

##### 5-Iodo-1-(3-((5-(1-(4-chlorophenyl)-5-methyl-1*H*-1,2,3-triazol-4-yl)-1,3,4-oxadiazol-2-yl)thio)propyl)indoline-2,3-dione (9h)

Yellow solid (73%); M.p. 204 °C; ^1^H NMR (600 MHz, DMSO-*d*_6_) *δ*: 7.97 (dd, *J* = 8.3, 1.9 Hz, 1H, Ar–Hs), 7.81 (d, *J* = 1.9 Hz, 1H, Ar–H), 7.76 (s, 4H, Ar–Hs), 7.11 (d, *J* = 8.3 Hz, 1H, Ar–Hs), 3.84 (t, *J* = 6.6 Hz, 2H, NCH_2_), 3.40 (t, *J* = 7.2 Hz, 2H, SCH_2_), 2.60 (s, 3H, triazole-CH_3_), 2.14 (p, *J* = 6.8 Hz, 2H, CH_2_**CH̲**_**2**_CH_2_). ^13^C NMR (151 MHz, DMSO-*d*_6_) *δ*: 182.43 (ketonic C=O), 163.84, 159.73, 158.26, 150.25, 145.88, 136.93, 135.39, 134.36, 132.55, 131.22, 130.35, 127.64, 120.28, 113.52, 86.48, 38.67 (NCH_2_), 30.00 (SCH_2_), 27.34 (CH_2_**C̲H**_**2**_CH_2_), 10.06 (triazole-CH_3_). MS (ESI): *m*/*z*: [M]^+^ calcd. 605.97 and found 605.95. Anal. Calcd. for C_22_H_16_ClIN_6_O_3_S: C, 43.55; H, 2.66; N, 13.85; S, 5.28; found C, 43.37; H, 2.66; N, 13.89; S, 5.25%.

##### 1-(3-((5-(3-Phenyl-1*H*-pyrazol-5-yl)-1,3,4-oxadiazol-2-yl)thio)propyl)indoline-2,3-dione (15a)

Yellow solid (65%); M.p. 132 °C; ^1^H NMR (600 MHz, DMSO-*d*_6_) *δ*: 14.84 (s, NH, 1H), 7.63 (t, *J* = 7.7 Hz, 1H, Ar–H), 7.55 (d, *J* = 7.2 Hz, 1H, Ar–H), 7.47 (q, *J* = 2.6 Hz, 3H, Ar–Hs), 7.32–7.30 (m, 3H, Ar–Hs), 7.23 (d, *J* = 7.9 Hz, 1H, Ar–H), 7.11 (t, *J* = 7.5 Hz, 1H, Ar–H), 3.85 (t, *J* = 6.4 Hz, 2H, NCH_2_), 3.41 (t, *J* = 7.2 Hz, 2H, SCH_2_), 2.15 (p, *J* = 6.8 Hz, 2H, CH_2_**CH̲**_**2**_CH_2_). ^13^C NMR (151 MHz, DMSO-*d*_6_) *δ*: 183.76 (ketonic C=O), 164.09, 161.09, 158.90, 150.94, 145.36, 139.52, 138.45, 137.51, 129.79, 129.18, 126.12, 124.89, 123.57, 118.20, 111.00, 107.97, 38.64 (NCH_2_), 30.02 (SCH_2_), 27.47 (CH_2_**C̲H**_**2**_CH_2_). MS (ESI): *m*/*z*: [M]^+^ calcd. 431.10 and found 431.12. Anal. Calcd. for C_22_H_17_N_5_O_3_S: C, 61.24; H, 3.97; N, 16.23; S, 7.43; found C, 61.06; H, 3.99; N, 16.19; S, 7.48%.

##### 5-Chloro-1-(3-((5-(3-phenyl-1*H*-pyrazol-5-yl)-1,3,4-oxadiazol-2-yl)thio)propyl)indoline-2,3-dione (15b)

Yellow solid (70%); M.p. 186 °C; ^1^H NMR (600 MHz, DMSO-*d*_6_) *δ*: 14.83 (s, NH, 1H), 7.68 (dd, *J* = 8.4, 2.3 Hz, 1H, Ar–H), 7.60 (d, *J* = 2.3 Hz, 1H, Ar–H), 7.48 (q, *J* = 2.7 Hz, 3H, Ar–Hs), 7.31 (d, *J* = 7.9 Hz, 3H, Ar–Hs), 7.26 (s, 1H, Ar–H), 3.85 (t, *J* = 6.6 Hz, 2H, NCH_2_), 3.40 (t, *J* = 7.2 Hz, 2H, SCH_2_), 2.14 (p, *J* = 6.8 Hz, 2H, CH_2_**CH̲**_**2**_CH_2_). ^13^C NMR (151 MHz, DMSO-*d*_6_) *δ*: 182.66 (ketonic C=O), 164.05, 161.09, 158.70, 149.46, 145.37, 139.52, 137.51, 137.24, 129.80, 129.18, 127.79, 126.11, 124.37, 119.64, 112.72, 107.97, 38.73 (NCH_2_), 29.95 (SCH_2_), 27.34 (CH_2_**C̲H**_**2**_CH_2_). MS (ESI): *m*/*z*: [M]^+^ calcd. 465.06 and found 465.05. Anal. Calcd. for C_22_H_16_ClN_5_O_3_S: C, 56.72; H, 3.46; N, 15.03; S, 6.88; found C, 56.91; H, 3.45; N, 14.98; S, 6.90%.

##### 5-Bromo-1-(3-((5-(3-phenyl-1*H*-pyrazol-5-yl)-1,3,4-oxadiazol-2-yl)thio)propyl)indoline-2,3-dione (15c)

Yellow solid (75%); M.p. 190 °C; ^1^H NMR (600 MHz, DMSO-*d*_6_) *δ*: 14.83 (s, NH, 1H), 7.75 (d, *J* = 59.5 Hz, 2H, Ar–Hs), 7.41–7.21 (m, 7H, Ar–Hs), 3.84 (s, 2H, NCH_2_), 3.40 (s, 2H, SCH_2_), 2.14 (s, 2H, CH_2_**CH̲**_**2**_CH_2_). ^13^C NMR (151 MHz, DMSO-*d*_6_) *δ*: 182.51 (ketonic C=O), 164.05, 161.09, 158.54, 149.83, 145.37, 140.05, 139.52, 137.51, 129.80, 129.16, 127.10, 126.11, 120.02, 115.31, 113.16, 107.97, 38.71 (NCH_2_), 29.94 (SCH_2_), 27.32 (CH_2_**C̲H**_**2**_CH_2_). MS (ESI): *m*/*z*: [M]^+^ calcd. 509.01 and found 509.00. Anal. Calcd. for C_22_H_16_BrN_5_O_3_S: C, 51.78; H, 3.16; N, 13.72; S, 6.28; found C, 52.00; H, 3.17; N, 13.75; S, 6.31%.

##### 5-Iodo-1-(3-((5-(3-phenyl-1*H*-pyrazol-5-yl)-1,3,4-oxadiazol-2-yl)thio)propyl)indoline-2,3-dione (15d)

Yellow solid (73%); M.p. 164 °C; ^1^H NMR (600 MHz, DMSO-*d*_6_) *δ*: 14.83 (s, NH, 1H), 7.94 (dd, *J* = 8.3, 1.9 Hz, 1H, Ar–H), 7.80 (d, *J* = 1.9 Hz, 1H, Ar–H), 7.49–7.47 (m, 3H, Ar–Hs), 7.33–7.30 (m, 3H, Ar–Hs), 7.09 (d, *J* = 8.3 Hz, 1H, Ar–H), 3.83 (t, *J* = 6.6 Hz, 2H, NCH_2_), 3.39 (t, *J* = 7.2 Hz, 2H, SCH_2_), 2.13 (p, *J* = 6.8 Hz, 2H, CH_2_**CH̲**_**2**_CH_2_). ^13^C NMR (151 MHz, DMSO-*d*_6_ with C_2_H_5_OD) *δ*: 182.43 (ketonic C=O), 164.04, 161.08, 158.26, 150.26, 145.83, 145.37, 139.52, 137.50, 132.54, 129.80, 129.18, 126.12, 120.29, 113.49, 107.97, 86.46, 38.66 (NCH_2_), 29.94 (SCH_2_), 27.30 (CH_2_**C̲H**_**2**_CH_2_). MS (ESI): *m*/*z*: [M]^+^ calcd. 557.00 and found 556.99. Anal. Calcd. for C_22_H_16_IN_5_O_3_S: C, 47.41; H, 2.89; N, 12.57; S, 5.75; found C, 47.23; H, 2.90; N, 12.62; S, 5.77%.

## Biological evaluations

SARS-CoV-2 M^pro^ assay

### Cloning, protein expression, and purification

SARS-CoV-2 M^pro^ from the Wuhan-Hu-1 isolate was expressed and purified as previously described.^43^ Briefly, the M^pro^ coding sequence was cloned into the pET21a(+) vector and transformed into *E. coli* BL21(DE3) cells. Protein expression was induced with 0.5 mM IPTG at an OD_600_ of 0.4–0.5, followed by incubation for 3 h at 37 °C. Cells were harvested, lysed using BugBuster Master Mix, and the soluble fraction was purified by Ni-NTA affinity chromatography. The eluted protein was concentrated and further purified by size-exclusion chromatography using a HiLoad™ 16/60 Superdex™ 200 pg column. Protein concentration was determined using the BCA assay, and SDS-PAGE was used to assess purity.

### Enzyme inhibition assays and IC_50_ determination

The SARS-CoV-2 M^pro^ FRET substrate DABCYL-KTSAVLQ↓SGFRKME-EDANS was purchased from BPS Bioscience (San Diego, CA, USA). SARS-CoV-2 M^pro^ inhibition was determined using a fluorogenic FRET assay based on our previously reported method,^43^ in which enzymatic cleavage of DABCYL-KTSAVLQ↓SGFRKME-EDANS separates the quencher and fluorophore, thereby increasing fluorescence.^3,44^ Briefly, reactions were performed in 100 µL containing 370 nM M^pro^, 20 µM substrate, 20 mM Tris–HCl buffer (pH 7.4), and test compounds initially screened at 100 µM. Reactions were initiated by the addition of substrate, and fluorescence was recorded every 60 s for 30 min at 25 °C using a Synergy HT microplate reader, with excitation and emission wavelengths of 360 and 460 nm, respectively. Reactions without inhibitor served as negative controls, whereas previously validated M^pro^ inhibitors were used as positive controls. For IC_50_ determination, active compounds were tested by two-fold serial dilution from 100 µM, and IC_50_ values were calculated by fitting initial velocities against inhibitor concentrations using GraphPad Prism version 10.1.1 (GraphPad Software, Inc., La Jolla, CA, USA).

### Cytotoxicity assay

IMR-90 cells (CCL-186™) were obtained from the American Type Culture Collection (ATCC, Manassas, VA, USA). Cytotoxicity was evaluated using the EZ-Cytox assay kit, as previously described,^26^ with minor modifications. Briefly, IMR-90 cells were cultured in DMEM supplemented with GlutaMAX™, 10% FBS, and 100 U mL^−1^ penicillin-streptomycin at 37 °C in a humidified 5% CO_2_ incubator. Cells were seeded in 96-well plates at 5 × 10^5^ cells per well and incubated until approximately 85% confluence. The cells were then treated with serial concentrations (100–3.12 µM) of the selected oxadiazole-isatin hybrids for 24 h, with DMSO-treated cells serving as the control. Subsequently, 10 µL of EZ-Cytox reagent was added to each well, followed by incubation for 1 h at 37 °C. Absorbance was measured at 450 nm using a Synergy HT microplate reader, and cell viability was calculated relative to untreated control cells.

### Michaelis–Menten kinetics assay

To determine the mode of inhibition of selected oxadiazole-isatin hybrids, enzyme kinetic assays were performed at a fixed SARS-CoV-2 M^pro^ concentration of 370 nM using substrate concentrations of 10, 20, 30, and 40 µM. Compounds 15a and 15b were tested using twofold serial dilutions starting at 100 µM, whereas compound 15c was tested using twofold serial dilutions starting at 60 µM; inhibitor-free reactions were included as controls. Initial velocities were calculated from the linear phase of substrate hydrolysis and analyzed using Michaelis–Menten kinetics. Lineweaver–Burk plots were generated for visualization. The untransformed initial-velocity data were globally fitted in GraphPad Prism 10.3.1 using competitive, noncompetitive, uncompetitive, and mixed-inhibition models. Model selection was assessed using the corrected Akaike information criterion (AICc), and ΔAICc values were used to compare the relative support for competing models. Residuals were examined for systematic dependence on substrate concentration and fitted velocity was used to assess model adequacy. For the mixed-inhibition model, the fitted *K*_i_ and *α* values were additionally evaluated to characterize inhibitor binding, while the overall inhibition mode was assigned based on the combined model-comparison and residual analyses.

### Molecular docking and dynamic simulation

The crystal structures of SARS-CoV-2 main protease (M^pro^) (PDB IDs: 8ACD^18^ and 7AXM^45^), containing inhibitors bound to the active and allosteric sites, respectively, were obtained in PDB format from the RCSB Protein Data Bank. Before docking, water molecules and other non-protein components were removed using AutoDockTools,^46^ and hydrogen atoms were subsequently added. The two- and three-dimensional structures of compound 15c were constructed using Marvin Sketch. Molecular docking studies were carried out using AutoDock Vina^47^ to predict binding affinities and characterize protein-ligand interactions. The grid box for docking was defined based on the coordinates of the co-crystallized ligands: (*x*, *y*, *z*; 9.6, 0.4, 21.7) for the active site and (7.3, −1.8, −19.7) for the allosteric site, with dimensions of 17 × 17 × 23 Å and 23 × 24 × 16 Å, respectively. The docking results were analyzed and visualized using Discovery Studio Visualizer. To further assess the stability of compound 15c within the SARS-CoV-2 M^pro^ binding site, molecular dynamics (MD) simulations were performed using the optimal docking pose. The CHARMM force field parameters were applied, and the system setup was conducted using the CHARMM-GUI Solution Builder.^48,49^ Ligand topologies were generated *via* the CGenFF server based on the CHARMM General Force Field.

The CHARMM-GUI workflow consists of five main steps. Initially, the protein-ligand complex coordinates are uploaded. Subsequently, the system is solvated, and its size and composition are defined, including the addition of Na^+^ and Cl^−^ ions to neutralize the system. Periodic boundary conditions (PBCs) are then applied to simulate an infinite system by repeating a unit cell in all directions, confining the simulation to atoms within the PBC box. In this study, the PBC box dimensions were set to 77 × 77 × 77 Å^3^, with Na^+^ ions added to ensure charge neutrality.^50^

Short energy minimization was performed to remove unfavorable contacts. This was followed by equilibration in two stages: the constant-volume and temperature (NVT) and constant-pressure and temperature (NPT) ensembles, ensuring the system reached stable thermodynamic conditions. Simulation parameters were adjusted after generating the input files, including the number of MD steps, trajectory output frequency, and energy calculations. The Verlet cutoff scheme was employed for neighbor searching, with a 12 Å cutoff for non-bonded interactions, while long-range electrostatic interactions were treated using the Particle Mesh Ewald (PME) method. Energy minimization was performed using the steepest-descent algorithm for 5000 steps.

The system was equilibrated for 125 ps at 300.15 K using positional restraints of 400 kJ·mol^−1^·nm^−2^ for backbone atoms and 40 kJ·mol^−1^·nm^−2^ for side chains under both NVT and NPT ensembles. Following equilibration, a 100 ns production simulation was performed at 310.15 K and 1 bar under NPT conditions. Temperature and pressure were maintained using the Nose–Hoover thermostat and Parrinello-Rahman barostat, respectively. Bond constraints involving hydrogen atoms were handled using the LINCS algorithm. Additionally, the V-rescale thermostat with a coupling constant of 1 ps was applied, and system trajectories were recorded every 2 ps. All simulations were performed using GROMACS 2020.2.^51^ The resulting trajectories were analyzed using GROMACS utilities, while Visual Molecular Dynamics (VMD) software^52^ was used for visualization, contact frequency analysis, and hydrogen-bonding evaluation. Binding free energy calculations were conducted using the MM/PBSA approach implemented in the g_mmpbsa package to estimate ligand-protein binding affinity.^53^

## Conflicts of interest

There are no conflicts to declare.

## Supplementary Material

RA-016-D6RA06004H-s001

## Data Availability

All data generated or analyzed during this study are included in this manuscript and its supplementary information (SI). Supplementary information is available. See DOI: https://doi.org/10.1039/d6ra06004h.
