# Supplementary material for: Design and synthesis of 1,3,4-oxadiazole-tethered N-substituted isatin hybrids as SARS-CoV-2 main protease inhibitors: biological evaluation, kinetic investigation, and in silico studies
Source: RSC Adv. 2026 Sep 1;16(42):51489–506. doi: 10.1039/d6ra06004h (PMC13531525; doi:10.1039/d6ra06004h)
Supplement: Supplementary file 1 [file RA-016-D6RA06004H-s001.pdf]

This journal is © The Royal Society of Chemistry 2026

Supporting Information

Design and Synthesis of 1,3,4-Oxadiazole-Tethered *N*-Substituted Isatin Hybrids as SARS-CoV-2 Main Protease Inhibitors: Biological Evaluation, Kinetic Investigation, and *In Silico* Studies

Tarfah Al-Warhi[1#], Md Sofequl Islam Mukim[2,3#], Zainab M Elsayed[4], Yasser M. Omar[5,6], Diaaeldin M. Elimam[7], Moataz A. Shaldam[8,9], Ashraf K. El-Damasy[10], Mostafa M. Elbadawi[8], Haytham O. Tawfik[11,9,*], Dae-Geun Song[2,3,*], Wagdy M. Eldehna[8,*]

[1] *Department of Chemistry, College of Science, Princess Nourah Bint Abdulrahman University, P.O. Box 84428, Riyadh 11671, Saudi Arabia*

[2] *Center for Natural Product Systems Biology, KIST Gangneung Institute of Natural Products, Gangneung 25451, Republic of Korea*

[3] *Natural Product Applied Science, KIST School, University of Science and Technology, Gangneung 25451, Republic of Korea*

[4] *Scientific Research and Innovation Support Unit, Faculty of Pharmacy, Kafrelsheikh University, Kafr El-Shaikh 33516, Egypt*

[5] *Institute of Cancer Therapeutics, University of Bradford, BD7 1DP, UK.*

[6] *Department of Pharmaceutical Organic Chemistry, Faculty of Pharmacy, Assiut University, Assiut 71526, Egypt.*

[7] *Department of Pharmacognosy, Faculty of Pharmacy, Kafrelsheikh University, Kafrelsheikh 33516, Egypt.*

[8] *Department of Pharmaceutical Chemistry, Faculty of Pharmacy, Kafrelsheikh University, Kafrelsheikh, P.O. Box 33516, Egypt*

[9] *Department of Pharmaceutical Chemistry, Faculty of Pharmacy, Alsalam University, 31511, Tanta, Egypt*

[10] *Department of Medicinal Chemistry, Faculty of Pharmacy, Mansoura University, Mansoura 35516, Egypt*

[11] *Department of Pharmaceutical Chemistry, Faculty of Pharmacy, Tanta University, Tanta 31527, Egypt*

[#] Equal contributions

[*] Correspondence: H.O.T. haytham.omar.mahmoud@pharm.tanta.edu.eg, D-G. S.; dsong82@kist.re.kr, W.M.E.; wagdy2000@gmail.com.

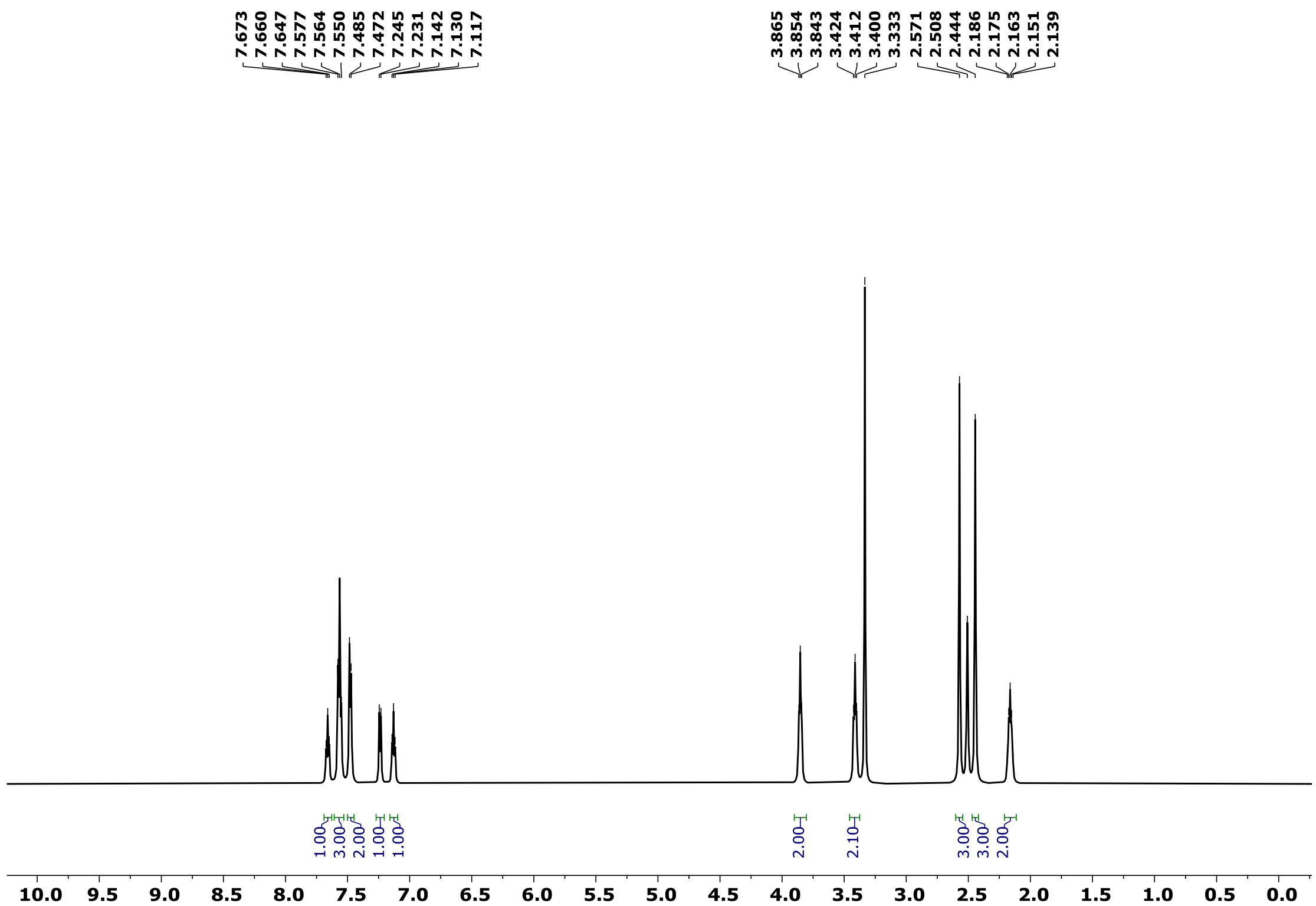

Figure S1. ^{1}H NMR (600 MHz, DMSO-d_6) spectrum of compound **9a**

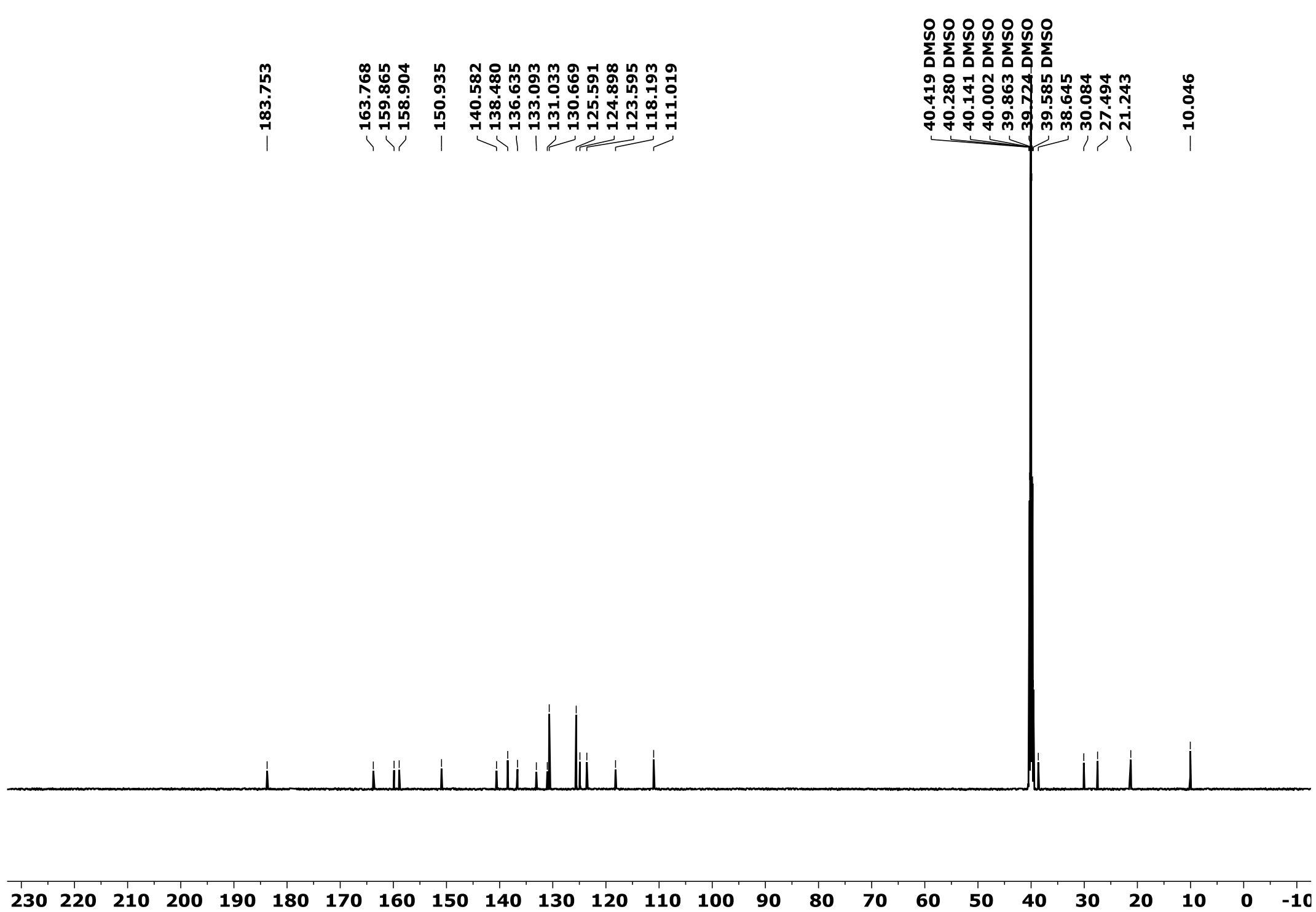

Figure S2. ^{13}C NMR (151 MHz, DMSO) spectrum of compound **9a**

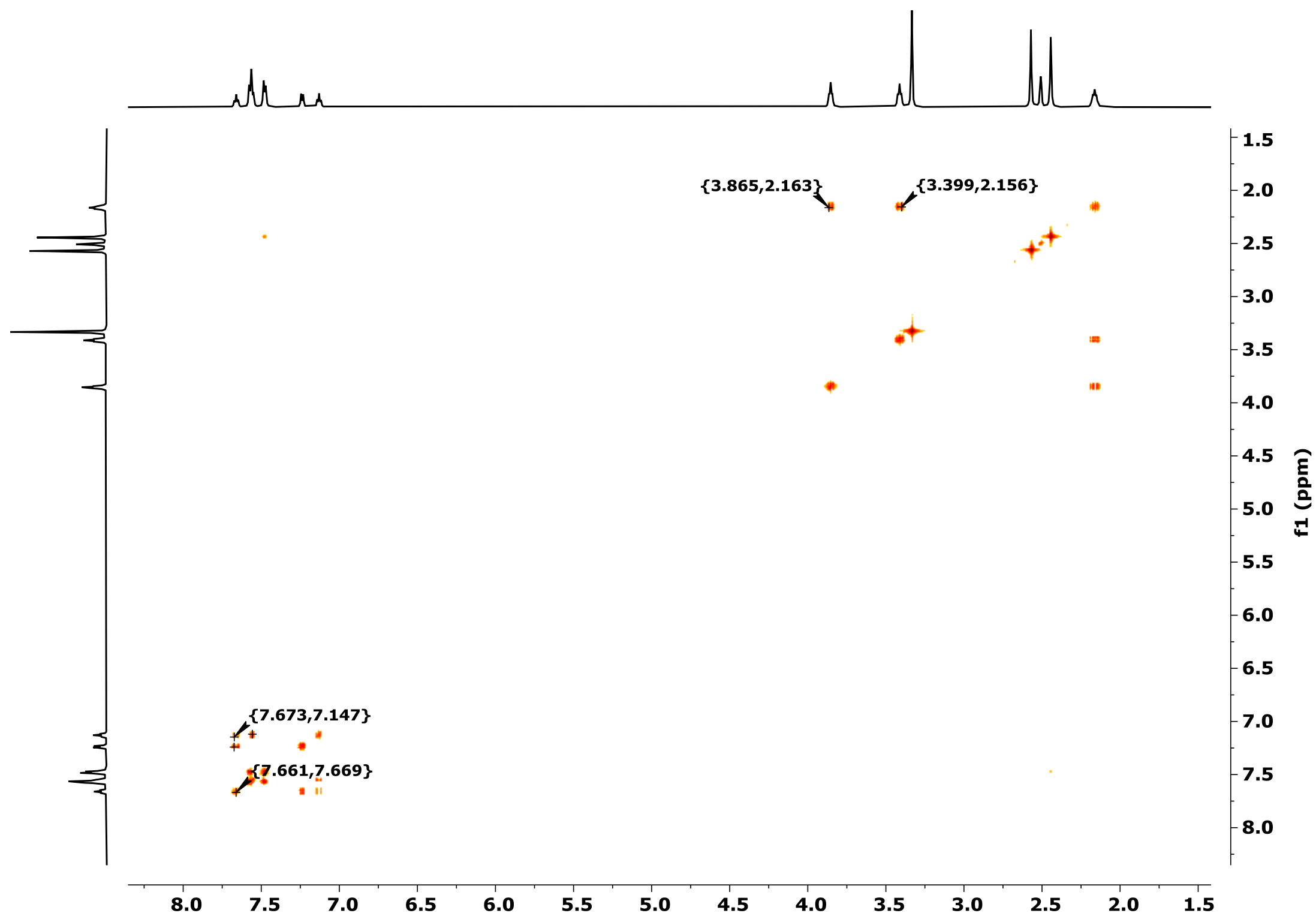

Figure S3. ^{1}H-^{1}H COSY spectrum (600 MHz, DMSO-d_6) of compound **9a**

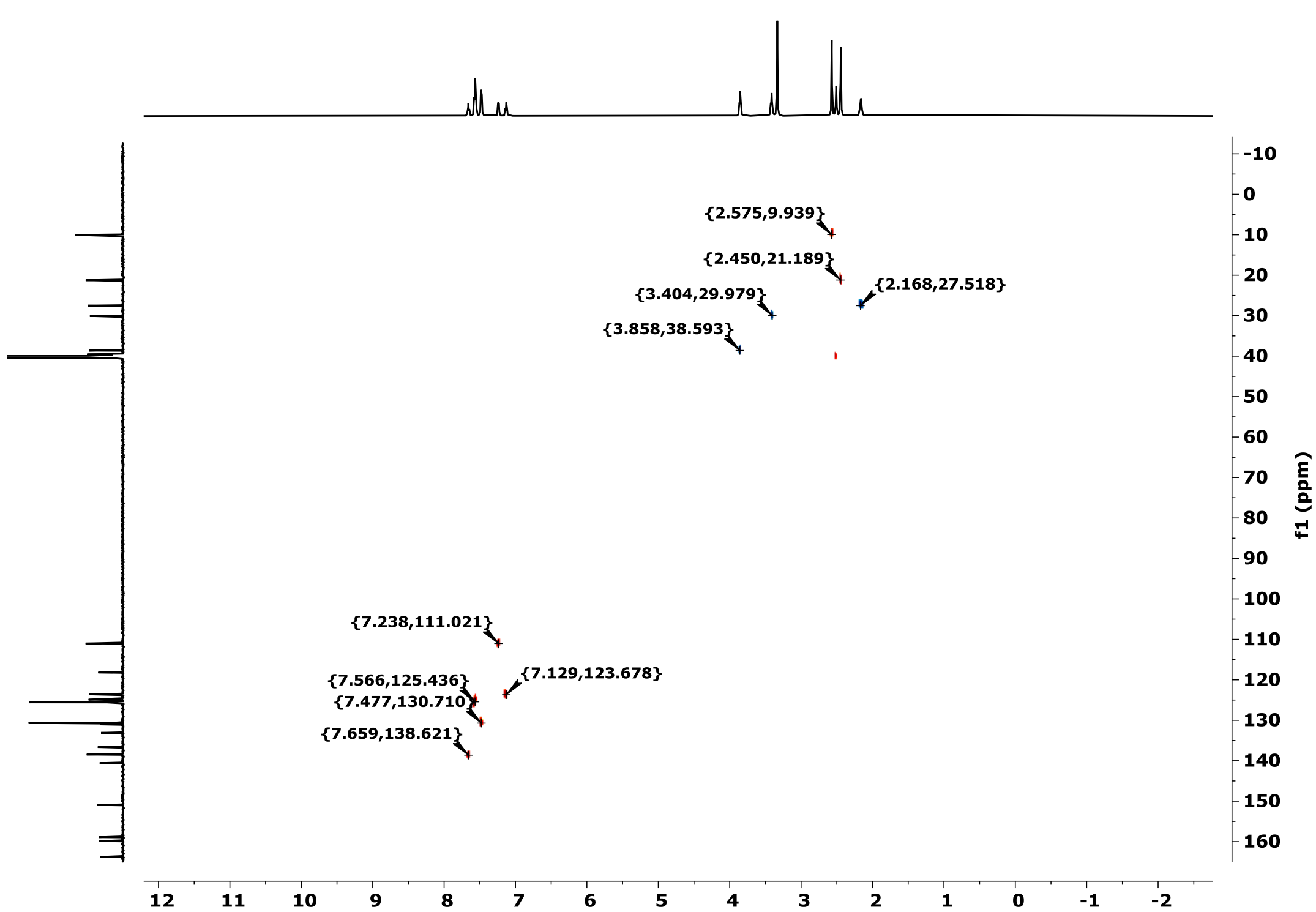

Figure S4. 2D ^{1}H-^{13}C HMQC spectrum (600/151 MHz, DMSO-d_6) of compound **9a**

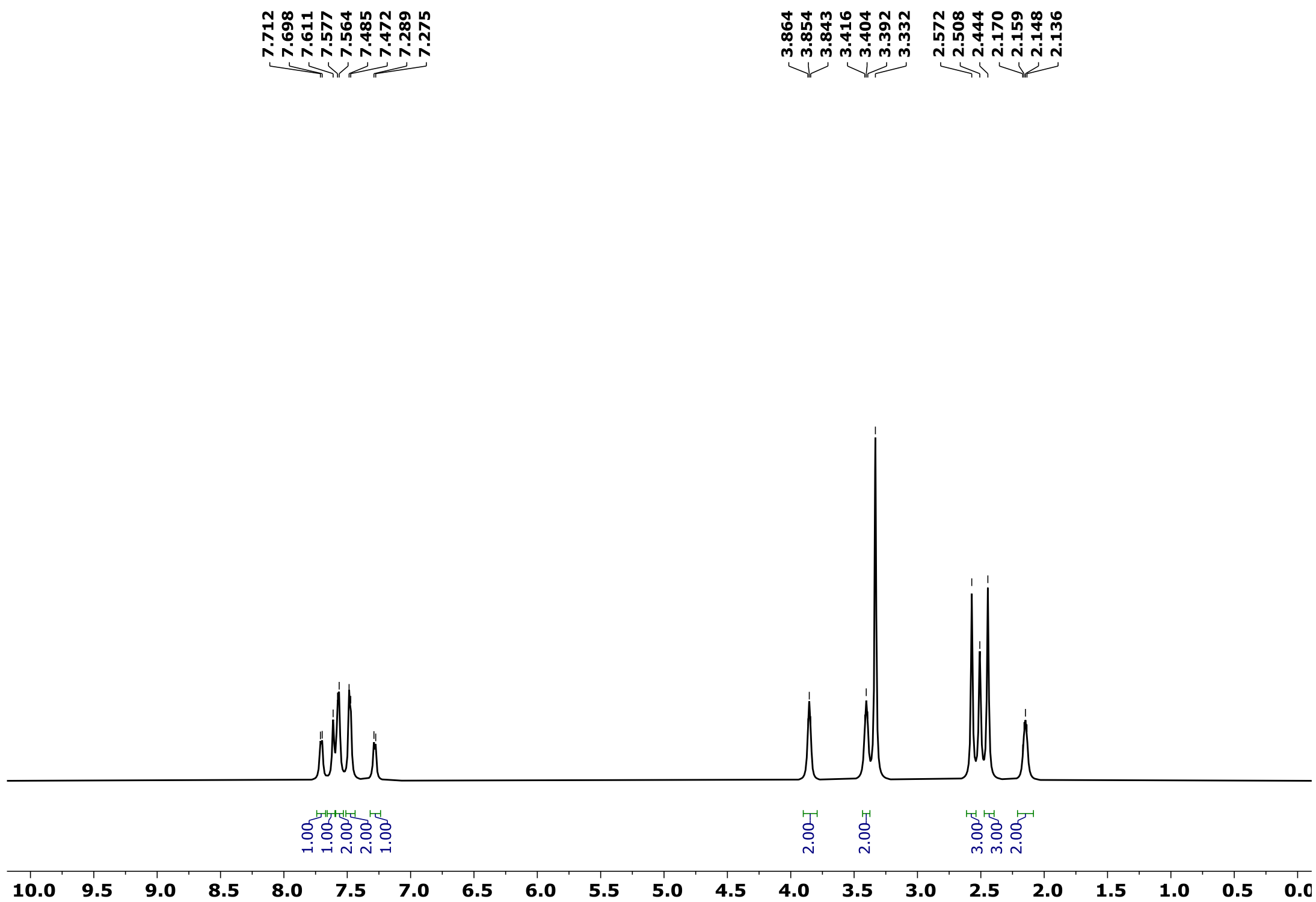

Figure S5. ^{1}H NMR (600 MHz, DMSO-d_6) spectrum of compound **9b**

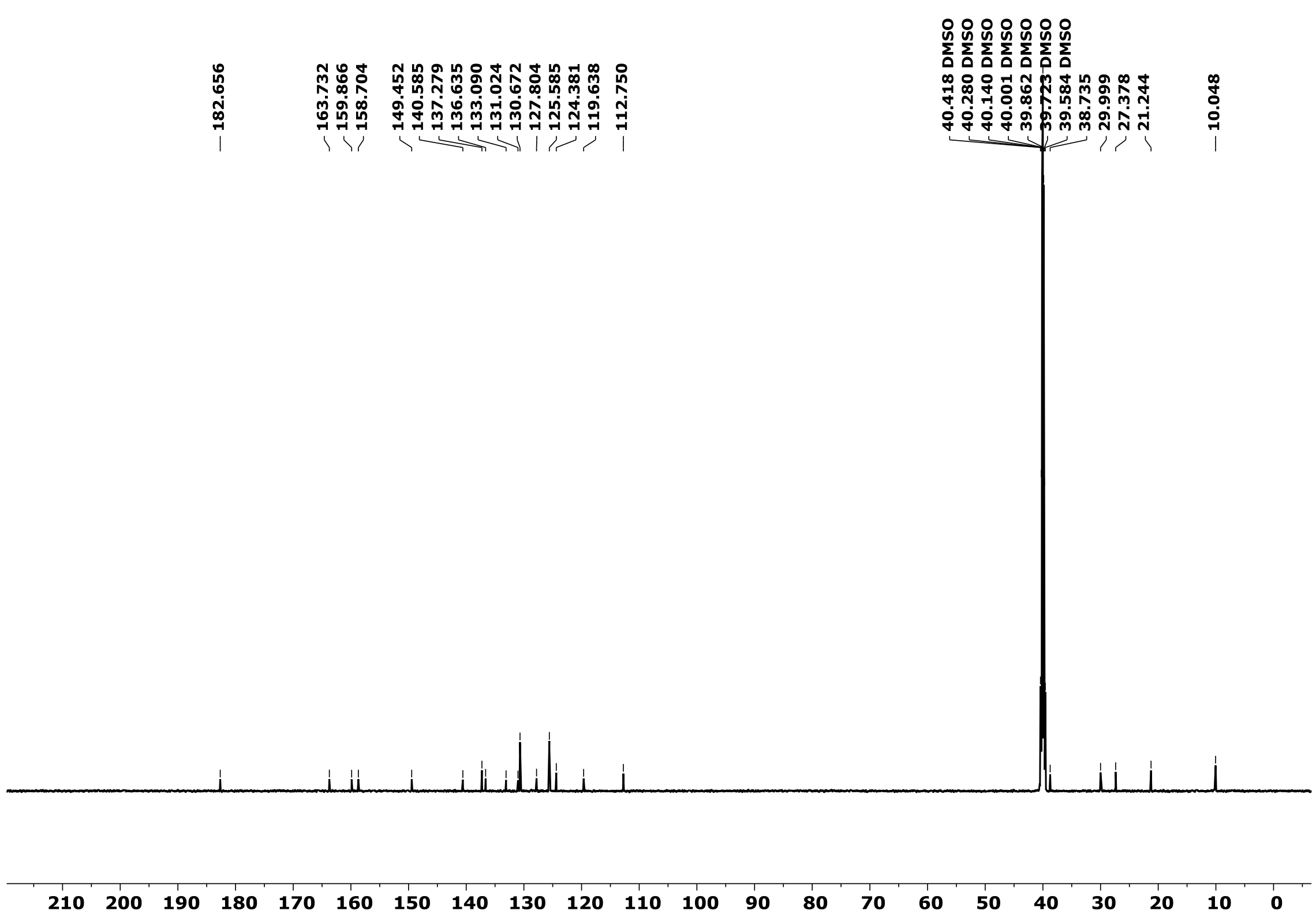

Figure S6. ^{13}C NMR (151 MHz, DMSO) spectrum of compound **9b**

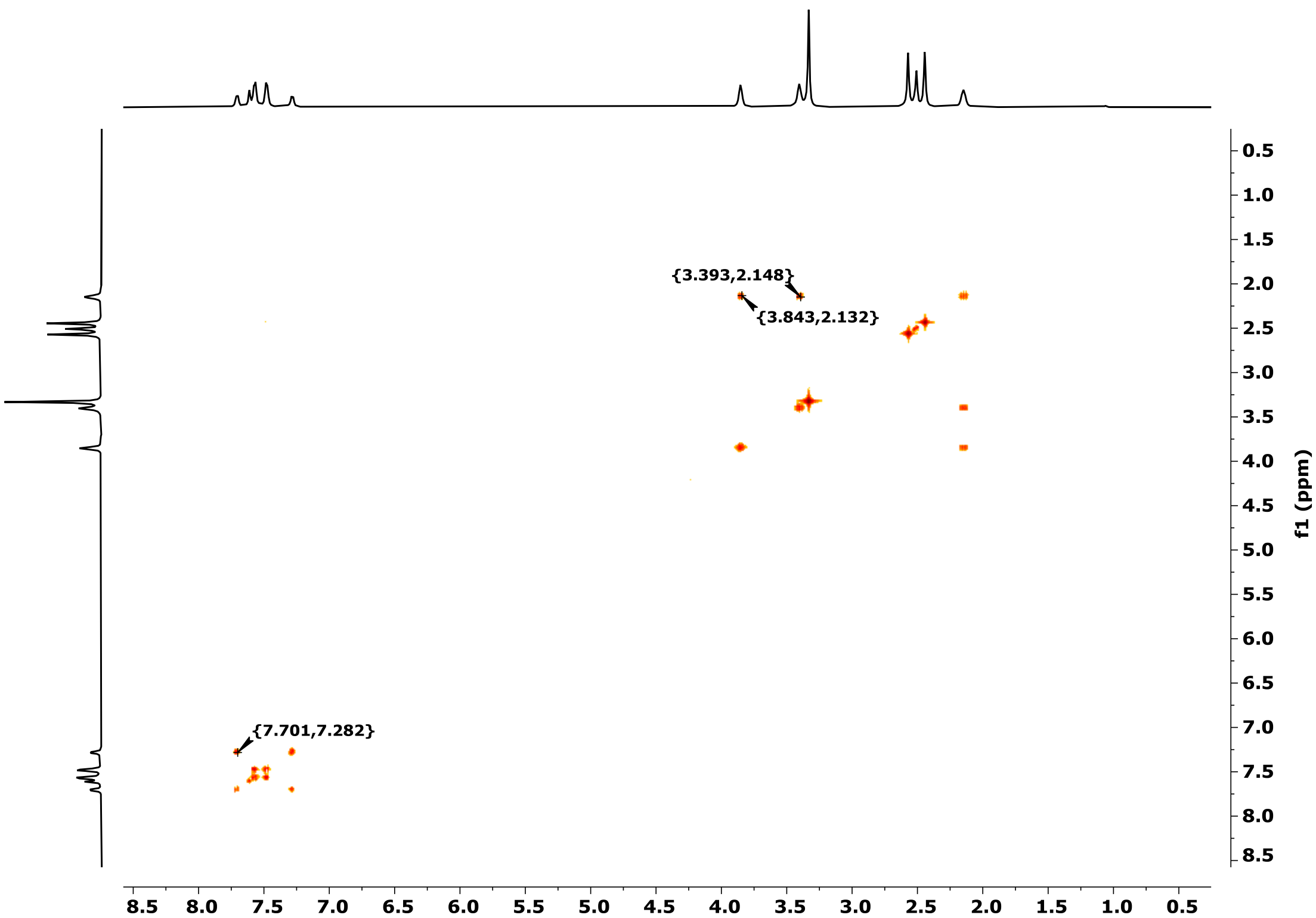

Figure S7. ^{1}H-^{1}H COSY spectrum (600 MHz, DMSO-d_6) of compound **9b**

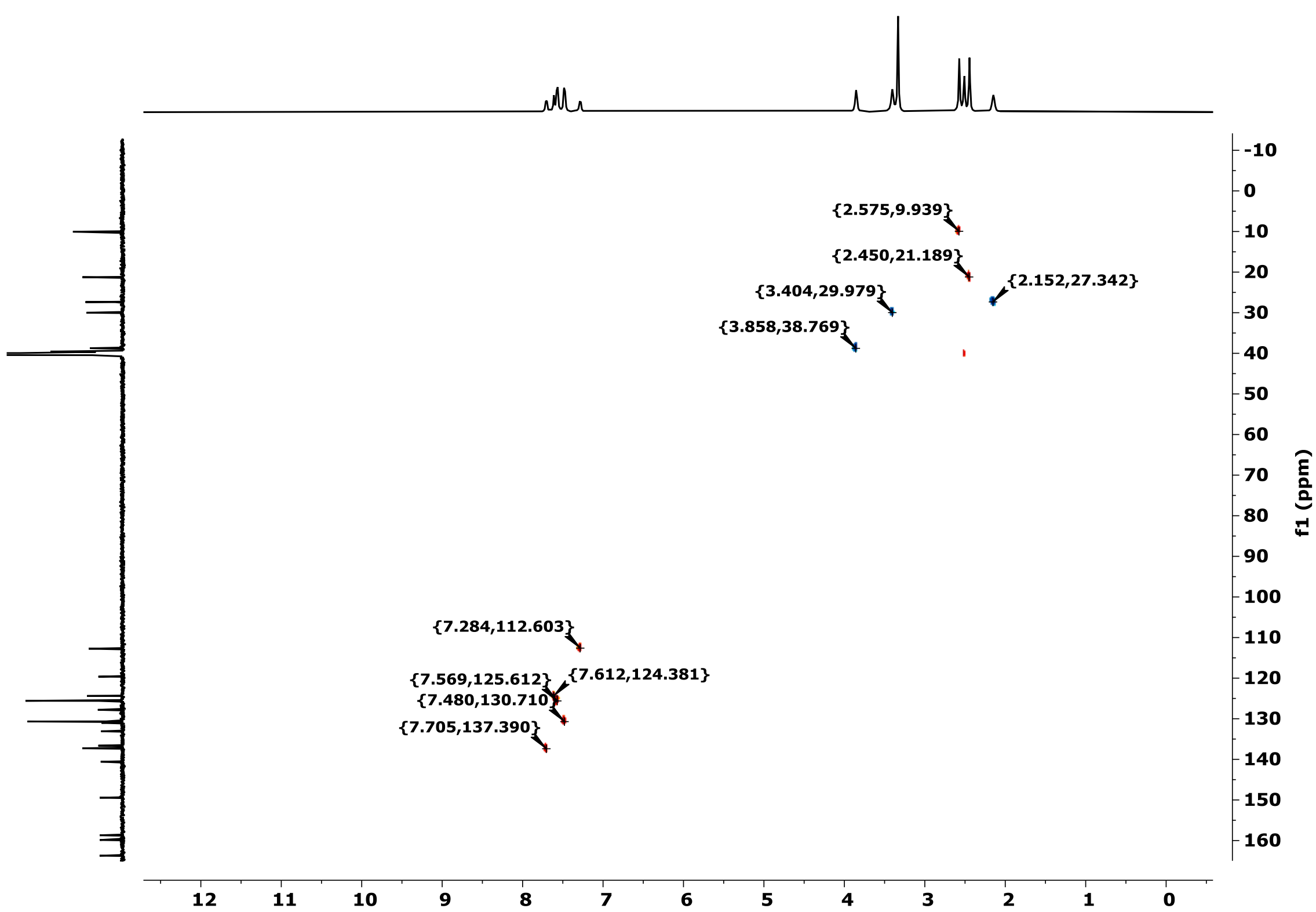

Figure S8. 2D ^{1}H-^{13}C HMQC spectrum (600/151 MHz, DMSO-d_6) of compound **9b**

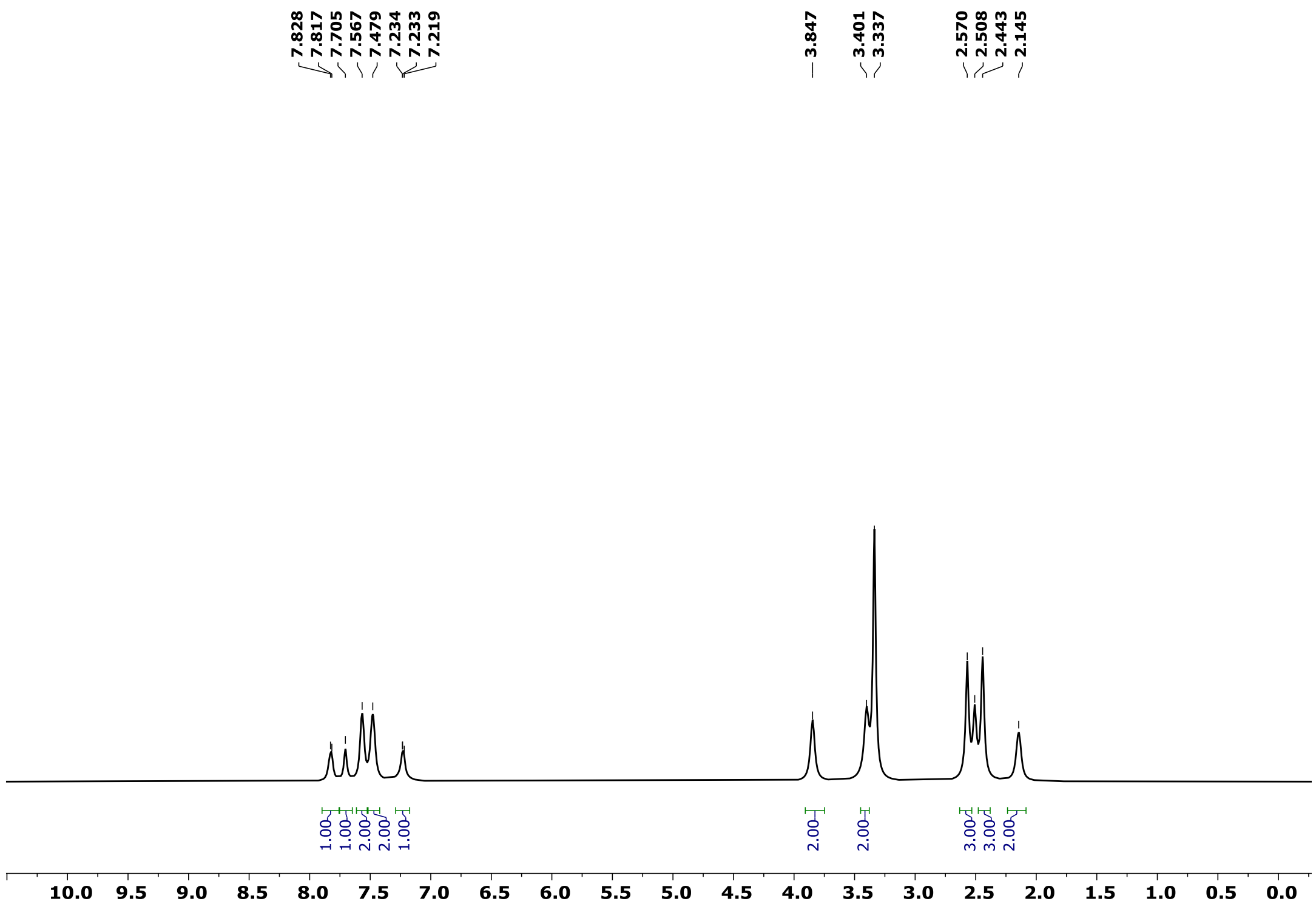

Figure S9. ^{1}H NMR (600 MHz, DMSO-d_6) spectrum of compound **9c**

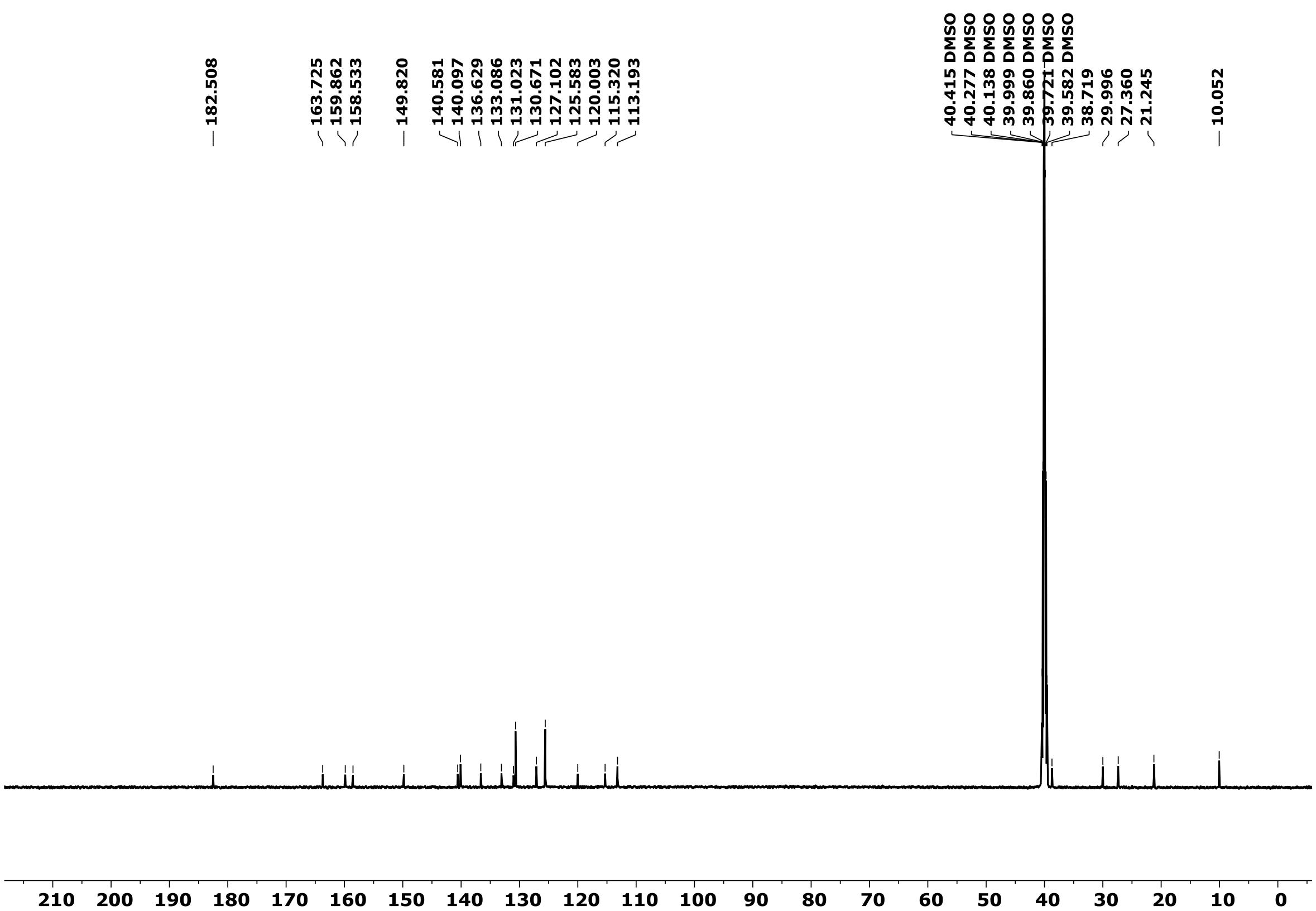

Figure S10. ^{13}C NMR (151 MHz, DMSO) spectrum of compound **9c**

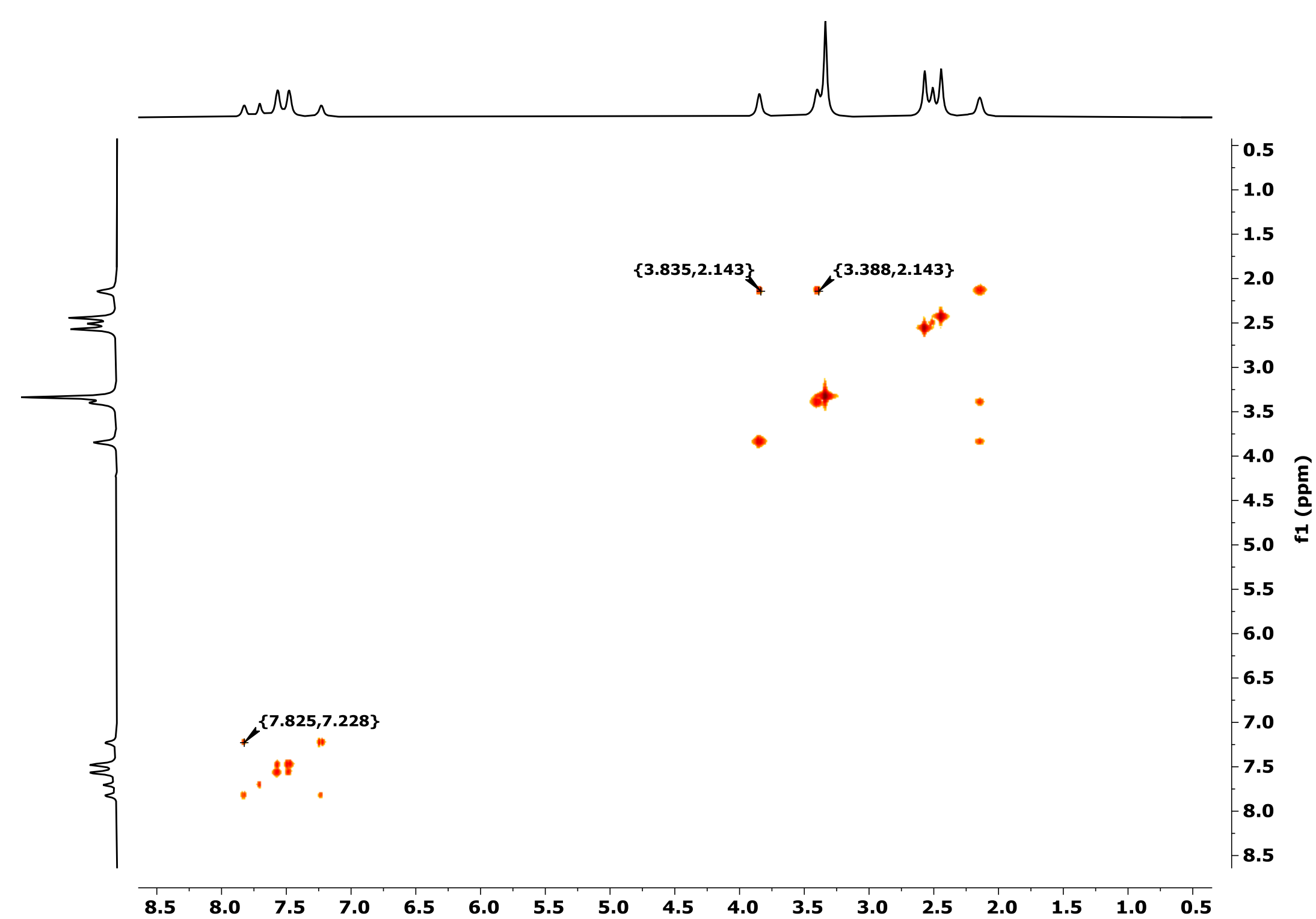

Figure S11. ^{1}H-^{1}H COSY spectrum (600 MHz, DMSO-d_6) of compound **9c**

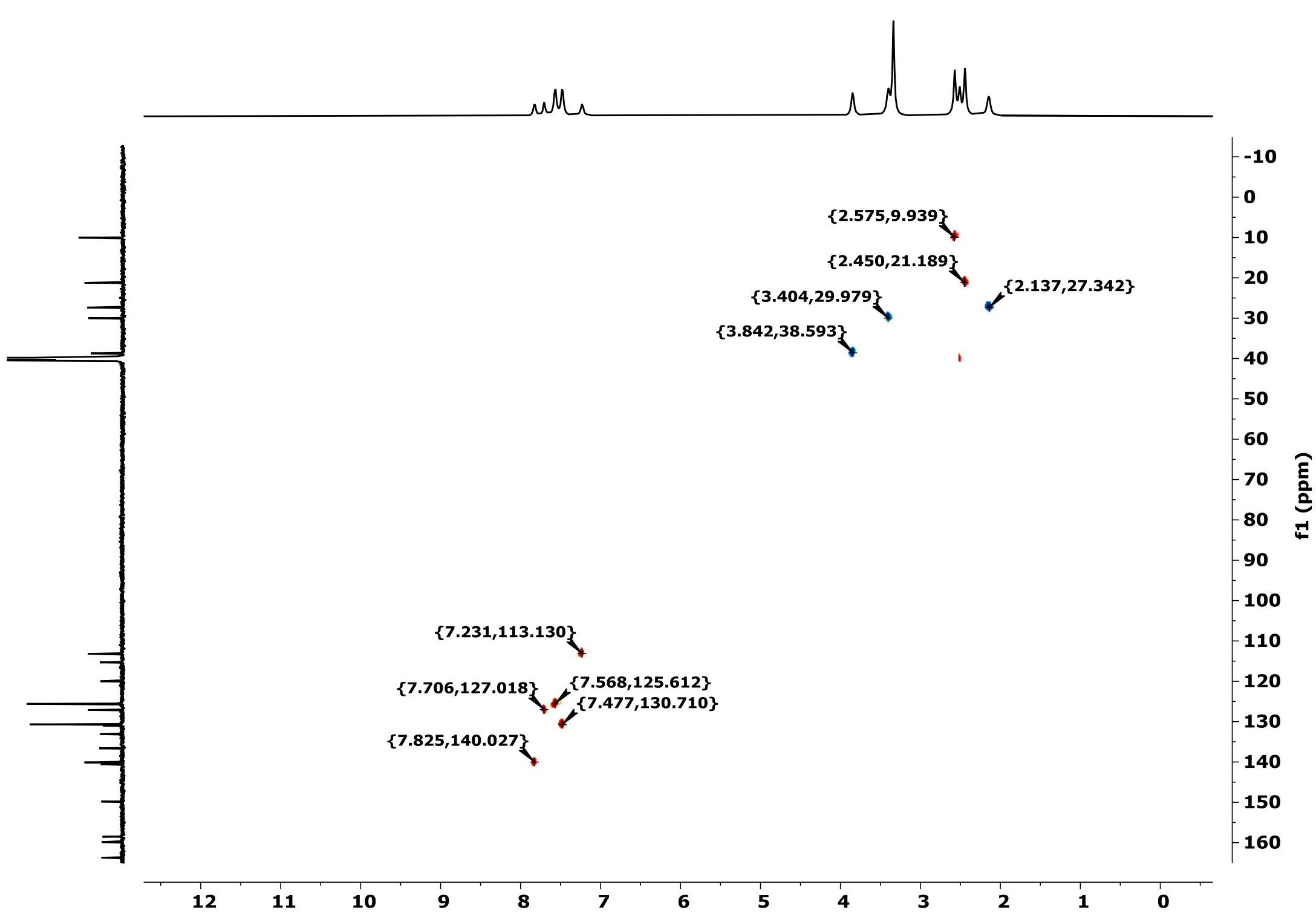

Figure S12. 2D ^{1}H-^{13}C HMQC spectrum (600/151 MHz, DMSO-d_6) of compound **9c**

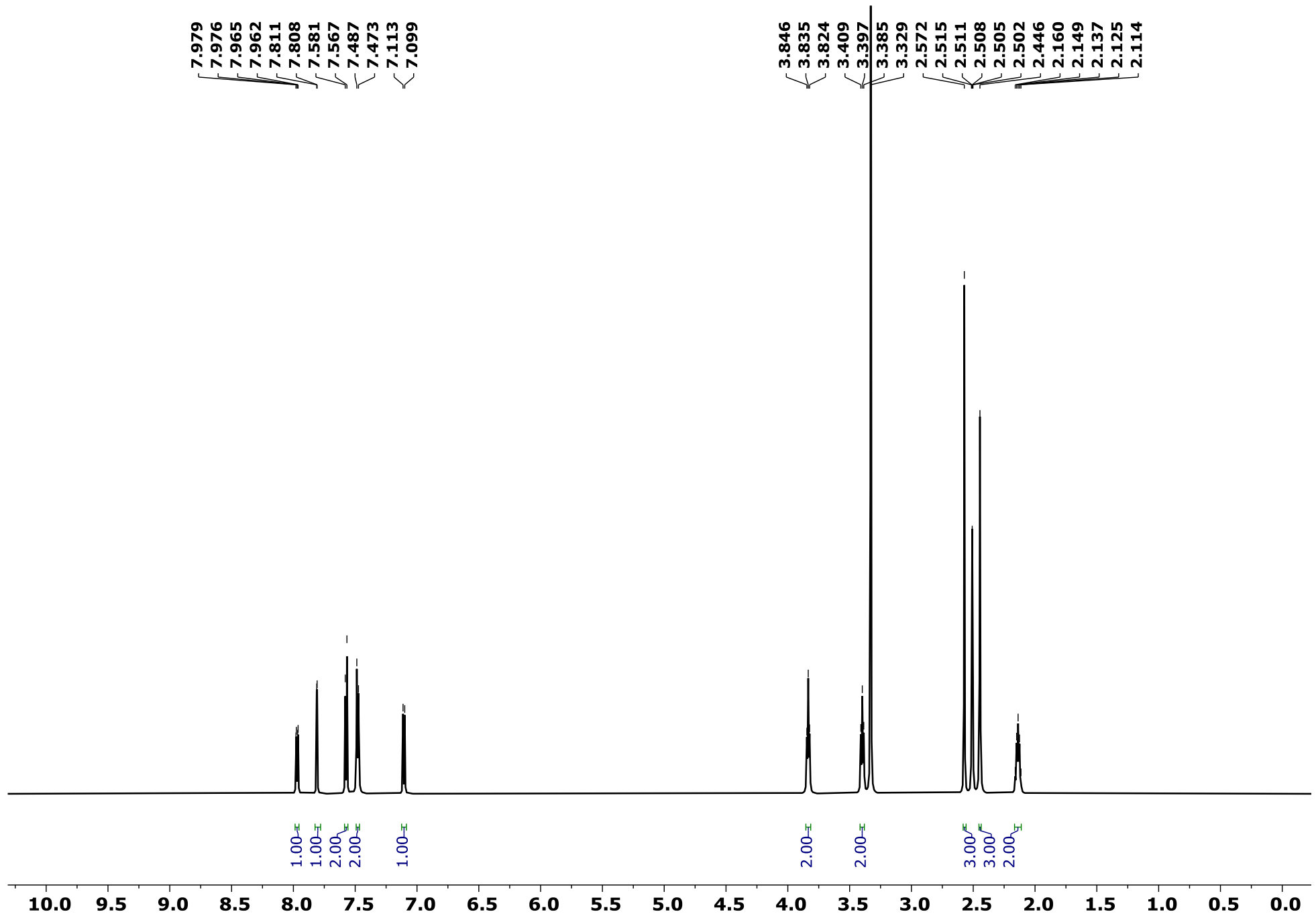

Figure S13. ^{1}H NMR (600 MHz, DMSO-d_6) spectrum of compound **9d**

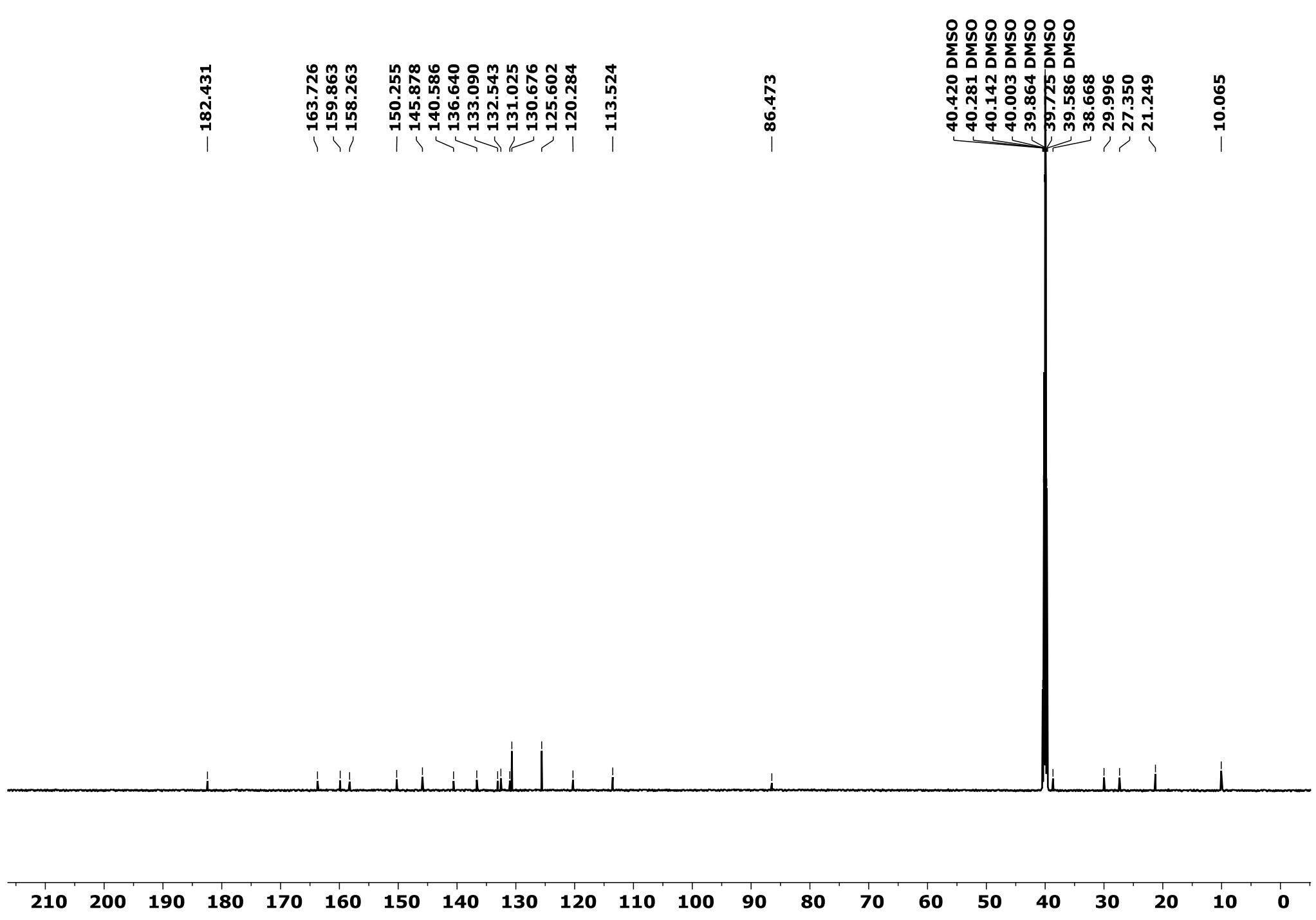

Figure S14. ^{13}C NMR (151 MHz, DMSO) spectrum of compound **9d**

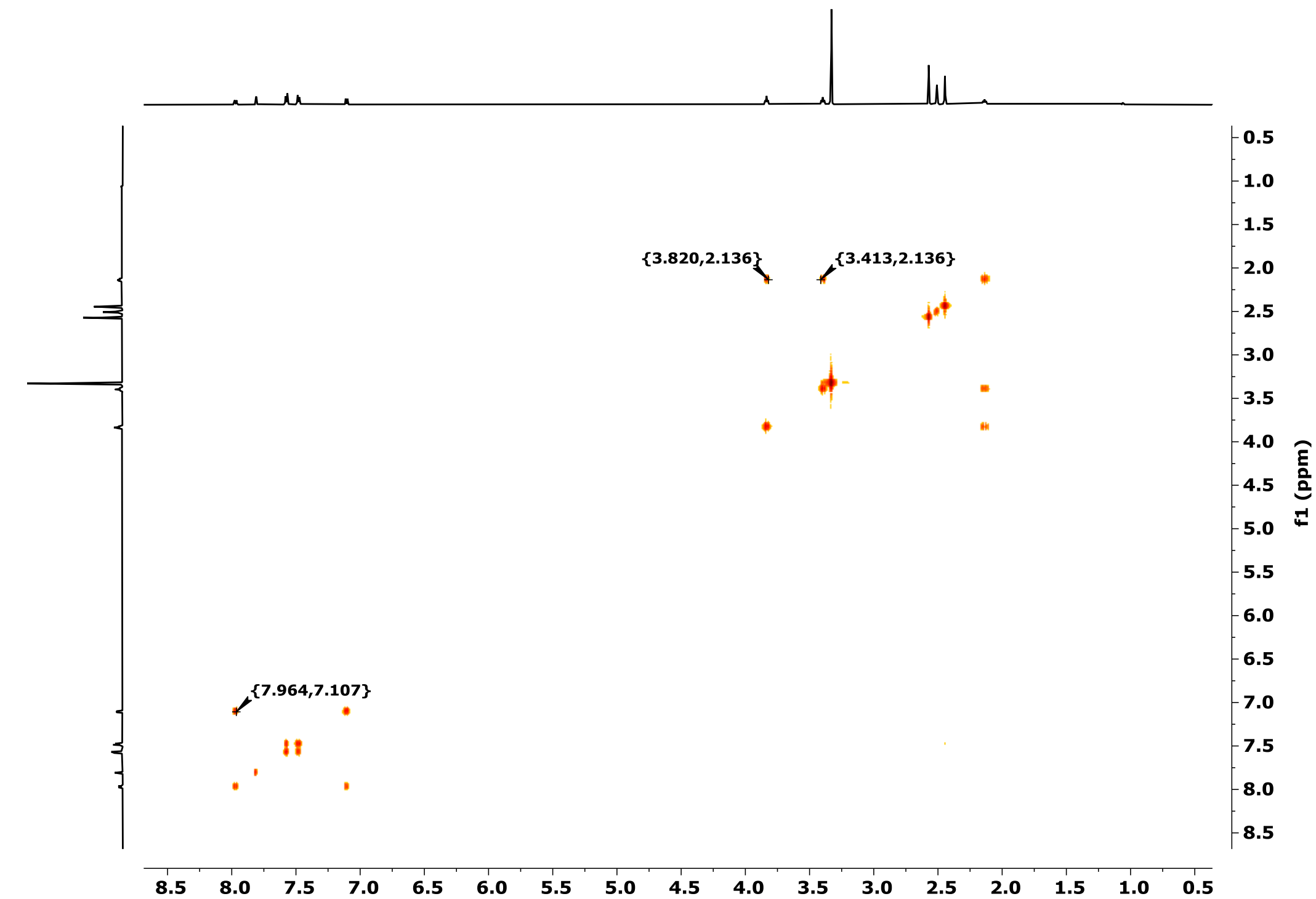

Figure S15. ^1H-^1H COSY spectrum (600 MHz, DMSO-d_6) of compound **9d**

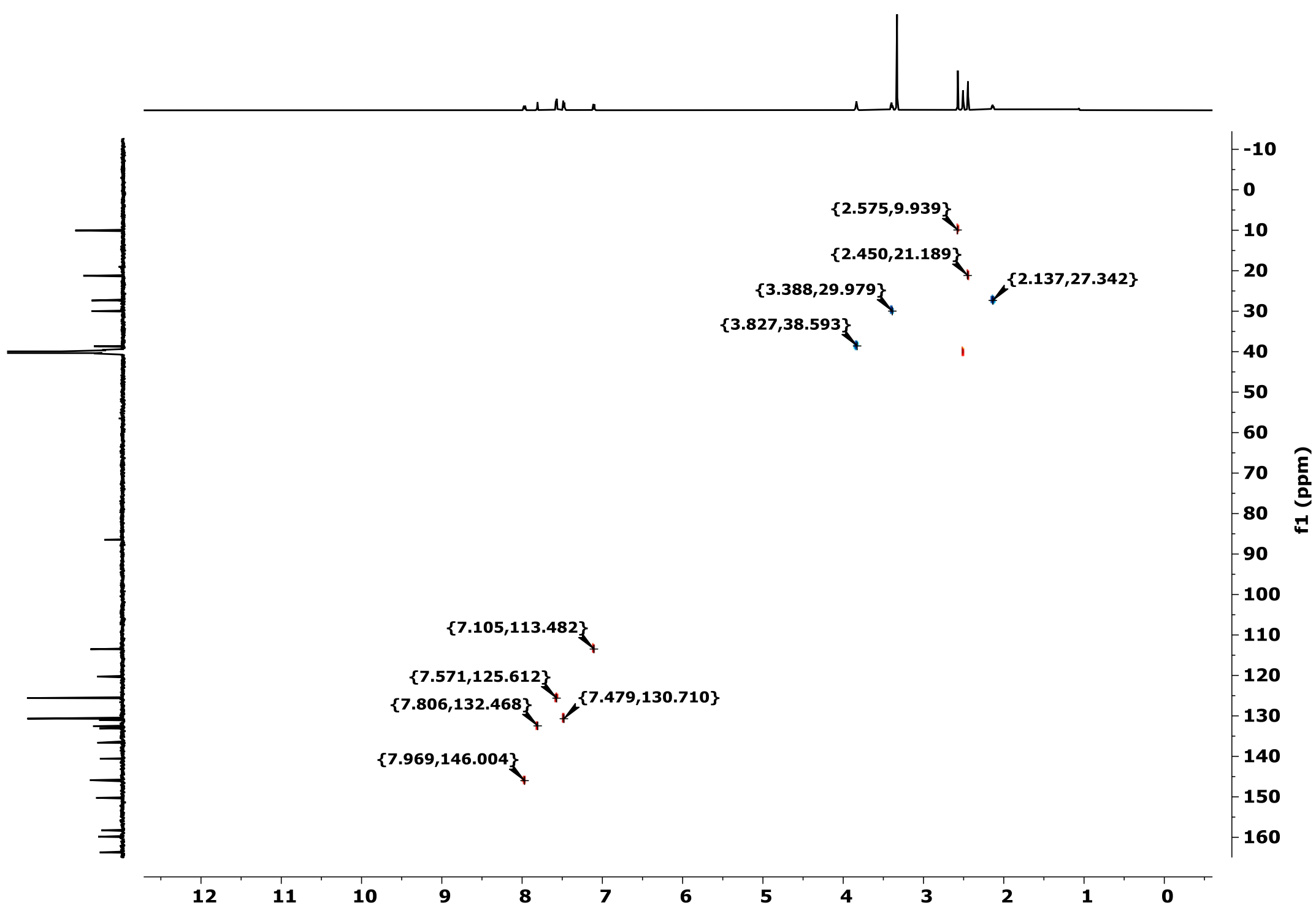

Figure S16. 2D ^1H-^{13}C HMQC spectrum (600/151 MHz, DMSO-d_6) of compound **9d**

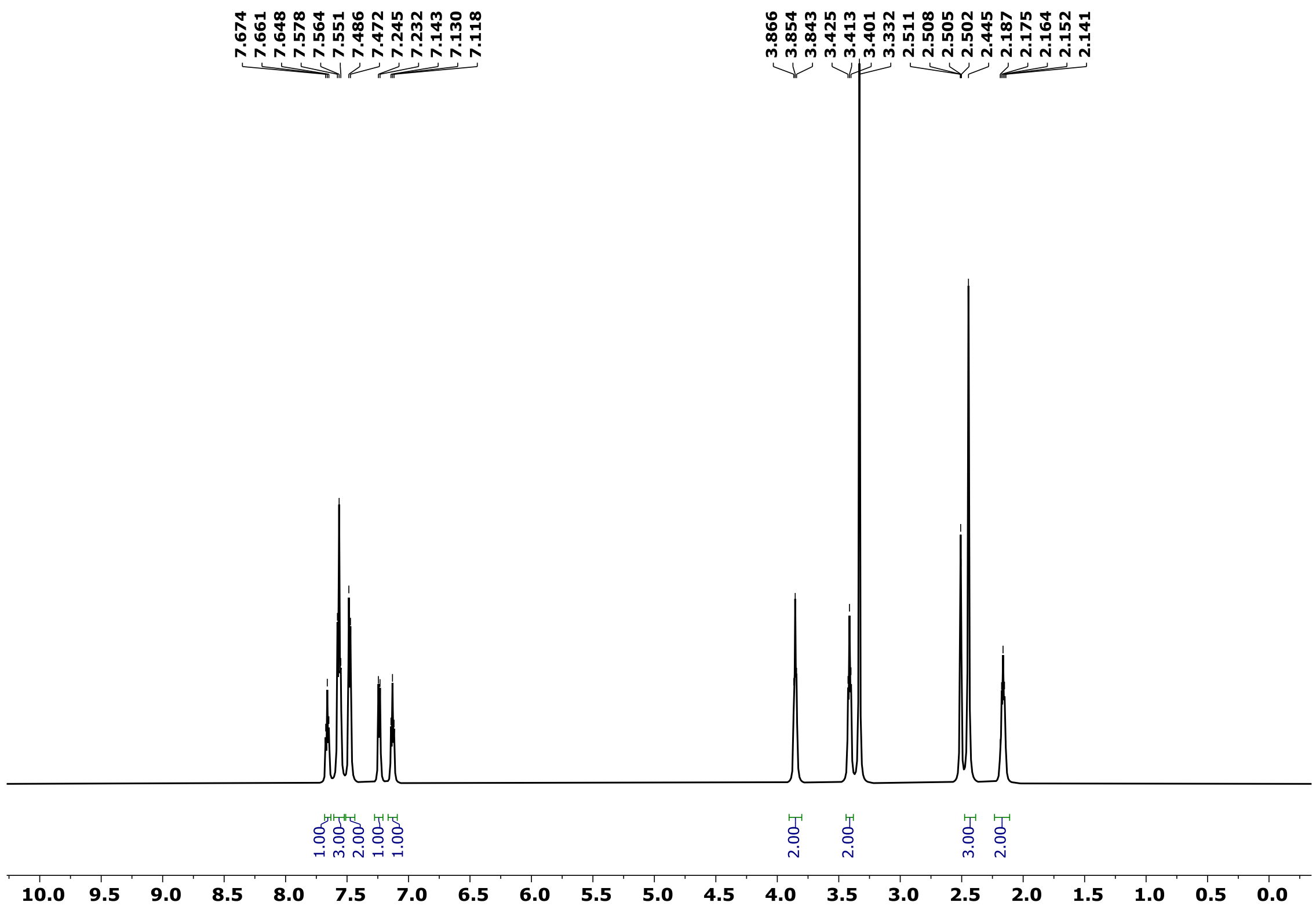

Figure S17. ^{1}H NMR (600 MHz, DMSO-d_6) spectrum of compound **9e**

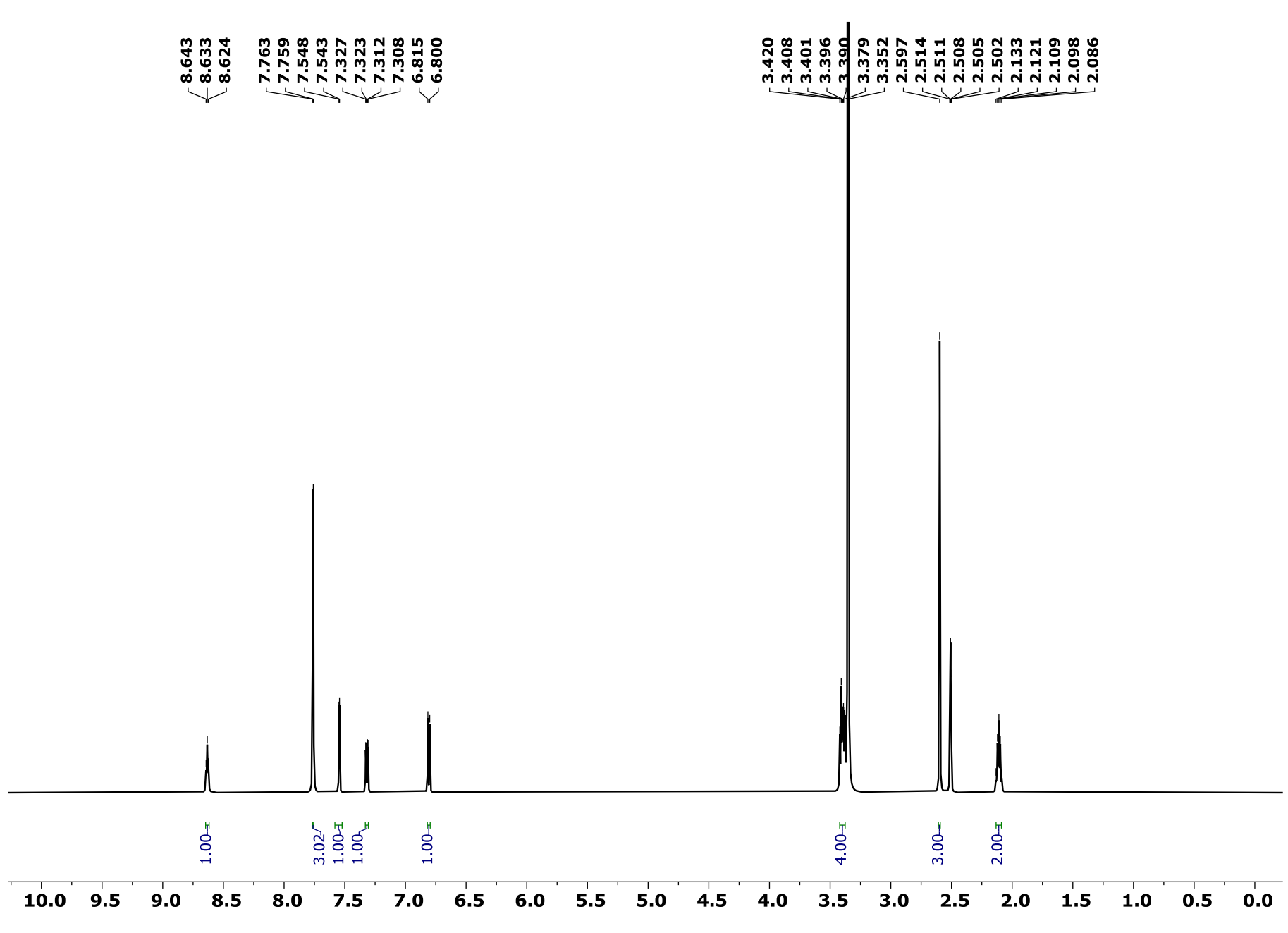

Figure S18. ^{1}H NMR (600 MHz, DMSO-d_6) spectrum of compound **9f**

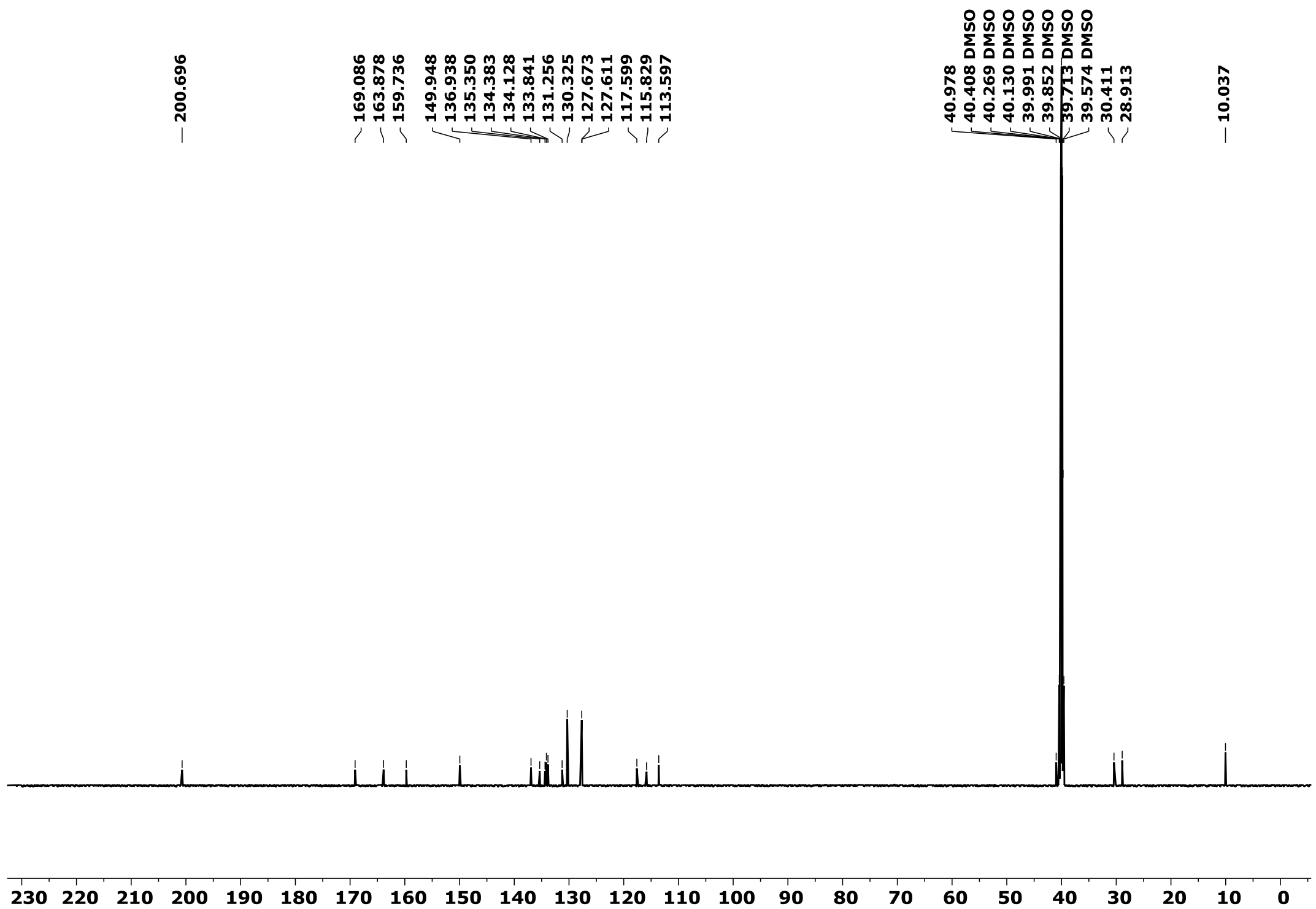

Figure S19. ^{13}C NMR (151 MHz, DMSO) spectrum of compound **9f**

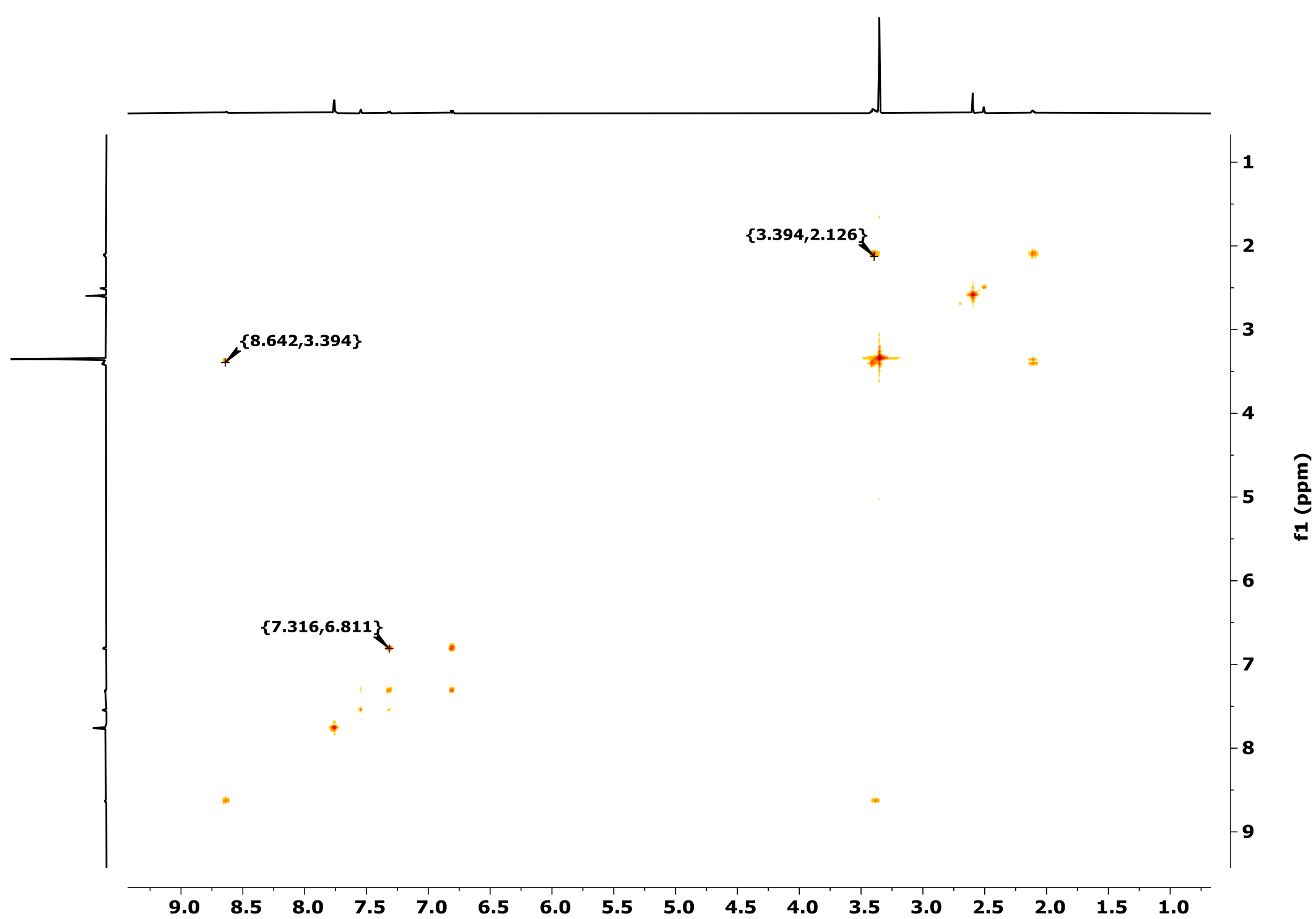

Figure S20. ^{1}H-^{1}H COSY spectrum (600 MHz, DMSO-d_6) of compound **9f**

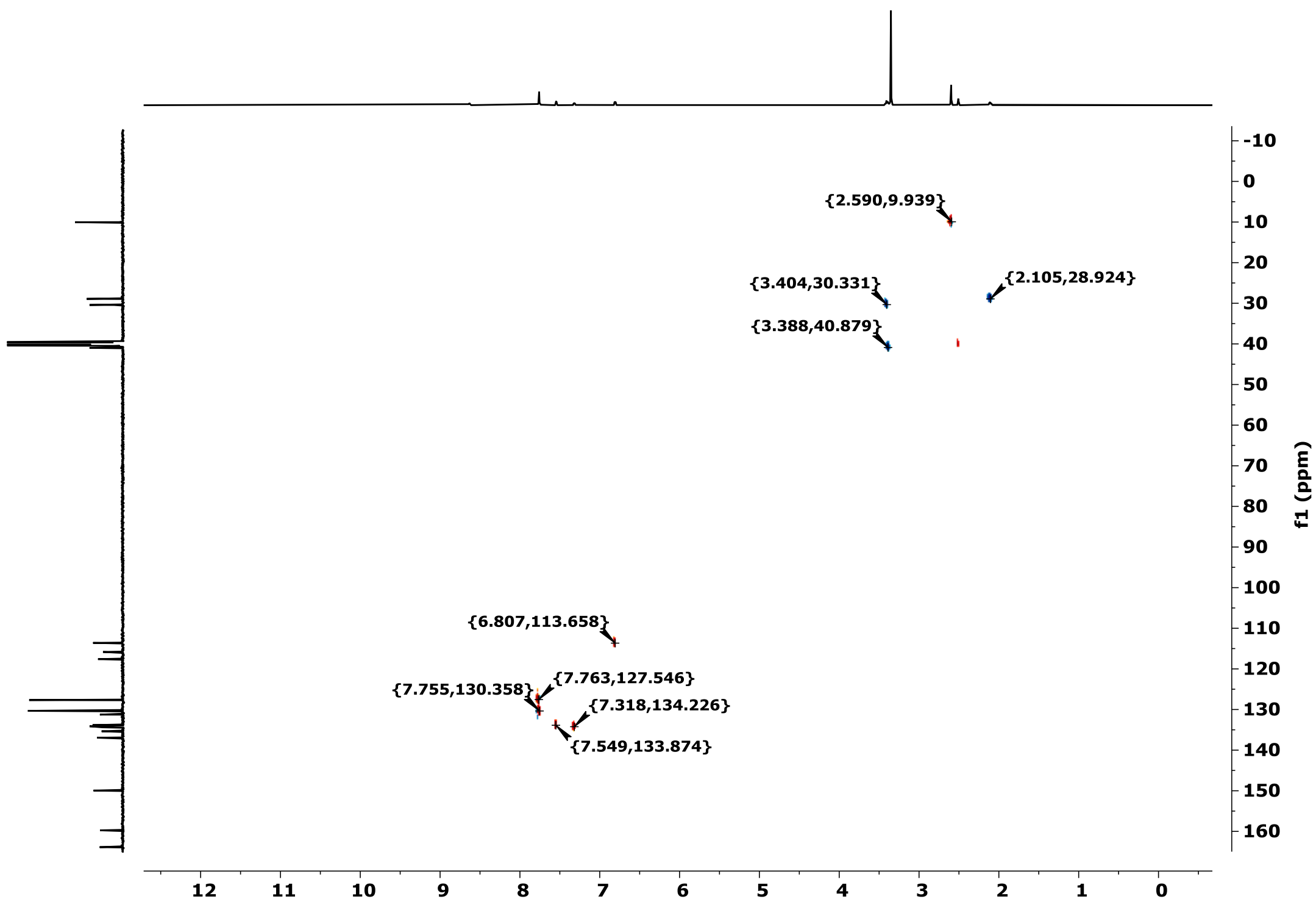

Figure S21. 2D ^{1}H-^{13}C HMQC spectrum (600/151 MHz, DMSO-d_6) of compound **9f**

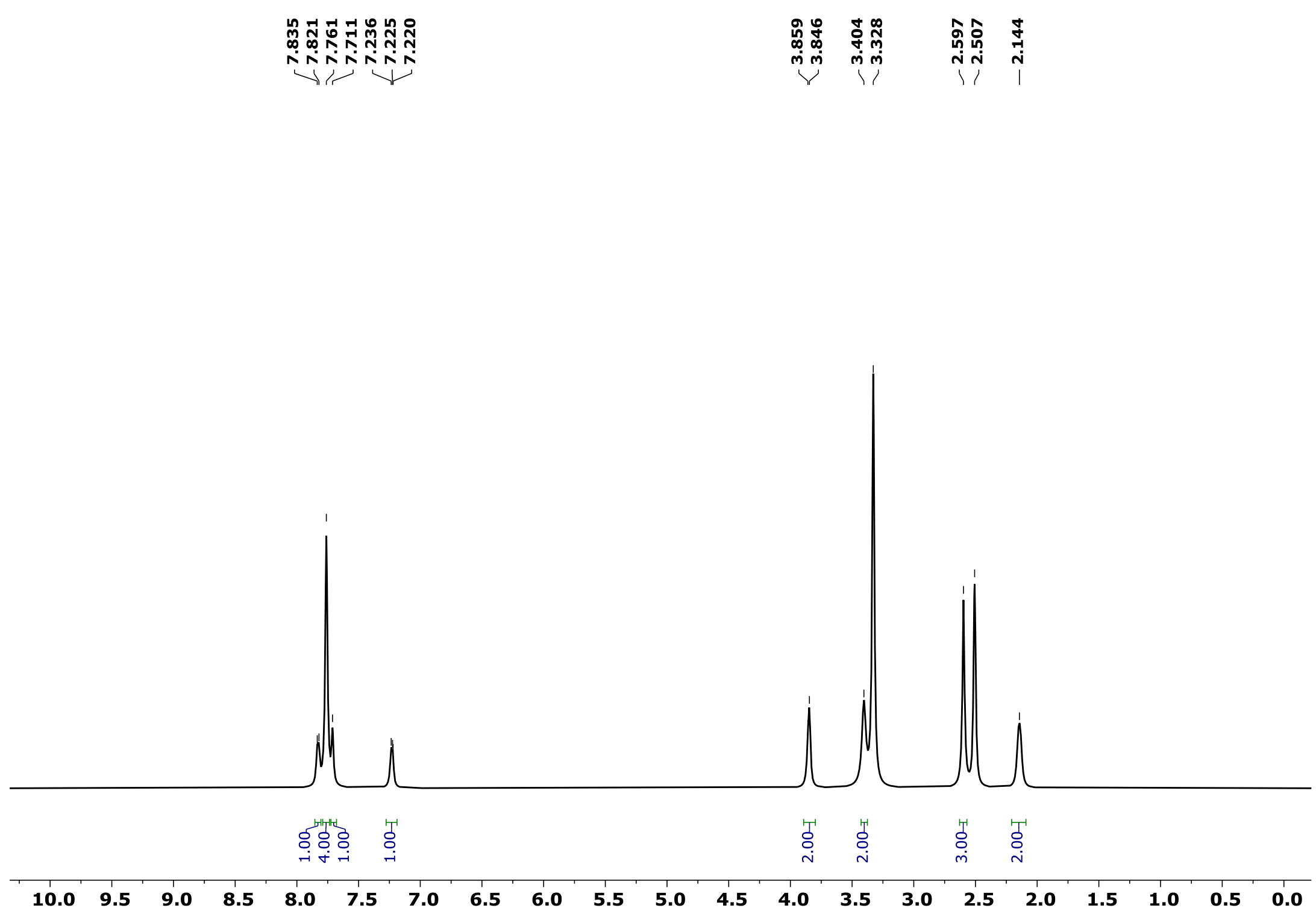

Figure S22. ^{1}H NMR (600 MHz, DMSO-d_6) spectrum of compound **9g**

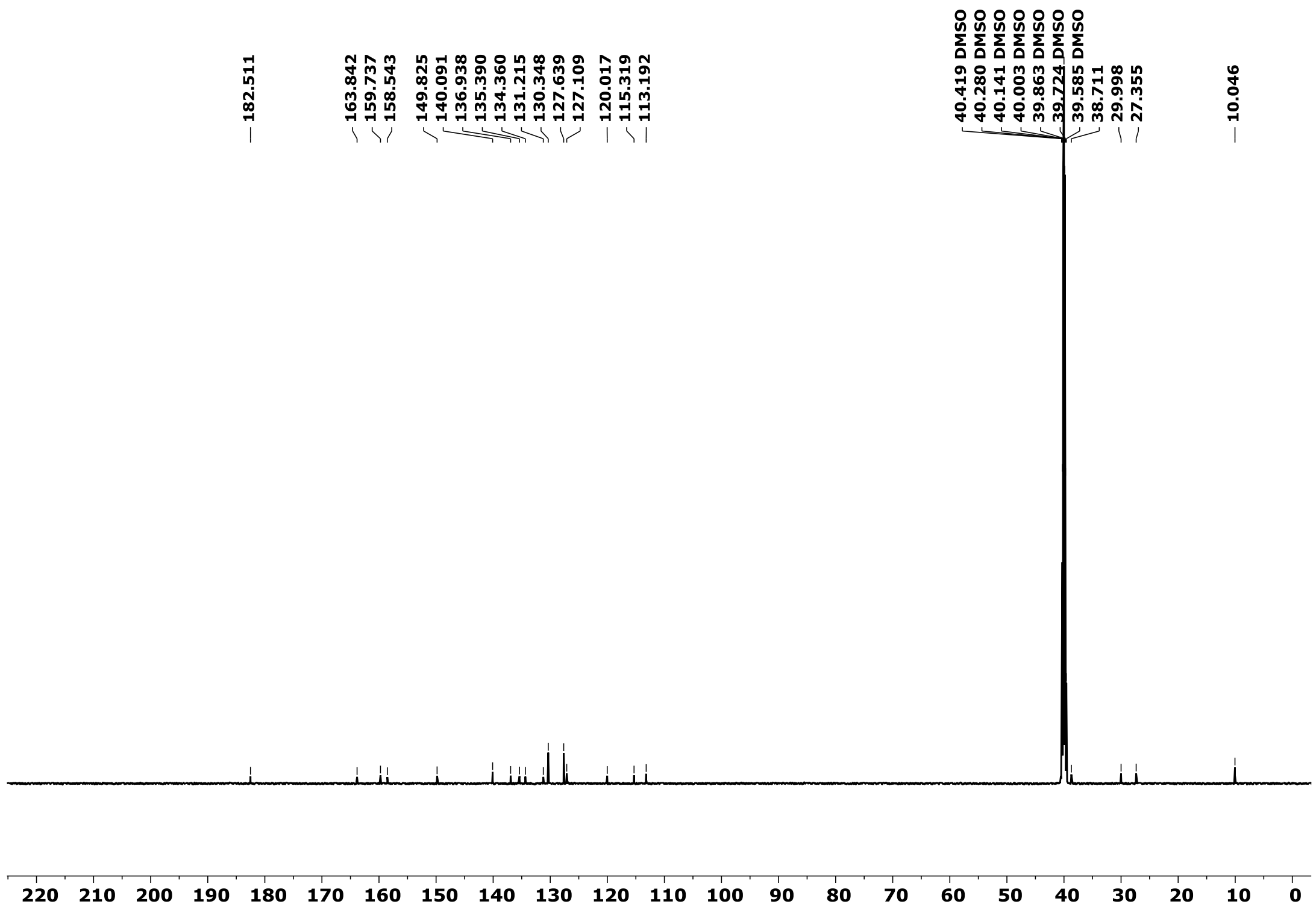

Figure S23. ^{13}C NMR (151 MHz, DMSO) spectrum of compound **9g**

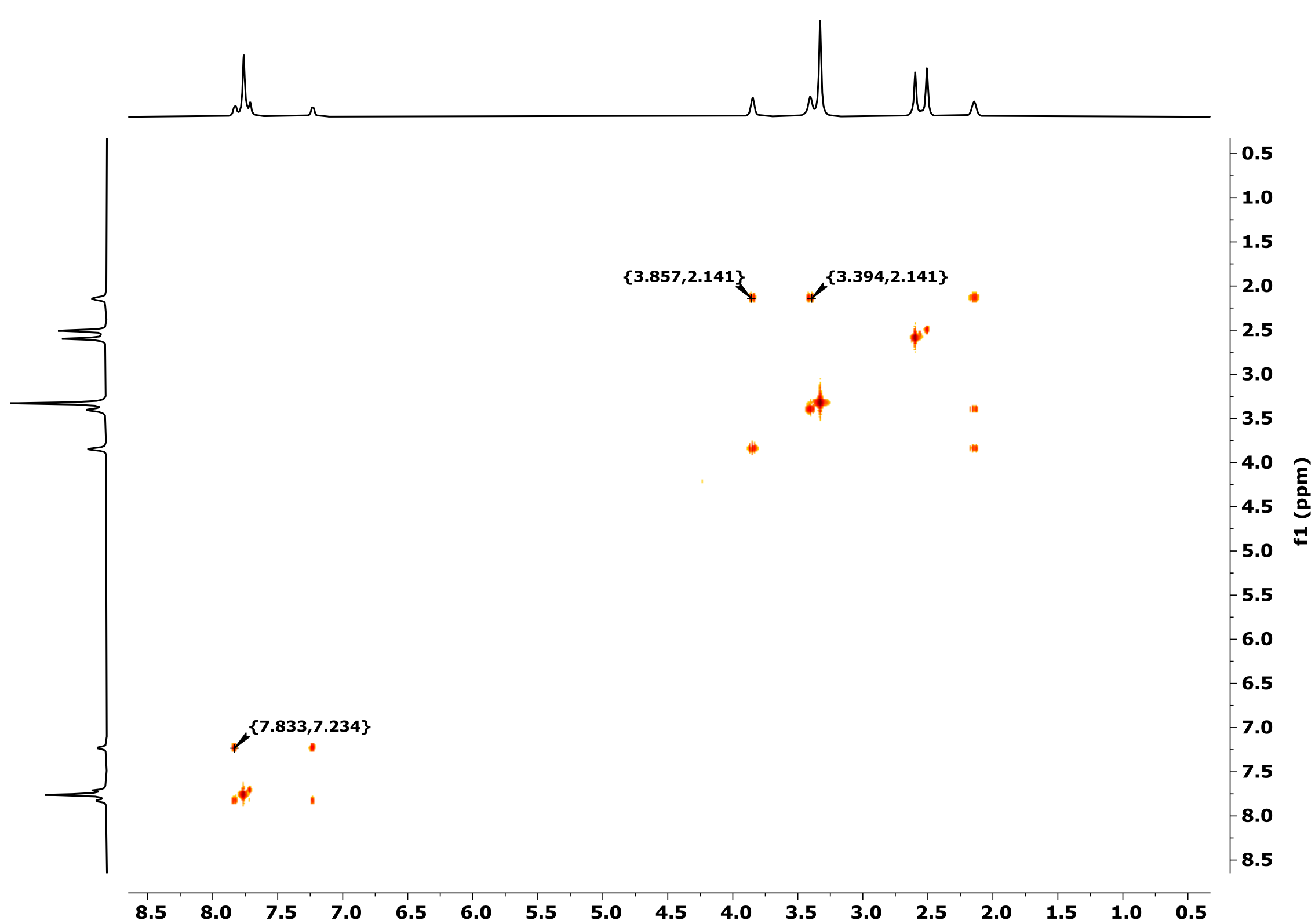

Figure S24. ^{1}H-^{1}H COSY spectrum (600 MHz, DMSO-d_6) of compound **9g**

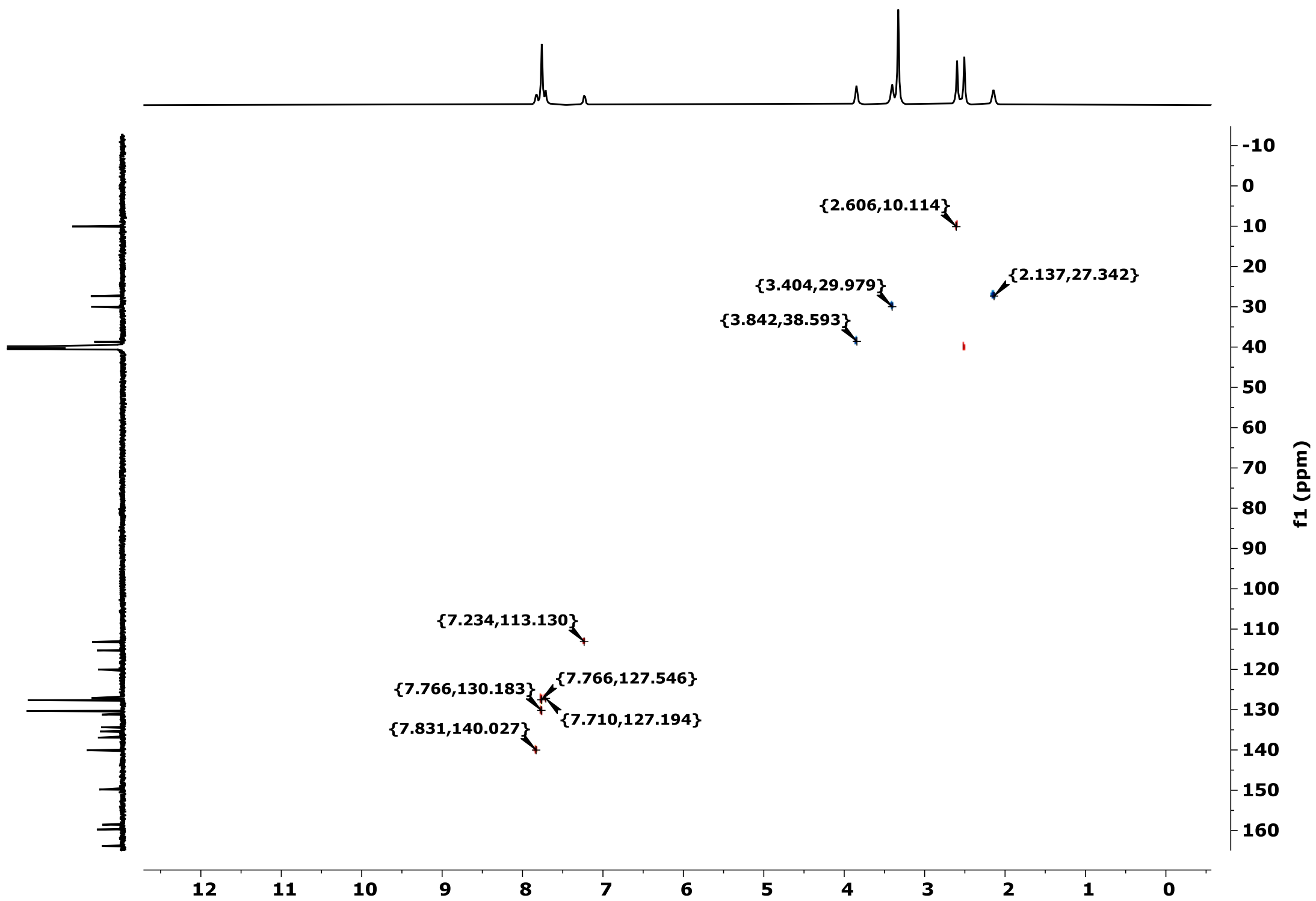

Figure S25. 2D ^{1}H-^{13}C HMQC spectrum (600/151 MHz, DMSO-d_6) of compound **9g**

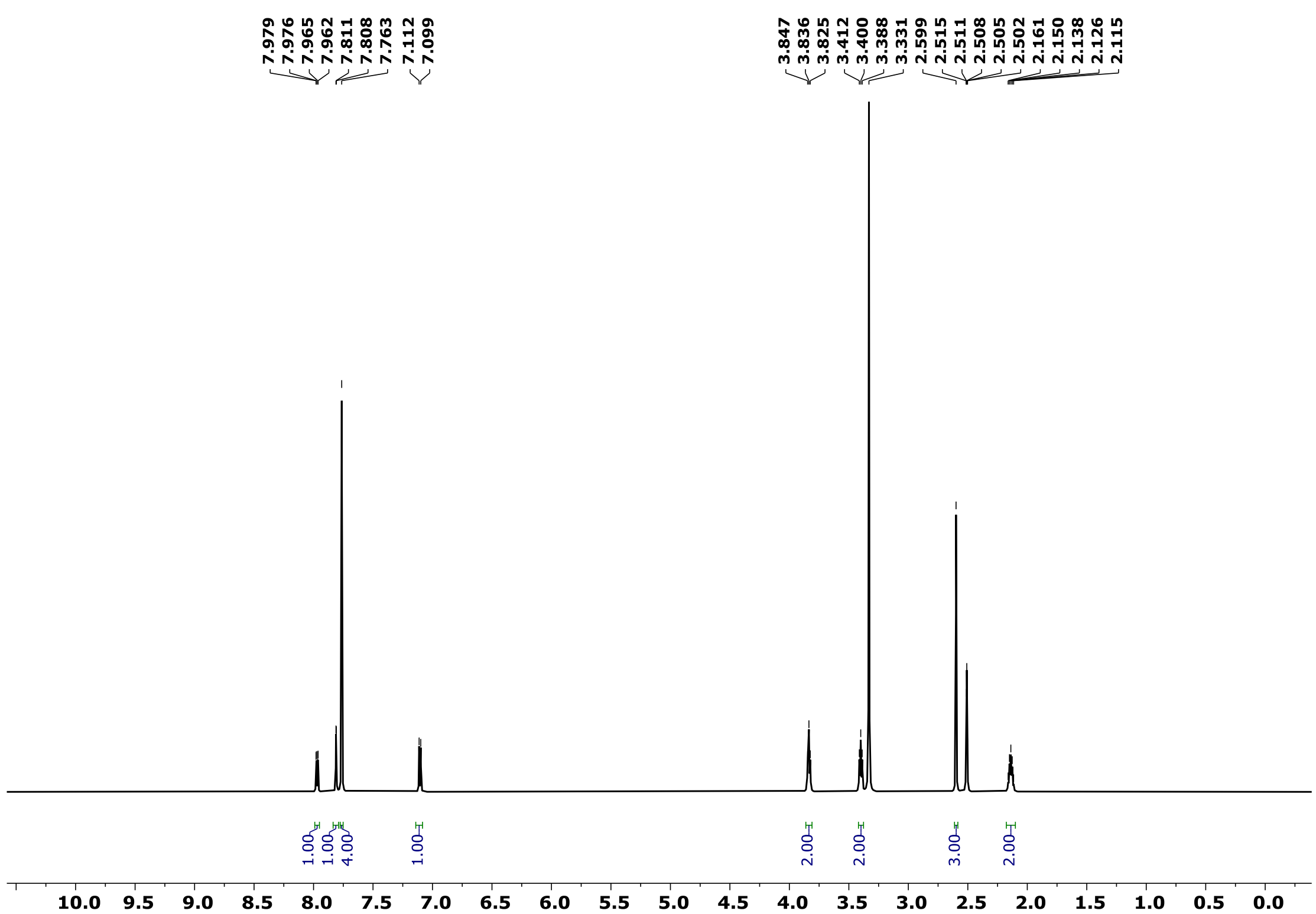

Figure S26. ^{1}H NMR (600 MHz, DMSO-d_6) spectrum of compound **9h**

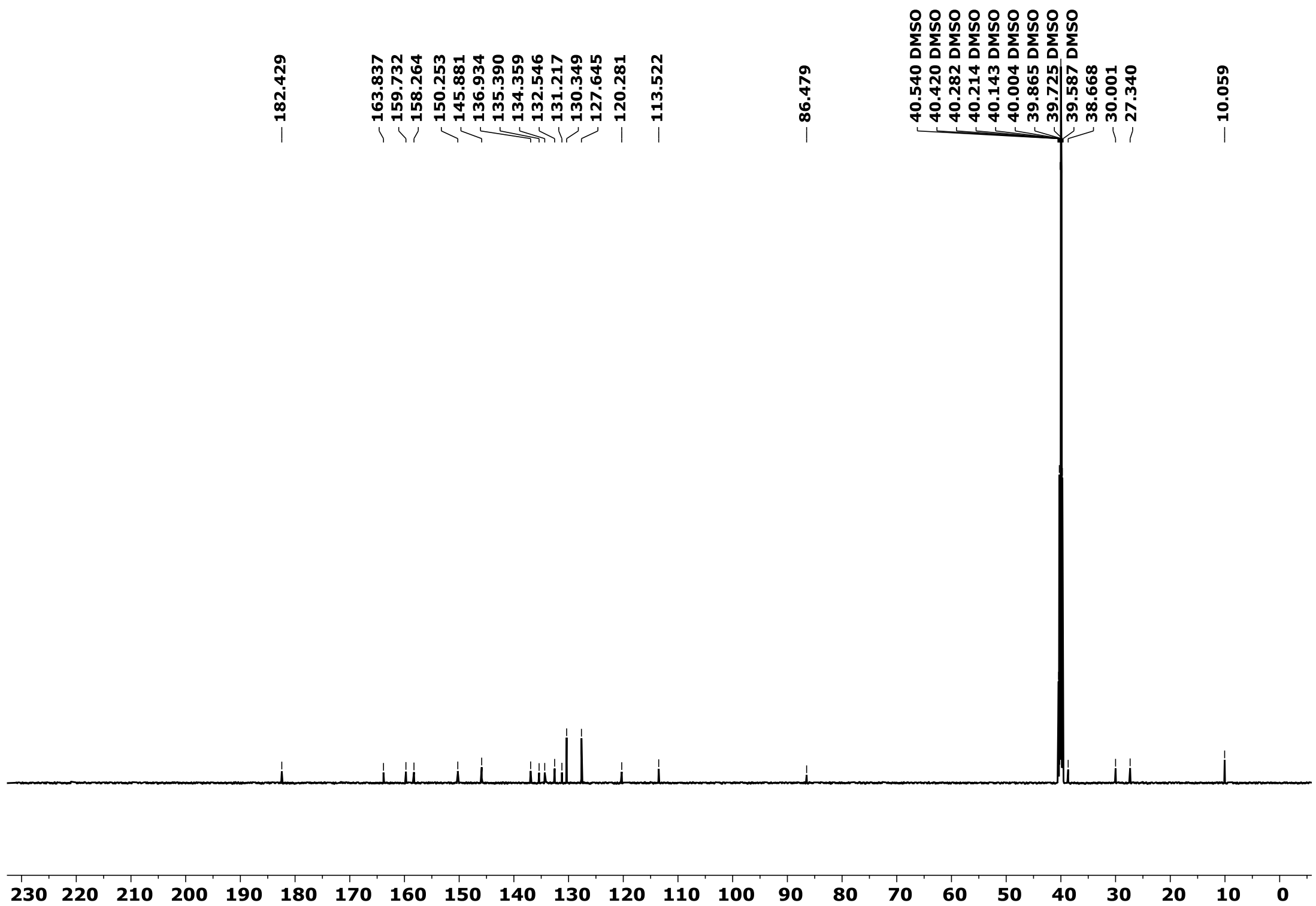

Figure S27. ^{13}C NMR (151 MHz, DMSO) spectrum of compound **9h**

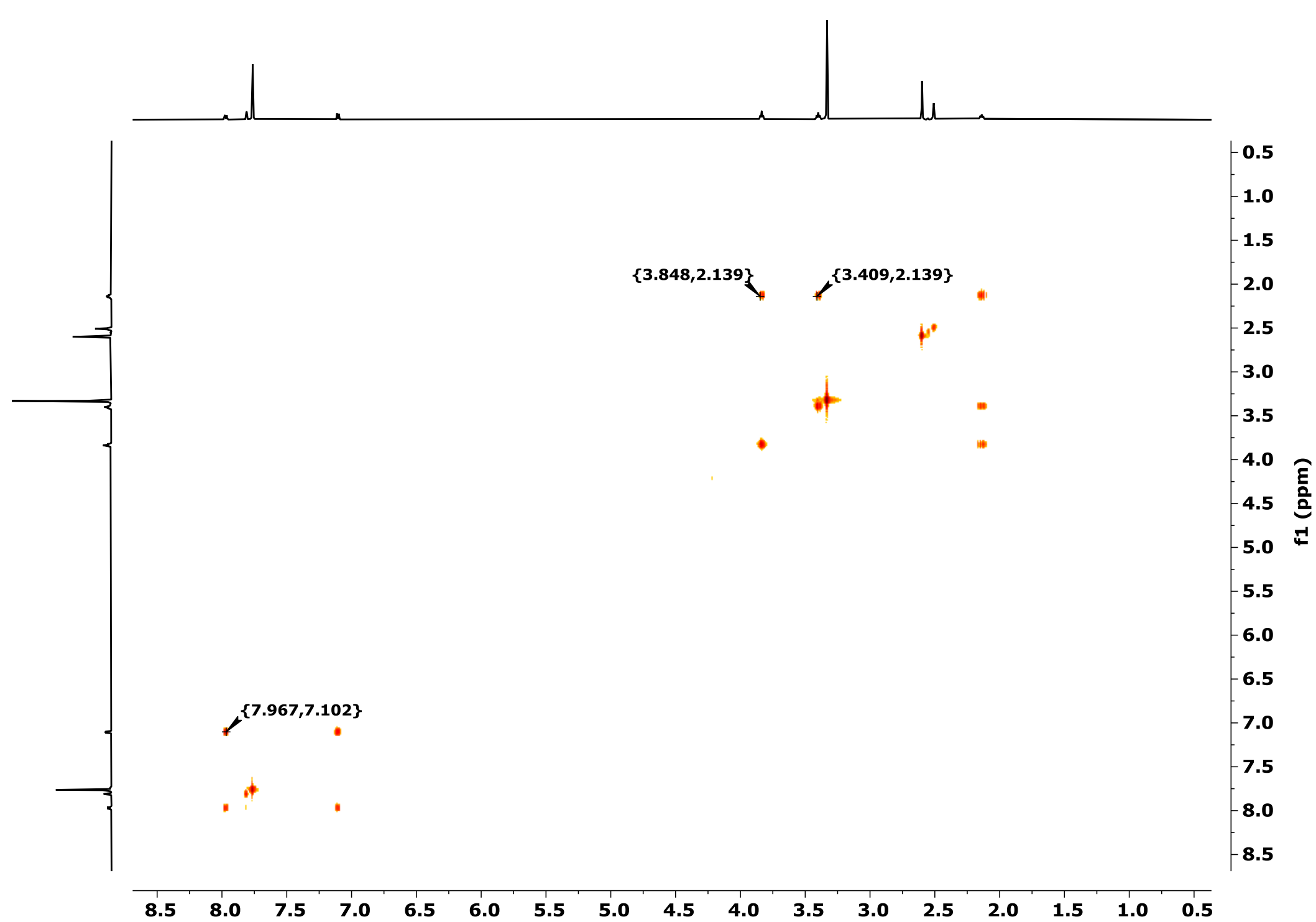

Figure S28. ^{1}H-^{1}H COSY spectrum (600 MHz, DMSO-d_6) of compound **9h**

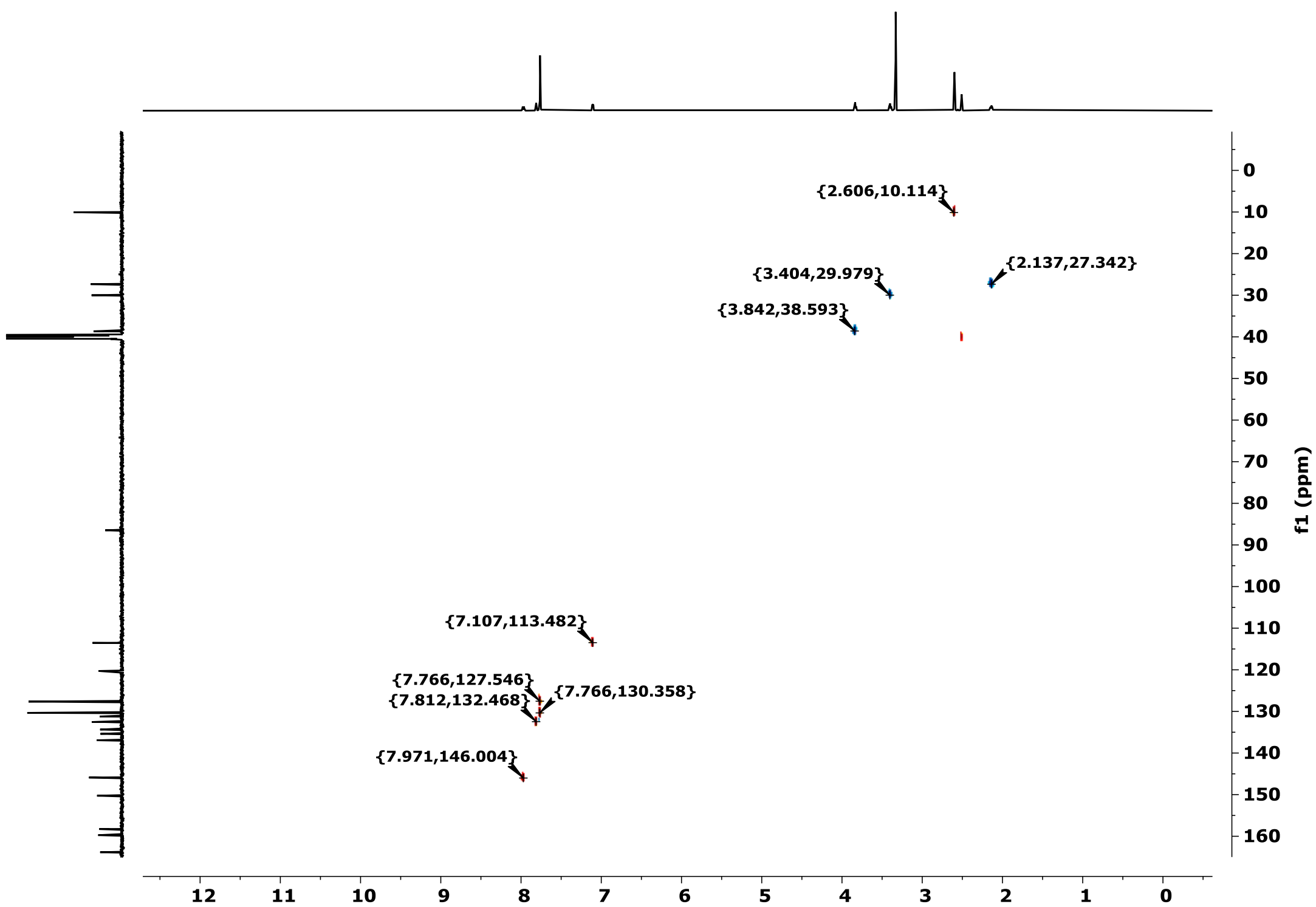

Figure S29. 2D ^{1}H-^{13}C HMQC spectrum (600/151 MHz, DMSO-d_6) of compound **9h**

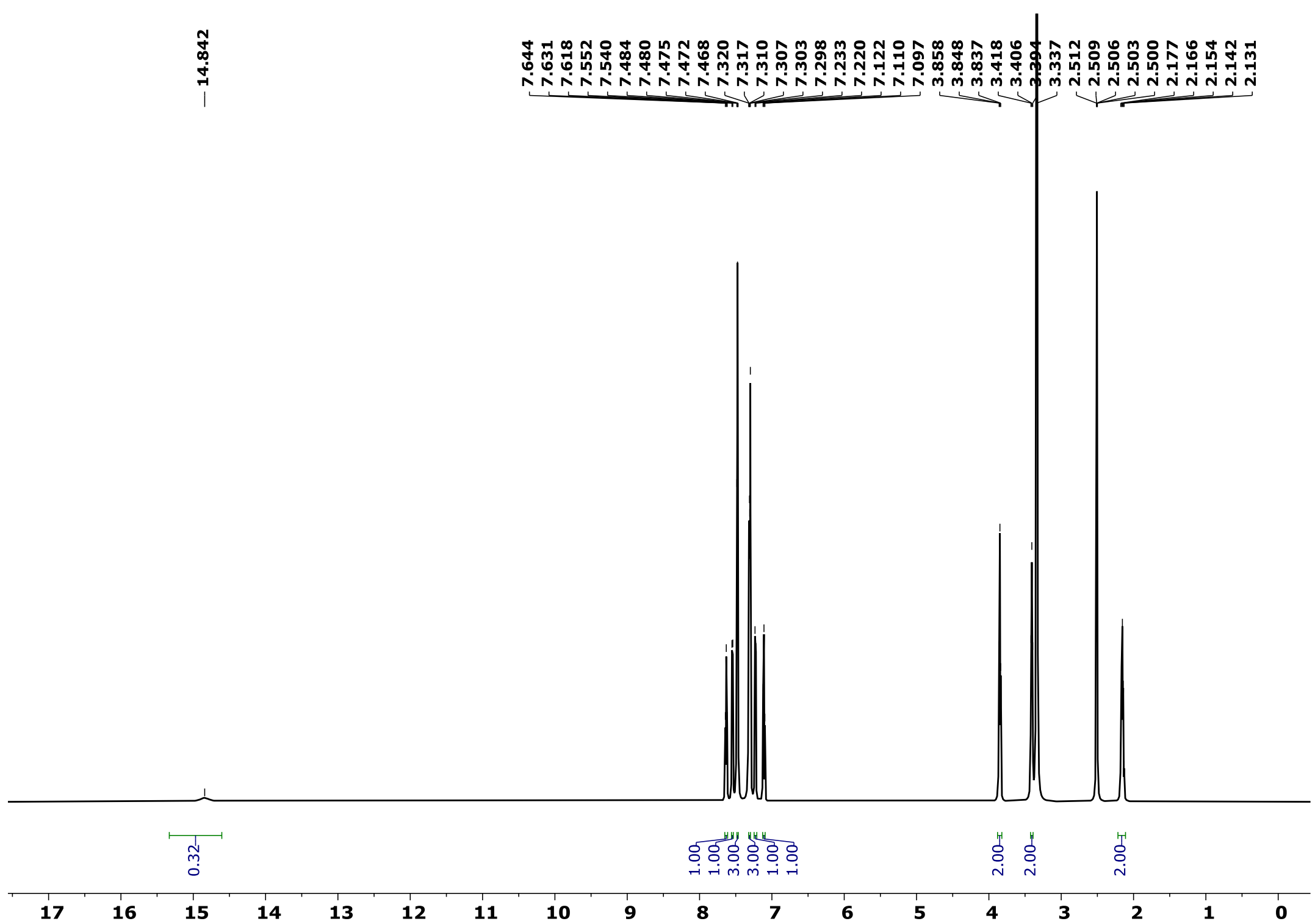

Figure S30. ^{1}H NMR (600 MHz, DMSO-d_6) spectrum of compound **15a**

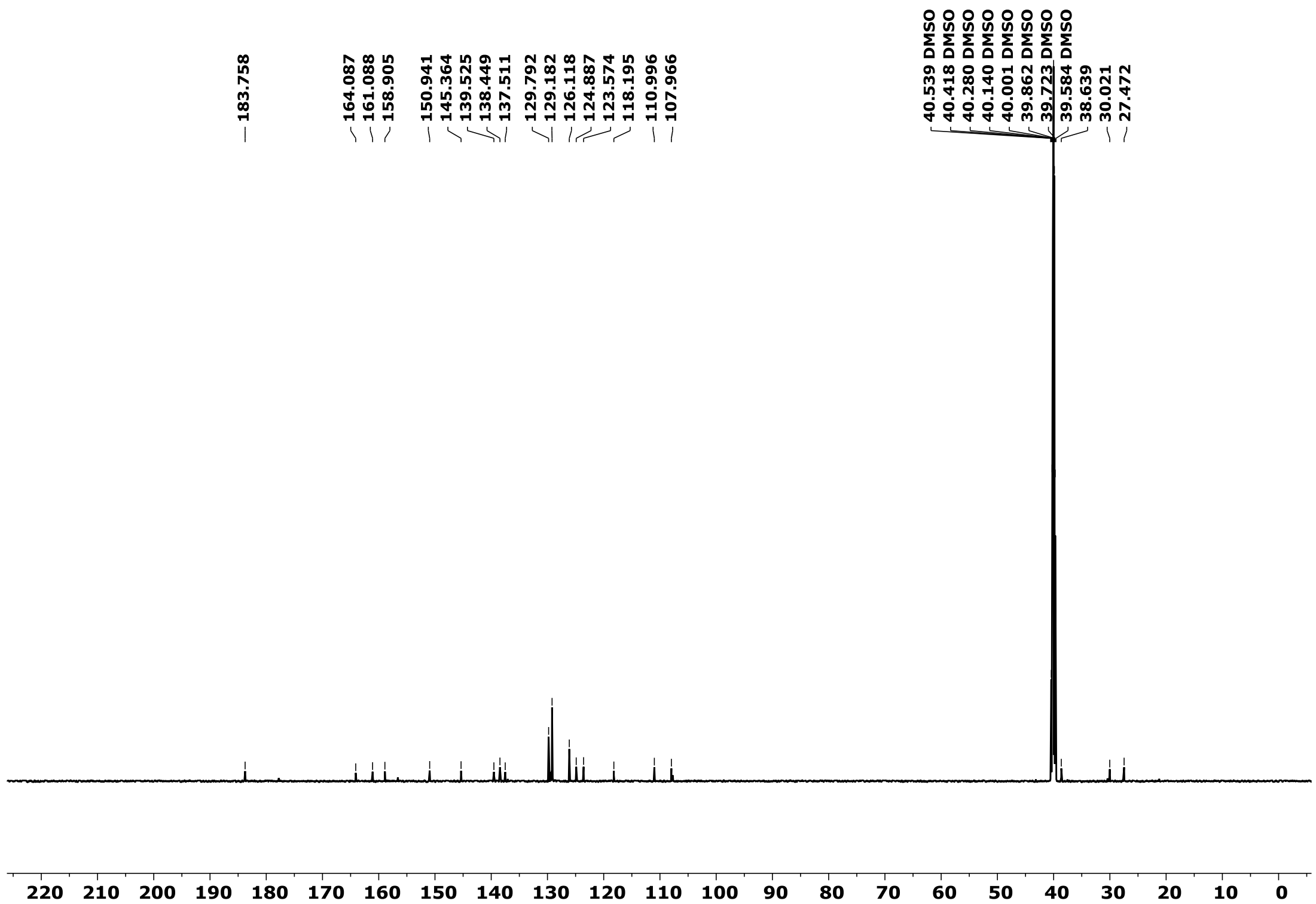

Figure S31. ^{13}C NMR (151 MHz, DMSO) spectrum of compound **15a**

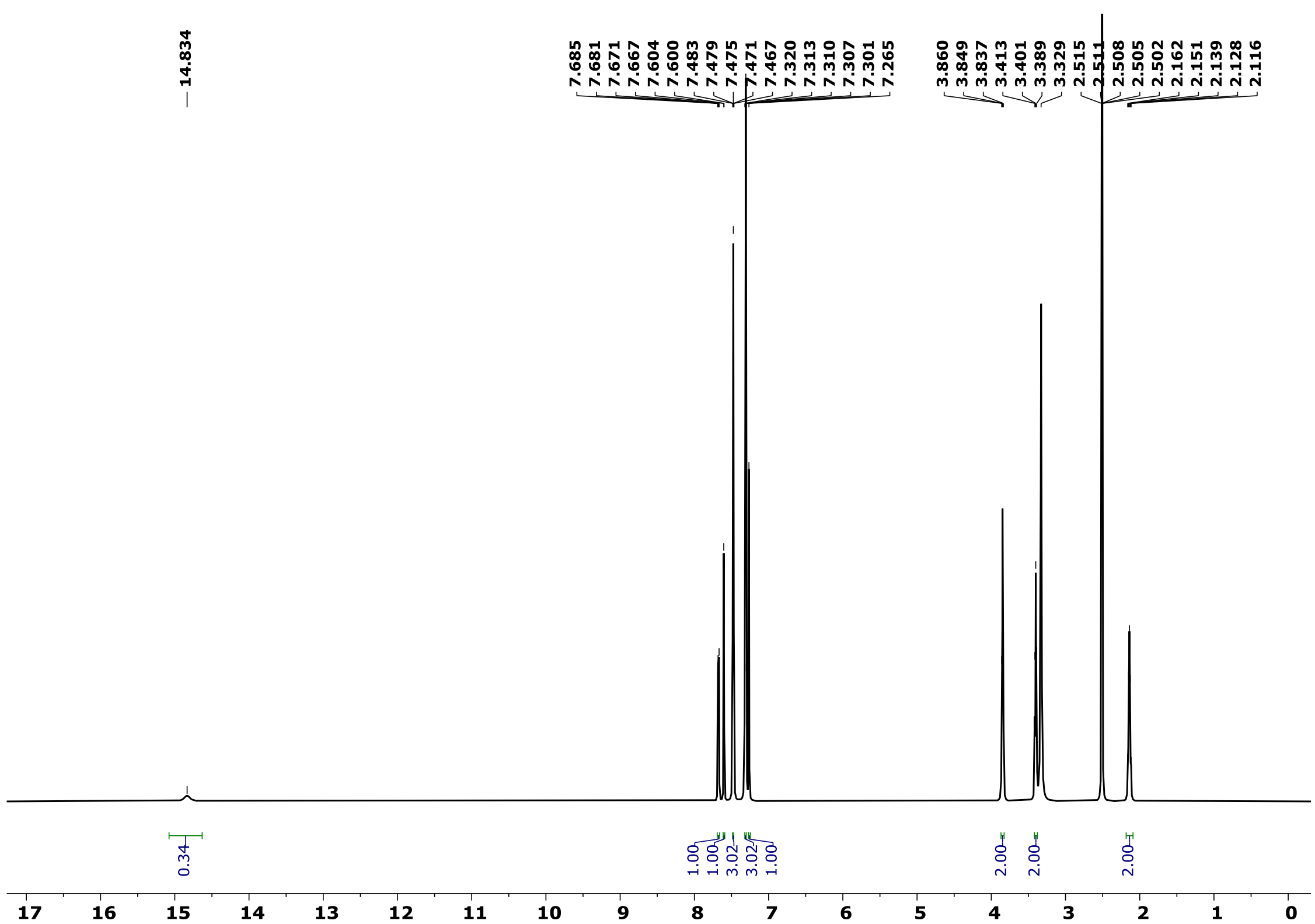

Figure S32. ^{1}H NMR (600 MHz, DMSO-d_6) spectrum of compound **15b**

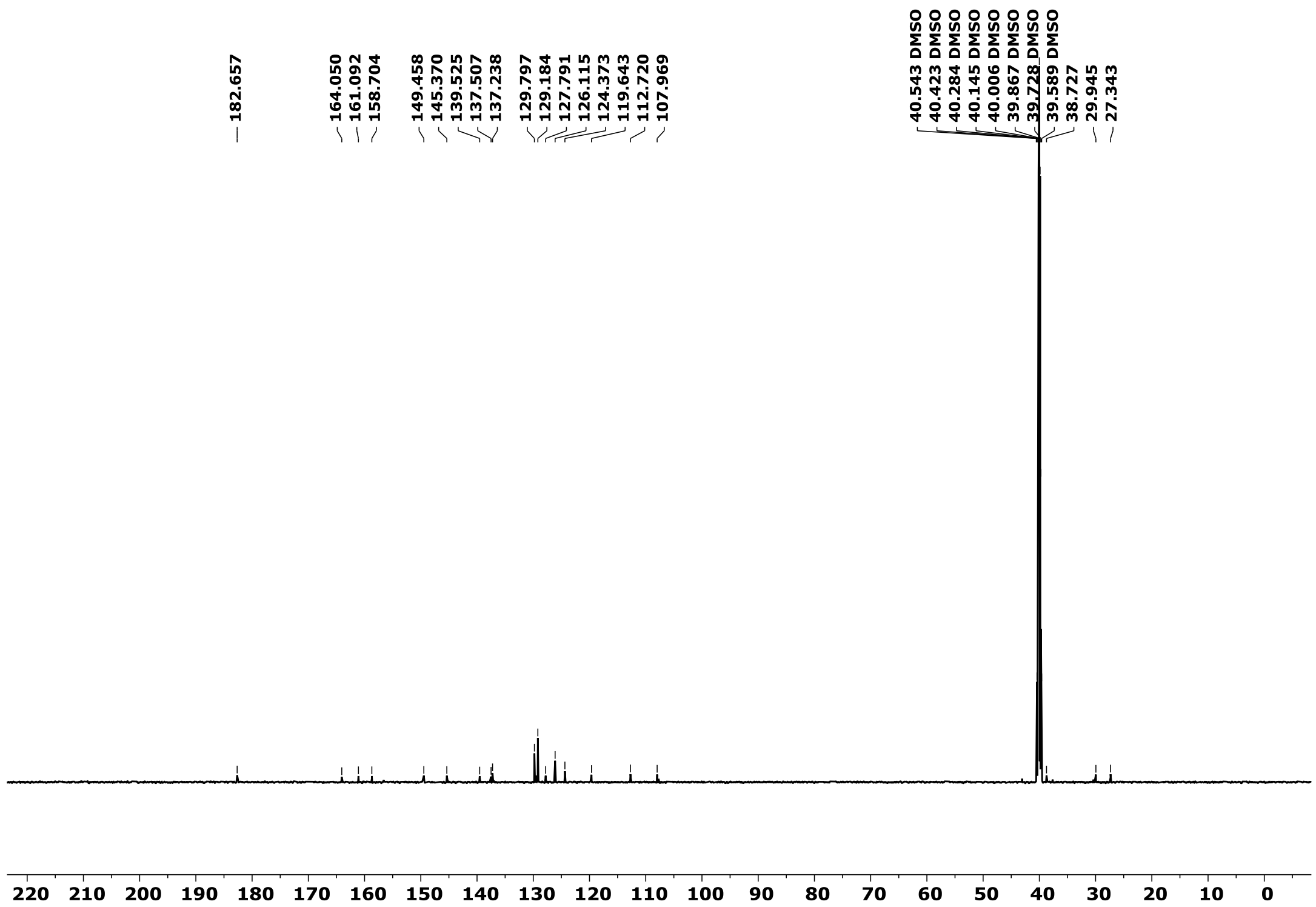

Figure S33. ^{13}C NMR (151 MHz, DMSO) spectrum of compound **15b**

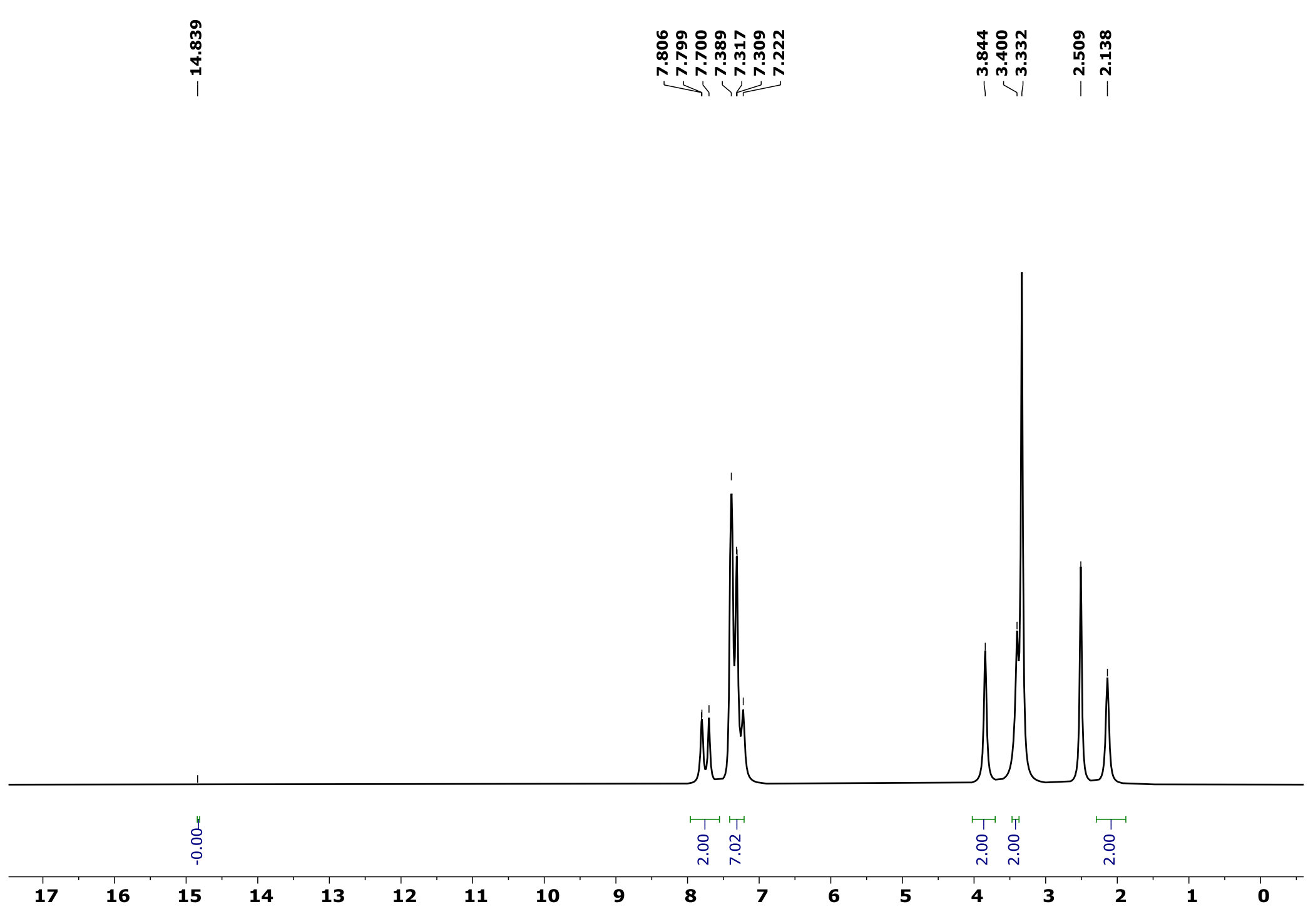

Figure S34. ^{1}H NMR (600 MHz, DMSO-d_6) spectrum of compound **15c**

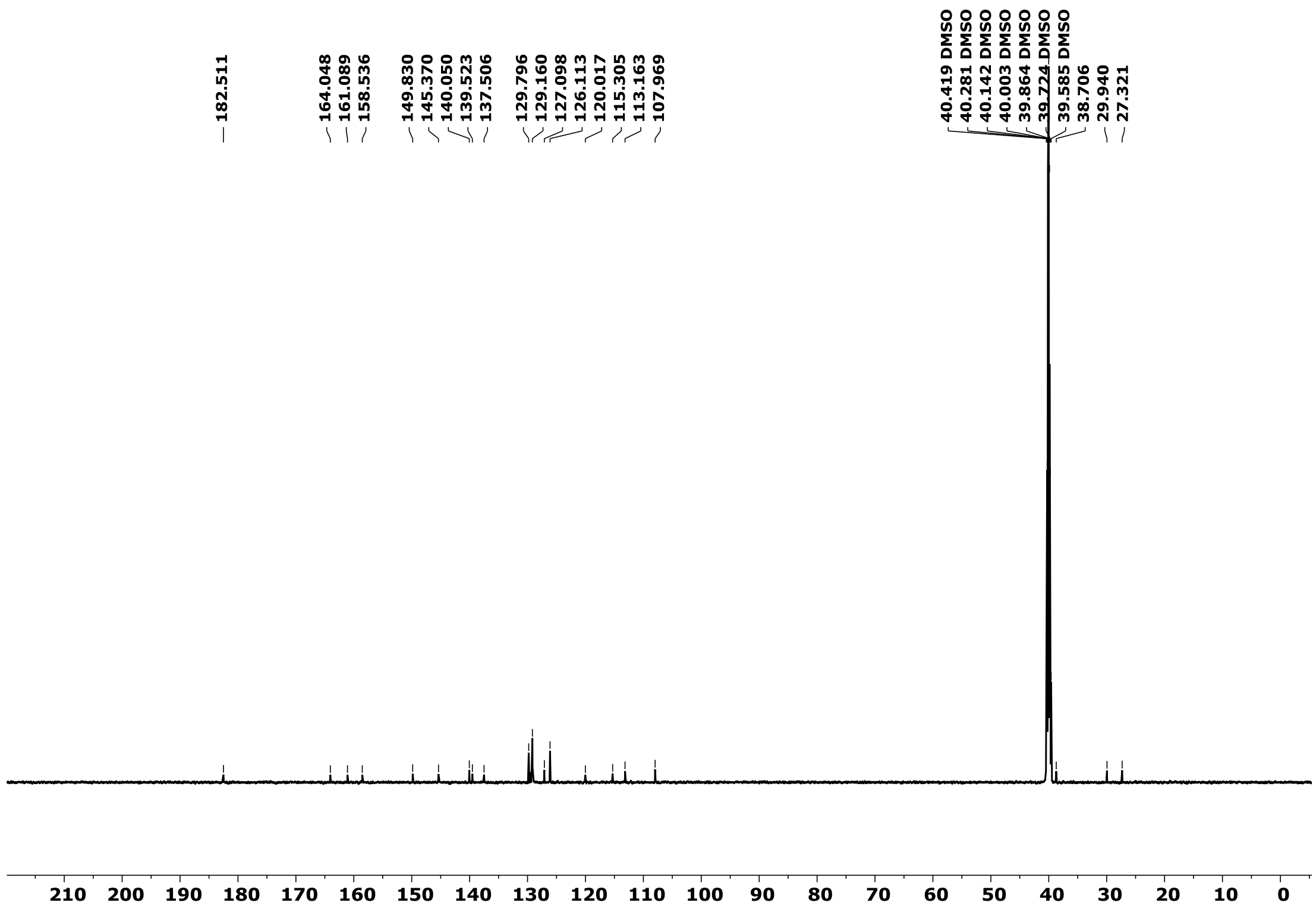

Figure S35. ^{13}C NMR (151 MHz, DMSO) spectrum of compound **15c**

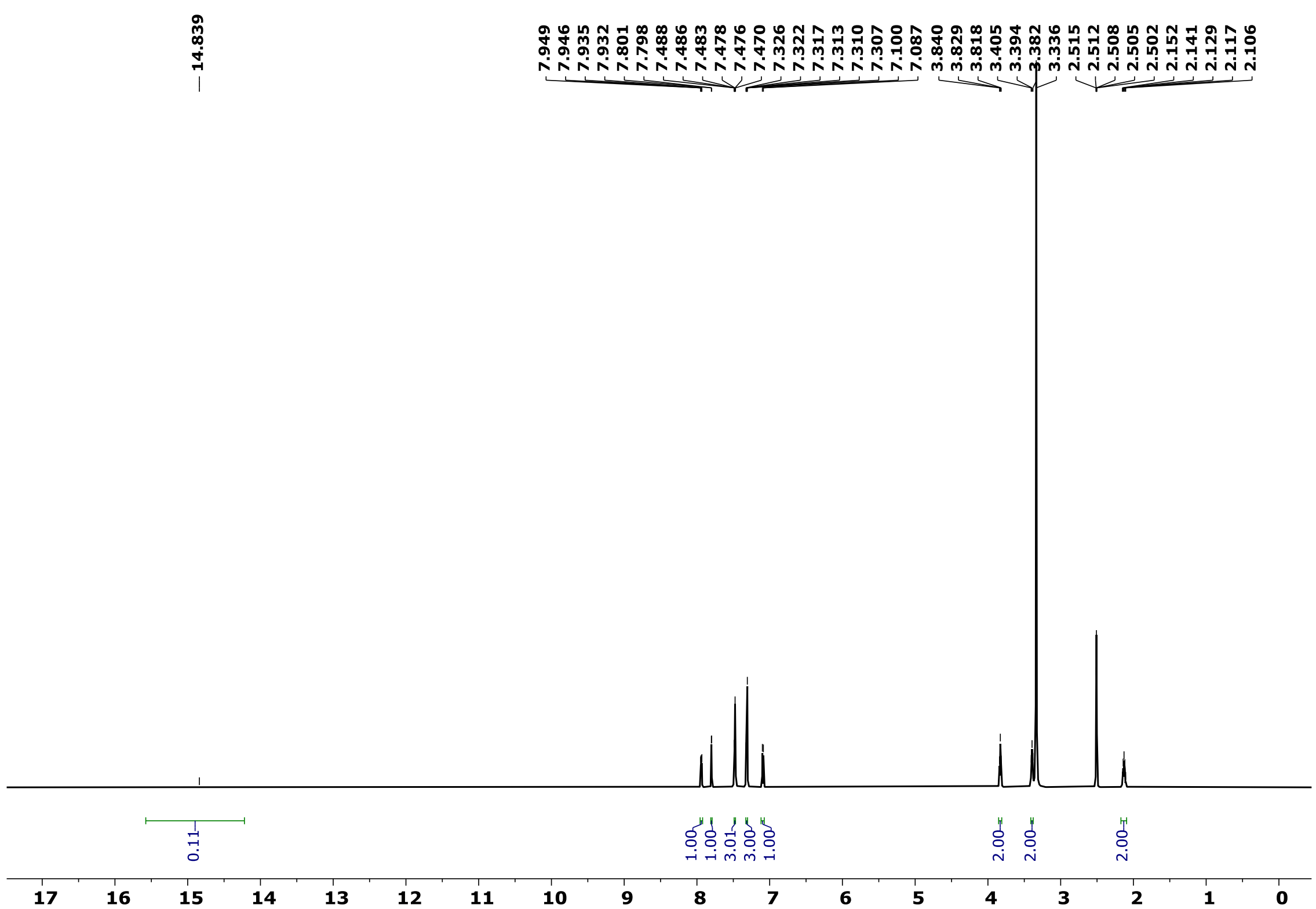

Figure S36. ^{1}H NMR (600 MHz, DMSO-d_6) spectrum of compound **15d**

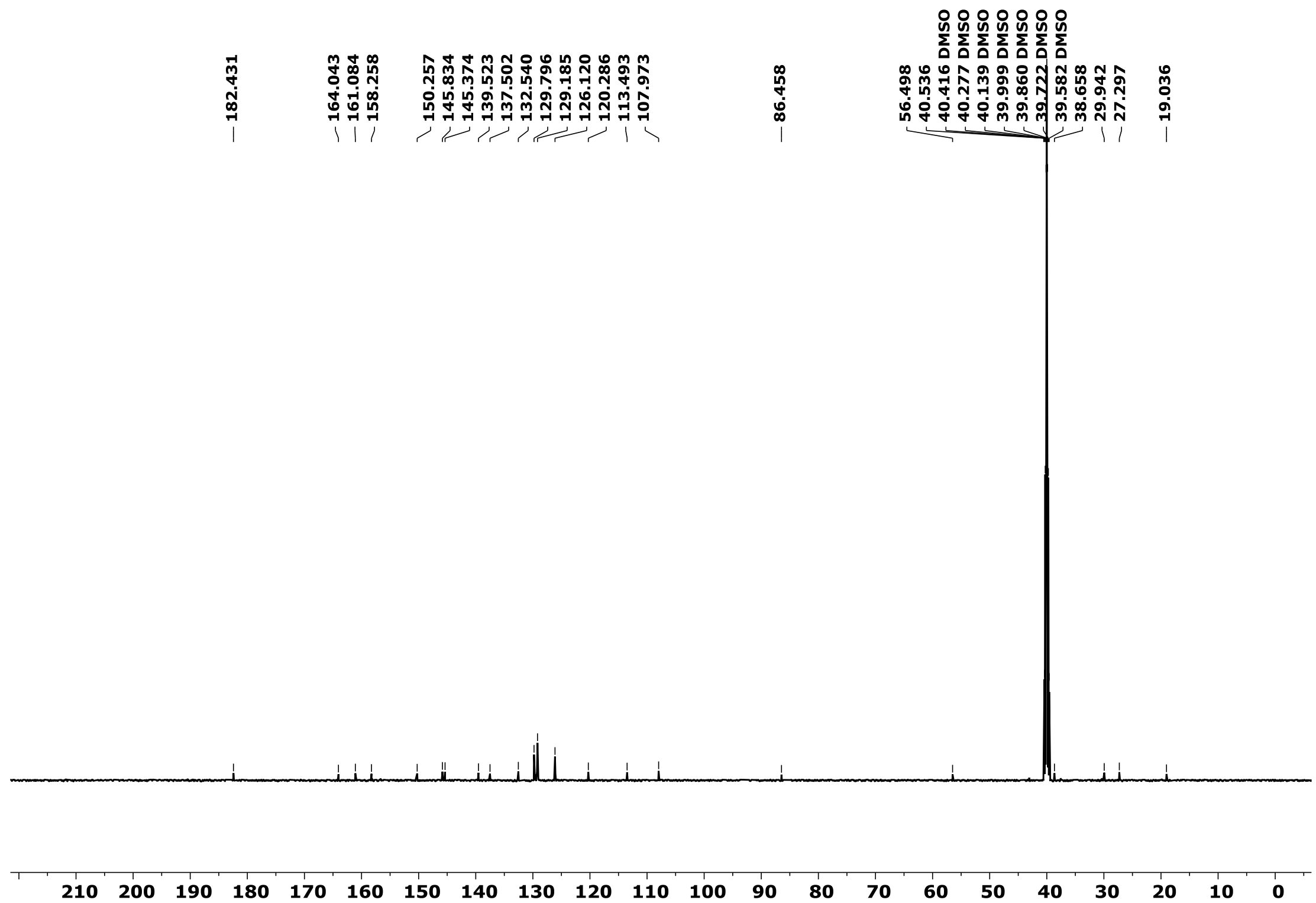

Figure S37. ^{13}C NMR (151 MHz, DMSO) spectrum of compound **15d**

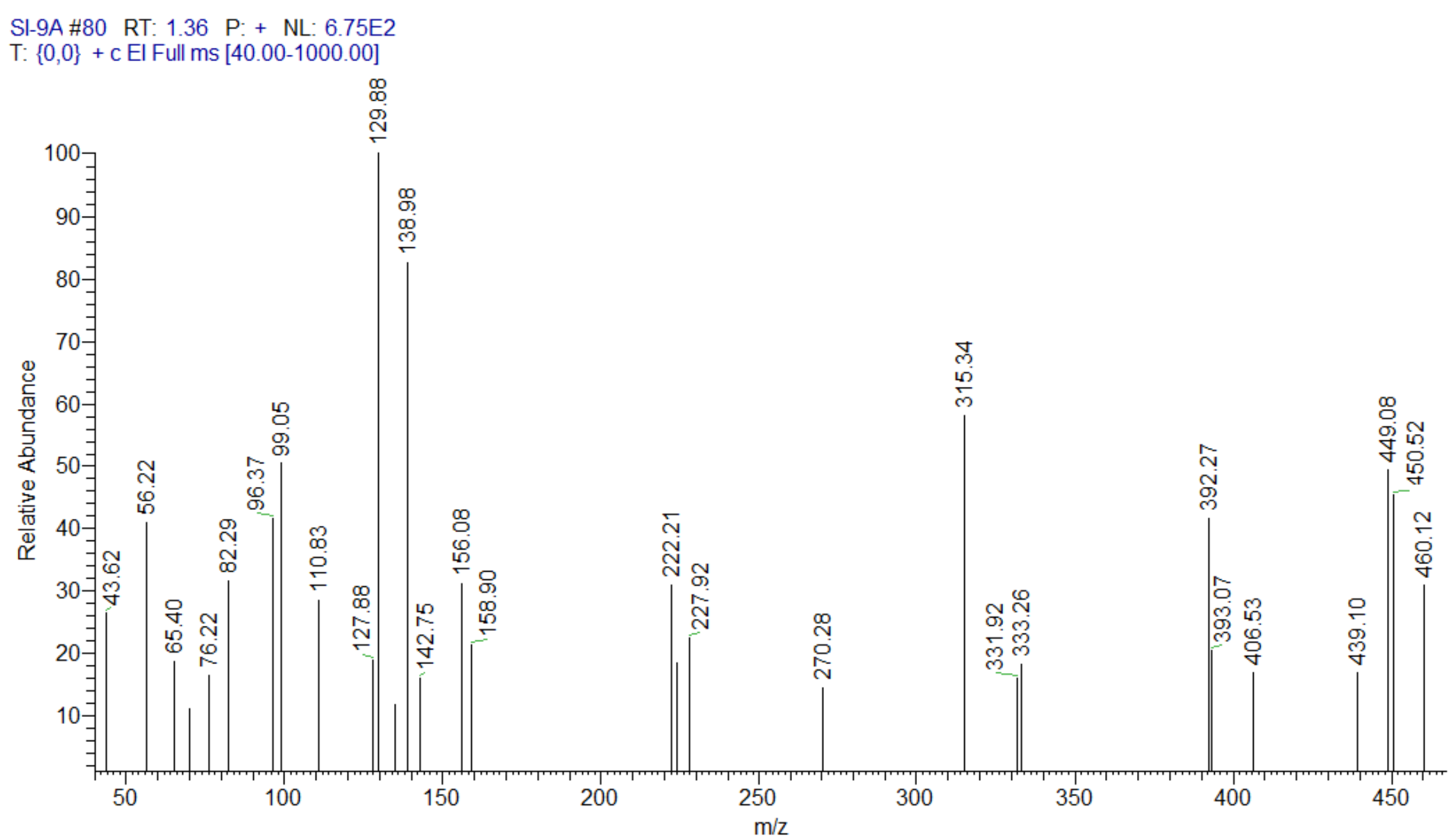

Figure S38. Mass spectrum of compound **9a**

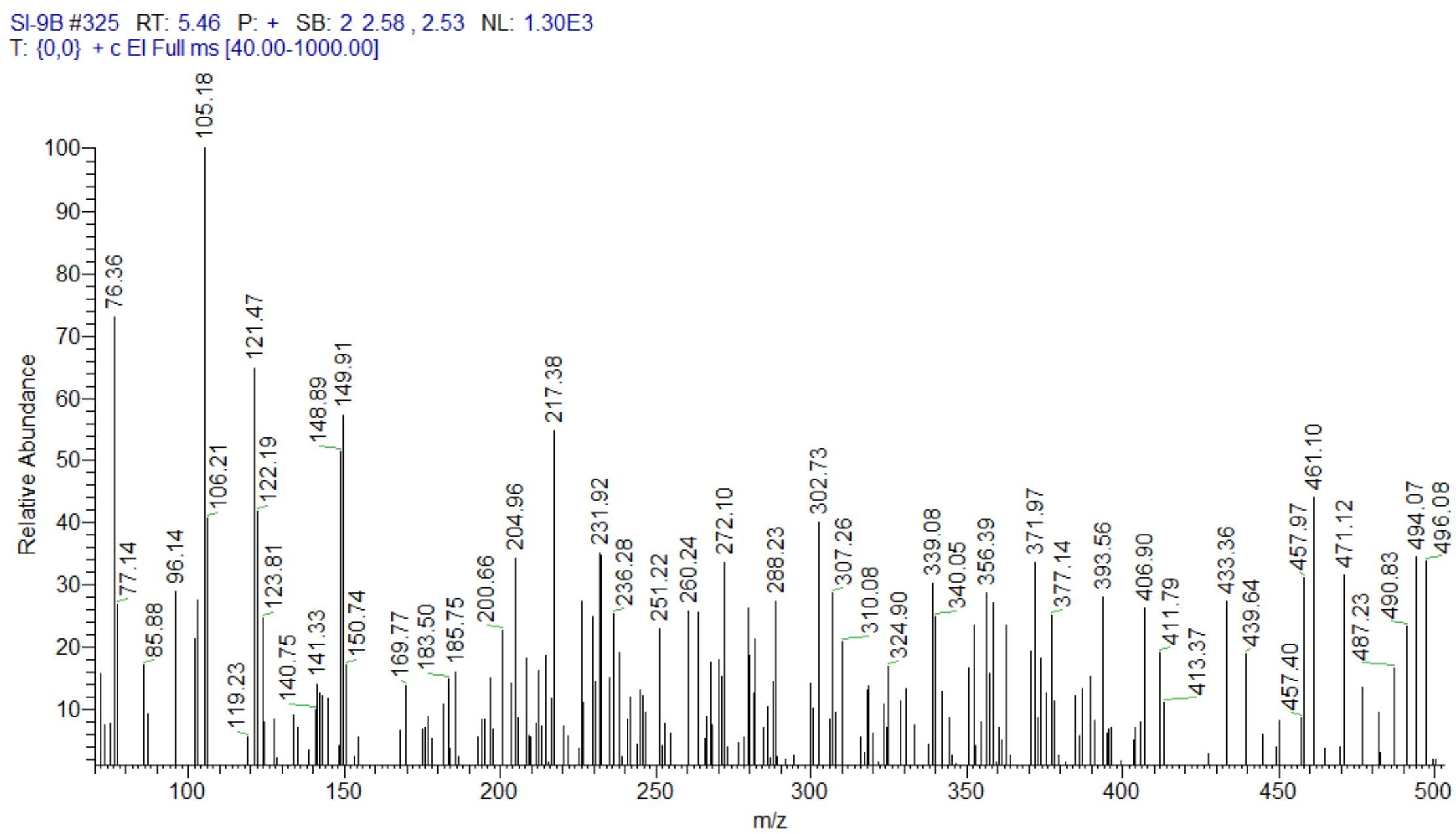

Figure S39. Mass spectrum of compound **9b**

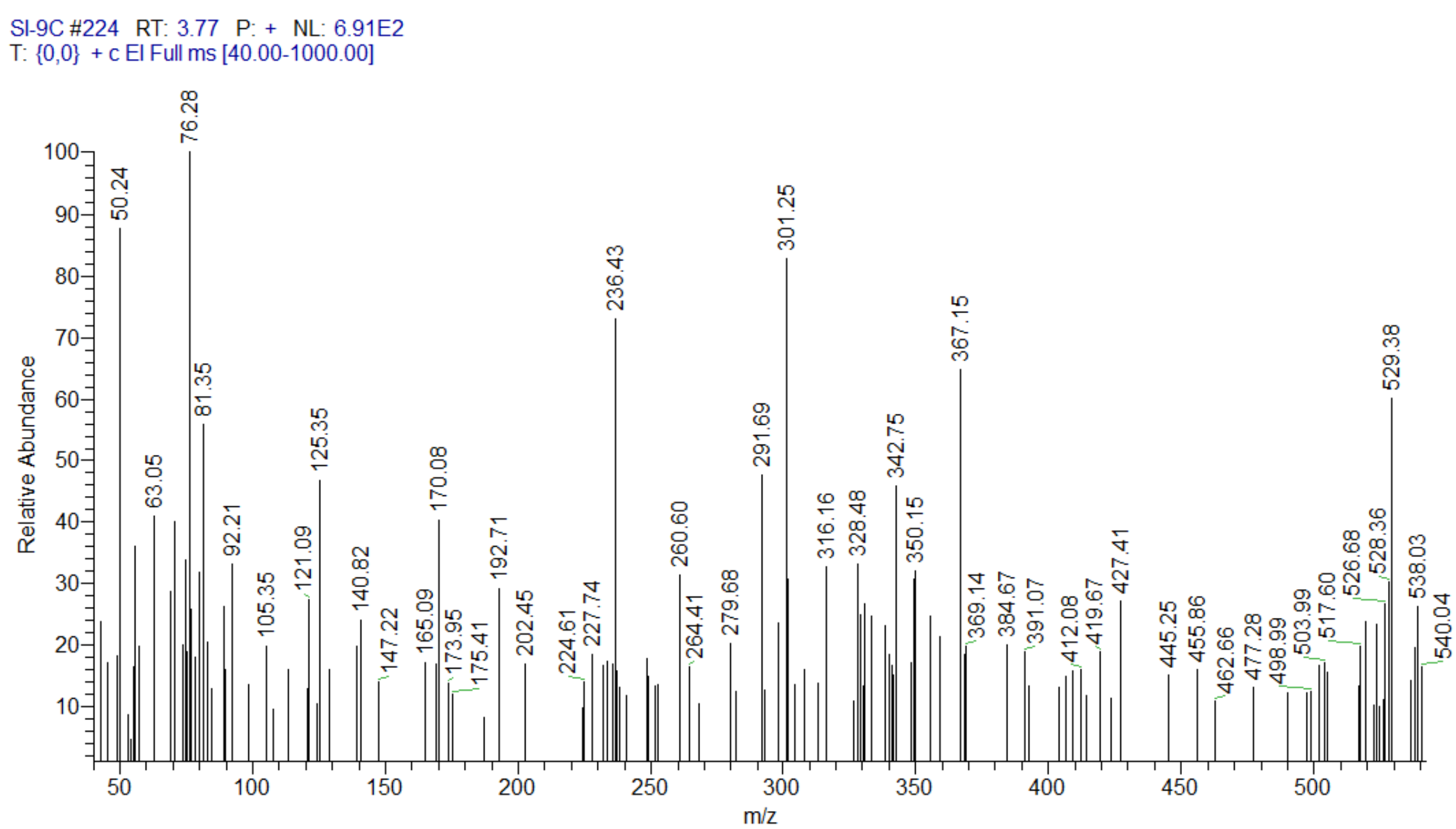

Figure S40. Mass spectrum of compound **9c**

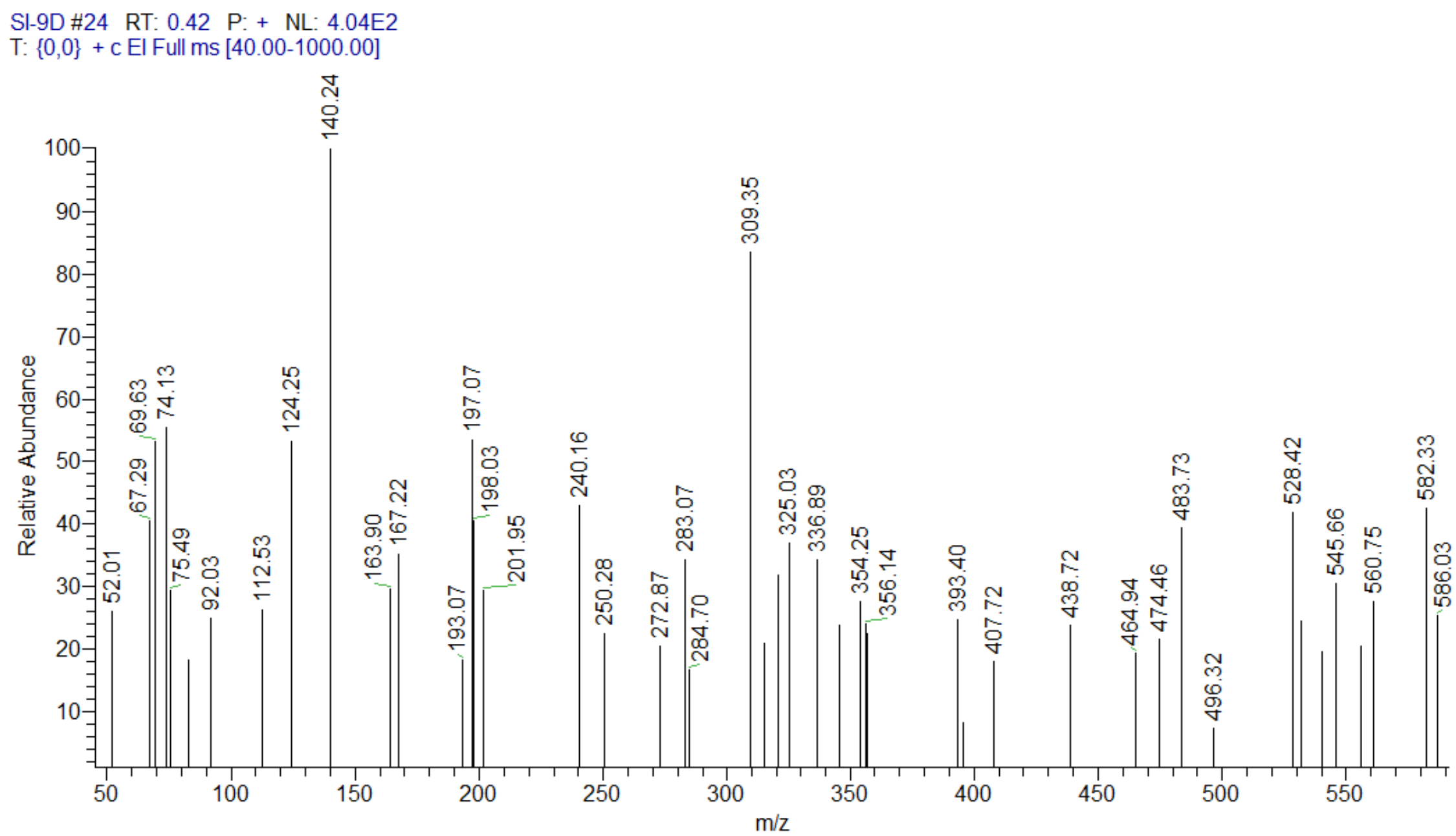

Figure S41. Mass spectrum of compound **9d**

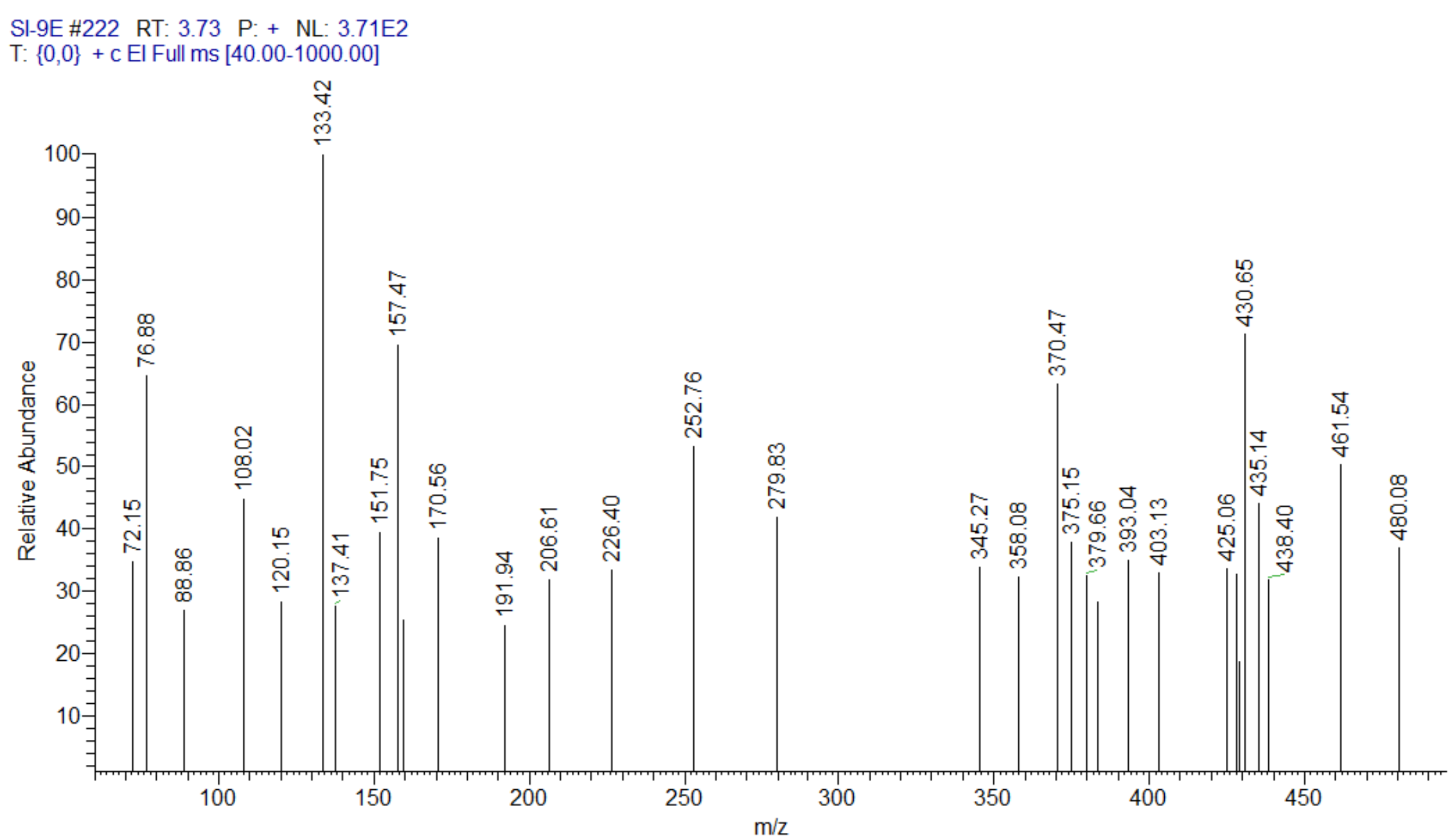

Figure S42. Mass spectrum of compound **9e**

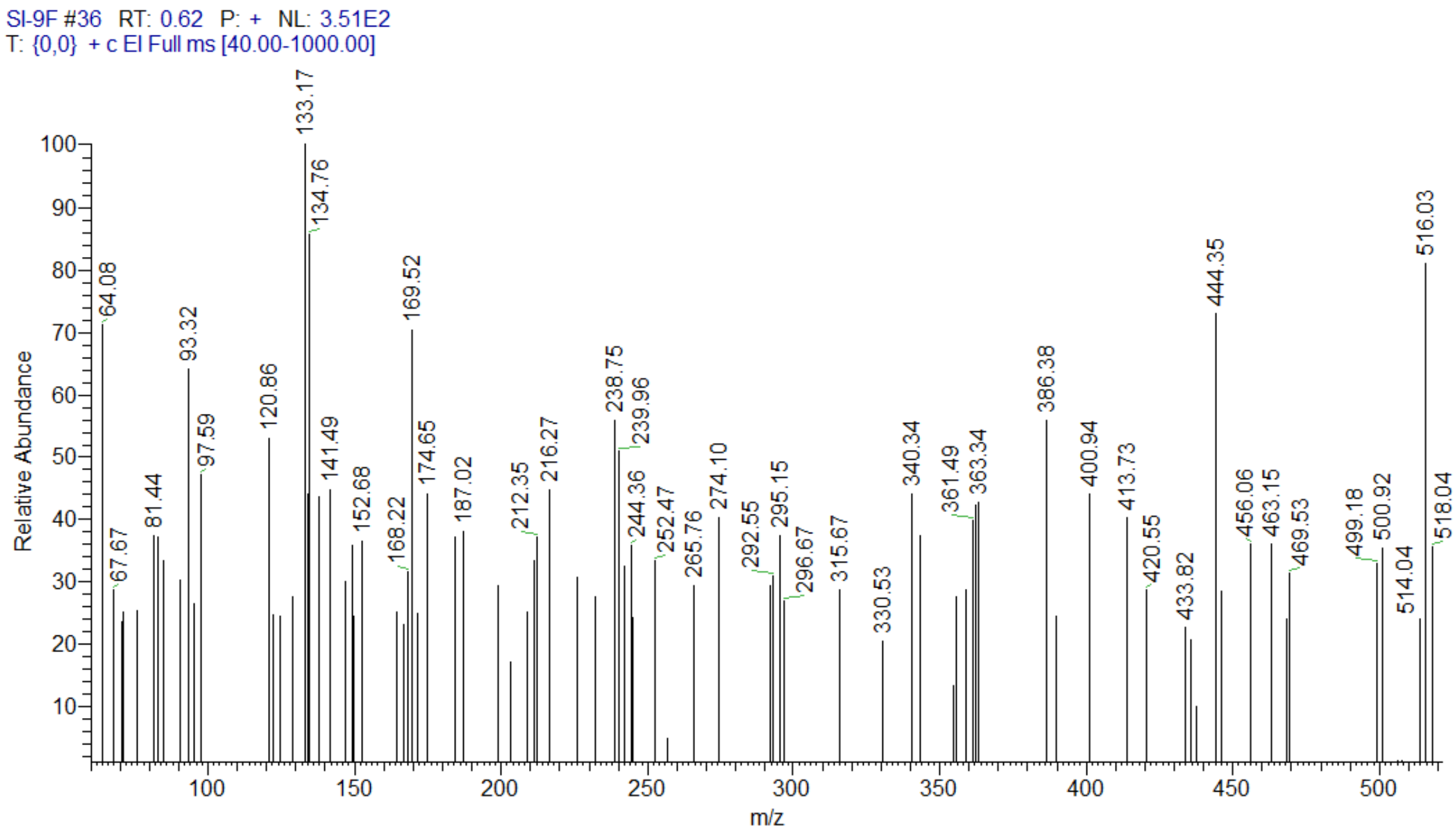

Figure S43. Mass spectrum of compound **9f**

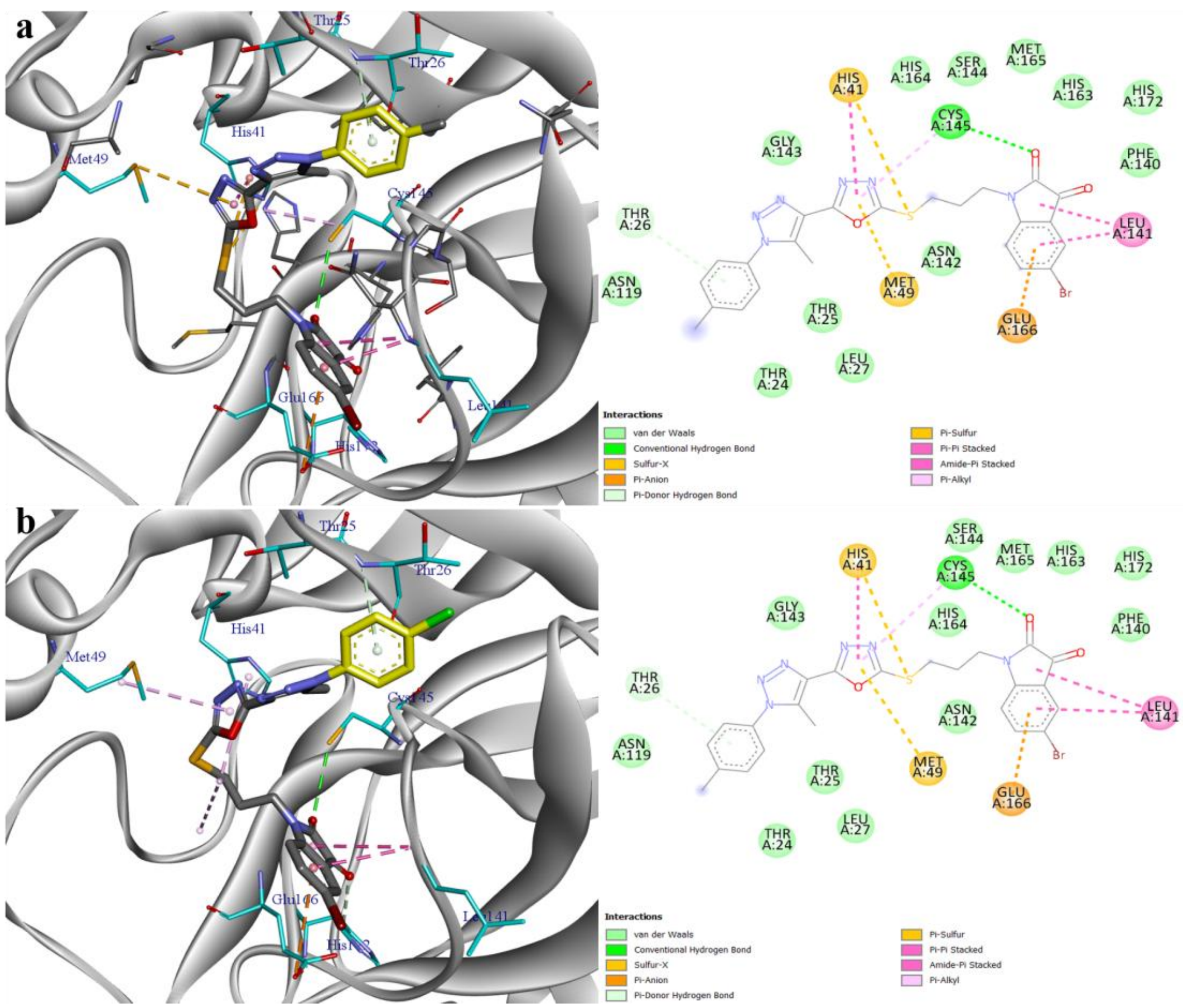

Figure S44. Presentation of the best docking pose through active site view (left) and 2D interaction plot (right) for (a) **9c** and (b) **9g** inside SARS-CoV-2 M^{pro} protein.

Table S1. ESI-MS characterization data of the synthesized compounds

Compound	Calculated M^+	Found M^+
**9a**	460.13	460.12
**9b**	494.09	494.07
**9c**	538.04	538.03
**9d**	586.02	586.03
**9e**	480.07	480.08
**9f**	514.03	514.04
**9g**	557.98	557.99
**9h**	605.97	605.95
**15a**	431.10	431.12
**15b**	465.06	465.05
**15c**	509.01	509.00
**15d**	557.00	556.99

Table S2. Reagent composition of the FRET-based SARS-CoV-2 M^{pro} inhibition assay.

Component	Primary stock	Intermediate (working) stock	Volume	Final conc. (100 µL)
Assay buffer (Tris-HCl, pH 7.4)	40 mM		50 µL	20 mM
Ultra-pure H_2O			10 µL	
SARS-CoV-2 M^{pro}	1–2 mg/ml (≈30-60 µM, batch-dependent)	3.70 µM (in assay buffer)	10 µL	370 nM
Test compound	10 mM in 100% DMSO	1 mM (in 25% DMSO)	10 µL	100 µM
FRET substrate	10 mM	100 µM (in H_2O)	20 µL	20 µM
Total			100 µL	

Table S3. Global nonlinear model comparison and residual assessment for SARS-CoV-2 M^{pro} inhibition by compounds **15a-c**.

Compound	Best-supported kinetic models	ΔAICc between noncompetitive and mixed models	Mixed-model α (95% CI)	Residual assessment	Interpretation
**15a**	Noncompetitive / Mixed	1.29	0.41 (0.06-3.01)	No pronounced systematic trend with substrate concentration or fitted velocity	Noncompetitive/ mixed
**15b**	Noncompetitive / Mixed	0.46	0.44 (0.12-1.56)	No pronounced systematic trend with substrate concentration or fitted velocity	Noncompetitive/ mixed
**15c**	Mixed / Noncompetitive	0.85	0.41 (0.15-1.14)	No pronounced systematic trend with substrate concentration or fitted velocity	Mixed/ noncompetitive
